# Dysregulated Choline, Methionine, and Aromatic Amino Acid Metabolism in Patients with Wilson Disease: Exploratory Metabolomic Profiling and Implications for Hepatic and Neurologic Phenotypes

**DOI:** 10.3390/ijms20235937

**Published:** 2019-11-26

**Authors:** Tagreed A. Mazi, Gaurav V. Sarode, Anna Czlonkowska, Tomasz Litwin, Kyoungmi Kim, Noreene M. Shibata, Valentina Medici

**Affiliations:** 1 Department of Nutrition, University of California Davis, 3135 Meyer Hall, One Shields Avenue, Davis, CA 95616, USA; 2 Department of Community Health Sciences, College of Applied Medical Sciences, King Saud University, P.O. Box 10219, Riyadh 11433, Saudi Arabia; 3 Department of Internal Medicine, Division of Gastroenterology and Hepatology, University of California Davis, 4150 V Street, Suite 3500 Sacramento, CA 95817, USA; gsarode@ucdavis.edu (G.V.S.); nshibata@ucdavis.edu (N.M.S.); 4 Second Department of Neurology, Institute of Psychiatry and Neurology, 02 957 Warsaw, Poland; czlonkow@ipin.edu.pl (A.C.); tlitwin@ipin.edu.pl (T.L.); 5 Division of Biostatistics, Department of Public Health Sciences, University of California Davis, One Shields Avenue, Davis, CA 95616, USA; kmkim@ucdavis.edu

**Keywords:** copper, metabolomics, phospholipids, choline, phenylalanine, tyrosine, histidine, steatosis, neurotransmitters

## Abstract

Wilson disease (WD) is a genetic copper overload condition characterized by hepatic and neuropsychiatric symptoms with a not well-understood pathogenesis. Dysregulated methionine cycle is reported in animal models of WD, though not verified in humans. Choline is essential for lipid and methionine metabolism. Defects in neurotransmitters as acetylcholine, and biogenic amines are reported in WD; however, less is known about their circulating precursors. We aimed to study choline, methionine, aromatic amino acids, and phospholipids in serum of WD subjects. Hydrophilic interaction chromatography-quadrupole time-of-flight mass spectrometry was employed to profile serum of WD subjects categorized as hepatic, neurologic, and pre-clinical. Hepatic transcript levels of genes related to choline and methionine metabolism were verified in the Jackson Laboratory toxic milk mouse model of WD (tx-j). Compared to healthy subjects, choline, methionine, ornithine, proline, phenylalanine, tyrosine, and histidine were significantly elevated in WD, with marked alterations in phosphatidylcholines and reductions in sphingosine-1-phosphate, sphingomyelins, and acylcarnitines. In tx-j mice, choline, methionine, and phosphatidylcholine were similarly dysregulated. Elevated choline is a hallmark dysregulation in WD interconnected with alterations in methionine and phospholipid metabolism, which are relevant to hepatic steatosis. The elevated phenylalanine, tyrosine, and histidine carry implications for neurologic manifestations and are worth further investigation.

## 1. Introduction

Wilson disease (WD) is caused by copper overload mainly in the liver and brain as a consequence of mutations affecting the P-type ATPase transporter responsible for copper excretion into the biliary system and ceruloplasmin maturation through the trans-Golgi network [1,2]. The clinical presentation of WD is highly variable and includes hepatic and neuropsychiatric manifestations [2]. Hepatic involvement ranges from asymptomatic hepatomegaly to hepatic steatosis, hepatitis, fibrosis and cirrhosis, liver failure, and hepatocellular carcinoma [3,4]. Neuropsychiatric symptoms include tremors, dystonia, bradykinesia, hypomnesia, and dysgnosia [5]. The molecular basis of WD is not understood. A major role is attributed to oxidative stress and the production of reactive oxygen species (ROS) with consequent damage to cellular proteins, lipids, and nucleic acids, and effects on mitochondria morphology and bioenergetics [6,7]. However, knowledge is still lacking about systemic WD manifestations and if and how they are metabolically interconnected.

Choline is an essential nutrient functioning at the intersection of multiple metabolic pathways. Its oxidized form, betaine, is relevant to the methionine cycle as a methyl donor in homocysteine re-methylation reactions catalyzed by betaine-homocysteine methyltransferase (BHMT) to generate methionine [8]. We and others have previously reported dysregulation in the methionine cycle in an animal model of WD, primarily in relation to epigenetic mechanisms [9,10,11]; however, this is not clear in human subjects with WD. Choline is also required for phospholipid (PL) synthesis, including phosphatidylcholines (PCs) and sphingolipids (SLs); therefore, it is essential for bio-membrane integrity, mitochondrial bioenergetics, and lipid and bile metabolism [12,13,14]. PC is the predominant PL in mammalian cell membranes, synthesized mainly via the choline-dependent cytidine-diphosphate-choline (CDP-choline) pathway with contributions from the liver-specific phosphatidylethanolamine *N*-methyltransferase (PEMT) pathway [12]. The PEMT pathway requires the methyl donor S-adenosylmethionine (SAM) and is inhibited by elevated S-adenosylhomocysteine (SAH) [15,16]. Dysregulation in PL is implicated in hepatic steatosis, inflammation, and many neurologic conditions [17,18,19,20]. Choline deficiency results in steatosis due to impaired hepatic synthesis of very-low-density lipoproteins (VLDLs) and export of triglycerides [21].

An additional potential layer of metabolic interaction is provided by the bioactive lipids ceramide, SLs, and sphingosine-1-phosphate (S1P). PC and ceramide are precursors of sphingomyelins (SMs), the main SLs in mammalian membranes [22]. S1P is formed from sphingosine, the degradation product of ceramide [23]. Sphingosine, S1P, and ceramide are interconvertible, and their homeostasis determines the cell’s fate. The formation of S1P promotes cell growth and survival, while sphingosine and ceramide induce apoptosis [24,25].

Acetylcholine and the biogenic amines, histamine, dopamine, norepinephrine, epinephrine, and serotonin are endogenous chemical messengers responsible for neural transmission and regulating locomotion, muscle tone, mood, attention, and behavior [26,27,28]. Choline is the precursor for acetylcholine; and neuroactive biogenic amines are derived from the aromatic amino acids phenylalanine, tyrosine, tryptophan, and histidine [29,30]. Methionine, via SAM formation, is also required for the synthesis of serotonin, epinephrine, and dopamine [31].

Metabolomic approaches offer a powerful tool to understand metabolism in biological systems. Most of the available metabolomic data in WD are from animal models [32,33,34]. In a previous metabolomic analysis of WD patients, we reported elevated sorbitol, alterations in tricarboxylic acid cycle intermediates, and some amino acids [35]. Given the scarce human metabolomic studies in WD and available data on altered methionine metabolism in animals, we explored serum choline, amino acids, and PL levels in subjects with WD compared to healthy controls (HC), hypothesizing that metabolomics patterns could further differentiate WD clinical presentations. We also aimed to verify hepatic transcript levels of key genes related to choline and methionine metabolism in the Jackson toxic milk mouse, an animal model of WD, to ultimately provide new insights into WD pathogenesis.

## 2. Results

### 2.1. Natural Clustering

Of the 374 detected metabolites, 107 were identified and annotated. Xenobiotics were excluded and 97 metabolites were included in the analysis. Principle component analysis score plot based on detected metabolites showed clustering with some overlap, suggesting a metabolomic variance may distinguish between WD and HC groups (Figure S1).

### 2.2. Identification of Distinctive Metabolites in Serum of WD Subjects Compared to HC

**Figure 1:**
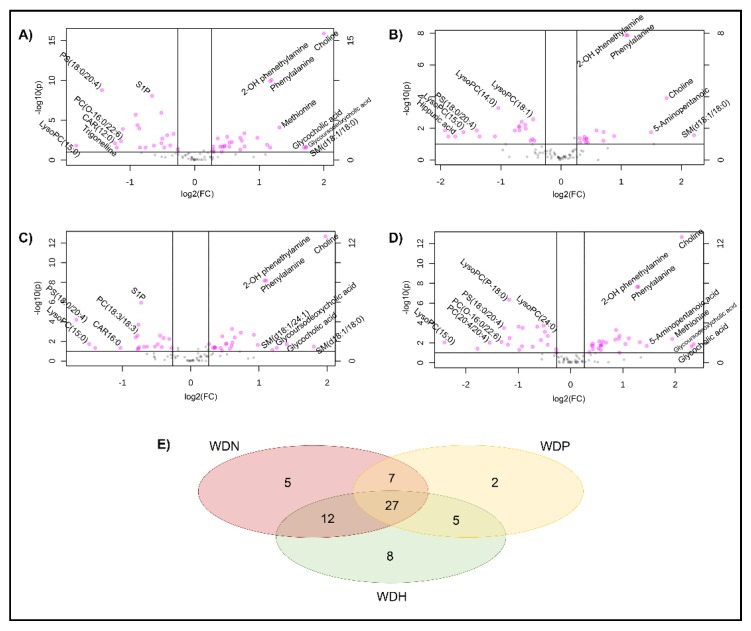
Volcano plot for Wilson disease (WD) and WD subgroups compared to healthy control (HC) based on all annotated metabolites. (**A**) WD (all); (**B**) WD pre-clinical (WDP); (**C**) WD neurologic (WDN); (**D**) WD hepatic (WDH). Important features were selected with fold change (FC) threshold 1.2 and t-test (*p*) threshold 0.1. Both fold changes and *p*-values are log-transformed. The pink circles represent features above the threshold. The further the circle’s position away from (0,0), the more significant the feature. (**E**) Venn diagram displaying common and group-specific significant metabolites in WD subgroups compared to HC.

**Figure 2:**
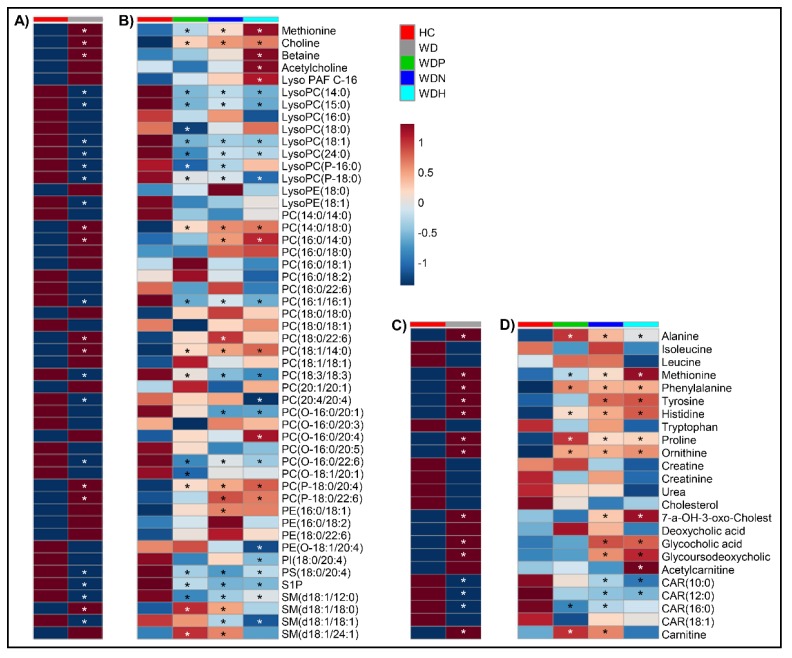
Heat-map of selected metabolites. Averages for each metabolite by group are shown to indicate fold-change magnitude and direction. Choline and related metabolites, methionine, and phospholipids in (**A**) healthy control (HC) compared to Wilson disease (WD); and (**B**) HC compared to WD pre-clinical (WDP), WD neurologic (WDN), and WD hepatic (WDH). Amino acids, cholesterol and bile, acylcarnitines, and related metabolites in (**C**) HC compared to WD; and (**D**) HC compared to WDP, WDN, and WDH. Metabolites marked by asterisks indicate FDR-adjusted *p* < 0.1 compared to HC by volcano analysis.

Volcano analysis comparing WD to HC revealed 158 significant metabolites, among which 44 were identified and annotated. A detailed list of all detected metabolites with statistical significance after false discovery rate (FDR) adjustment is shown in Figure S2 and Table S1. When analysis was restricted to known metabolites, 53 differentially dysregulated metabolites distinguished WD from HC, with 48 metabolites retaining significance after FDR adjustment (Figure 1A and Table S2). Heat-maps visualizing fold change in intensity and metabolite significance when comparing WD to HC are displayed in Figure 2A,C.

### 2.3. Identification of Serum Metabolites Distinguishing between WD Phenotypes

Volcano analysis, corrected for multiple testing, comparing WD subjects stratified by clinical presentation to HC showed a total of 41, 51, and 52 metabolites distinguishing WDP, WDN, and WDH, respectively (Figure 1B–D). Twenty-seven metabolites were differentially altered in all WD phenotypes. A Venn diagram of common and unique metabolites between WD subgroups is shown in Figure 1E. Significant metabolites selected by volcano plot analysis are listed in Tables S3–S5 for WDP, WDN, and WDH, respectively. Heat-maps visualizing fold changes and metabolite significance when comparing different WD phenotypes to HC are displayed in Figure 2B,D.

ANOVA was performed to investigate if a metabolomic phenotype can distinguish between WD presentations. Post hoc analysis showed 11 metabolites significantly different among WD phenotypes; however, only 2 retained significance after FDR adjustment suggesting no significant difference between WD subgroups.

### 2.4. Choline Is a Hallmark Alteration in Patients with WD

In subjects with WD, choline was the most significantly altered metabolite with a 4-fold increase. When stratified to WD phenotypes, a significant linear increase in choline was observed (WDP < WDN < WDH) versus HC. Moreover, betaine, a derivative of choline, was significantly elevated when comparing WDH to HC (Figure 2B and Table S6).

There was a non-significant increase in acetylcholine observed between WD and HC. When stratifying to WD phenotypes, an increasing trend was observed (WDP < WDN < WDH); however, this was significant only in WDH compared to HC (Figure 2B and Table S6). Choline correlated positively with betaine and acetylcholine as well as many PC species containing saturated fatty acyl chains (FACs). Conversely, it was negatively correlated with S1P and lysophosphatidylcholines (LysoPCs) primarily with saturated FACs (Table S6).

### 2.5. Patients with WD Present Elevation in Some Amino Acid Levels

Compared to HC, phenylalanine, tyrosine, methionine, ornithine, proline, histidine, and alanine were more abundant in subjects with WD (Figure 2C and Table S2). Phenylalanine levels were significantly increased along with its downstream metabolite, tyrosine. The elevation in phenylalanine was significant across all WD phenotypes, while tyrosine was significantly elevated in symptomatic subjects. (Figure 2D and Tables S3–S5). Metabolites correlated with phenylalanine are shown in Table S7.

Compared to HC, methionine was increased 2.5-fold in WD subjects, with a significant increasing trend when comparing WD phenotypes to HC (WDP < WDN < WDH). (Figure 2C and Tables S3–S5). Metabolites correlated with methionine are shown in Table S8.

Ornithine, proline, histidine, and alanine also increased significantly in WD compared to HC, with a significant elevation observed across all WD subgroups (Figure 2C and Tables S3–S5).

### 2.6. Altered Circulating Phospholipids in WD

Many circulating PL species are altered in subjects with WD. Compared to HC, WD was characterized by an increase in PCs mainly containing saturated FACs and a decrease in many PCs containing polyunsaturated FACs (Figure 2A and Table S4). We also observed a reduction in S1P across all WD subgroups as well as altered levels in many SM species (Figure 2B and Tables S5–S7).

### 2.7. Secondary Bile Acids Are Increased in WD

The secondary bile acids glycocholic acid and glycoursodeoxycholic acid levels were significantly increased more than 3-fold in WD compared to HC (Figure 2C and Table S2). This increase was driven solely by elevations in symptomatic subgroups, WDN and WDH (Figure 2D and Tables S4 and S5). Although not significant, deoxycholic acid was increased and cholesterol levels were decreased in WD compared to HC (Figure 2C and Table S2).

### 2.8. Decreased Serum Levels of Long-Chain Acylcarnitines in WD

Decreased acylcarnitine (CAR) levels were observed in WD subjects compared to HC, with significantly lower levels of decanoyl-L-carnitine (CAR10:0), lauroyl L-carnitine (CAR12:0), and palmitoyl-L-carnitine (CAR16:0) (Figure 2C and Table S2). Carnitine was significantly elevated in WDP and WDN compared to HC (Figure 2D and Tables S3 and S4).

### 2.9. No Sex-Specific Differences between WD and HC

No significantly different levels of metabolites were found after FDR correction, suggesting no sex-specific effect in WD patients.

### 2.10. Gene Expression Analysis in tx-j Mice and Response to Copper Chelation

**Figure 3:**
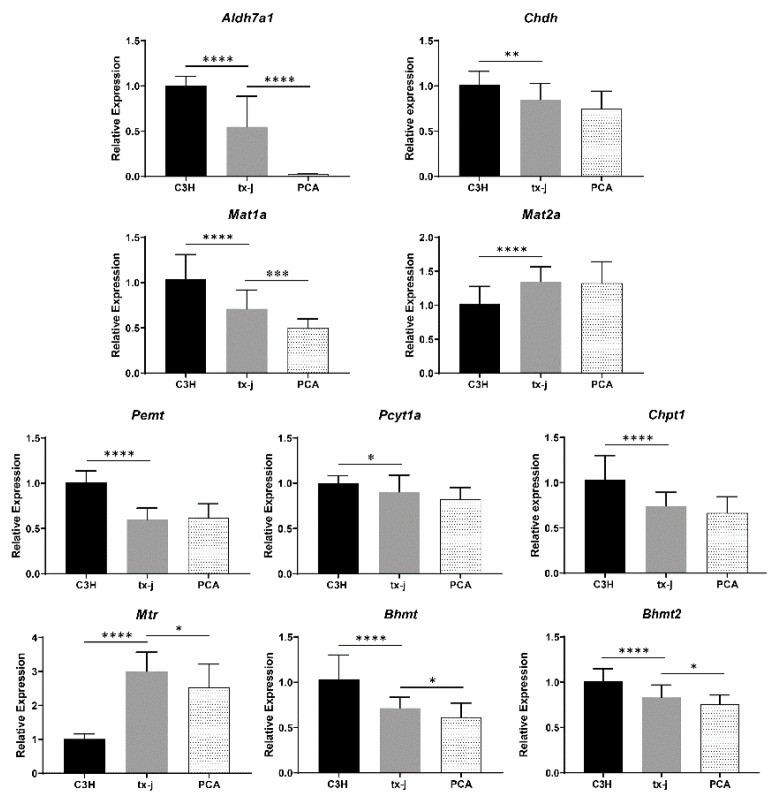
Analysis of genes related to choline and methionine in tx-j mice. C3H, control mice (*n* = 22); tx-j, Wilson disease mouse model (*n* = 22); PCA, tx-j mice treated with penicillamine (*n* = 22). *Aldh7a1*, aldehyde dehydrogenase family 7, member A1; *Chdh*, choline dehydrogenase; *Pemt*, phosphatidylethanolamine methyltransferase; *Pcyt1a*, phosphate cytidylyltransferase 1, choline, alpha isoform; *Chpt1*, choline phosphotransferase 1; *Mtr*, methionine synthase; *Bhmt* and *Bhmt2*, betaine-homocysteine methyltransferase and methyltransferase 2; *Mat1a* and *Mat2a*, methionine adenosyltransferase I and II, alpha. All genes are normalized to *Gapdh*. Data are presented as mean ± SD. Significance analyzed by Student’s t-test. * *p* < 0.05; ** *p* < 0.01; *** *p* < 0.001; **** *p* < 0.0001.

To validate the above findings, we checked the expression of genes related to choline and methionine metabolism in the Jackson Laboratory toxic milk model of WD, C3He-Atp7b^tx-J^/J (tx-j), compared to wild-type C3HeB/FeJ (C3H). In tx-j mice, transcript levels of aldehyde dehydrogenase family 7, member A1 (*Aldh7a1*) and choline dehydrogenase (*Chdh*), genes that convert choline to betaine, and transcript levels of *Pemt*, [phosphate cytidylyltransferase 1, choline, alpha isoform] (*Pcyt1a*), and choline phosphotransferase 1 (*Chpt1*), genes related to PC synthesis, were all significantly down-regulated compared to C3H control and were not corrected by PCA treatment (Figure 3).

Methionine synthase (*Mtr*) transcript levels were significantly up-regulated in tx-j mice and PCA treatment induced a mild but significant reduction. *Bhmt* and *Bhmt2* were significantly down-regulated in tx-j mice compared to C3H control and PCA treatment resulted in a further mild but significant reduction. The transcript level of methionine adenosyltransferase I, alpha (*Mat1a*) was significantly down-regulated in tx-j mice with further significant reduction in response to PCA. Conversely, methionine adenosyltransferase II, alpha (*Mat2a*) was significantly increased compared to C3H control with no difference between tx-j and PCA groups.

### 2.11. Pathway Analysis

**Figure 4:**
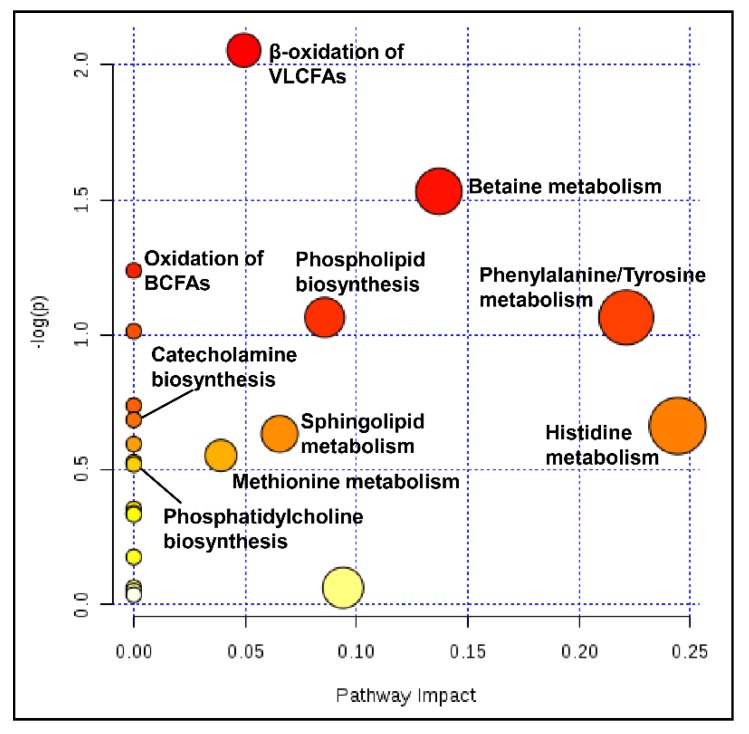
Metabolic pathway analysis. Node plot showing matched pathways according to significance (*p*-value) as determined by pathway enrichment analysis (*y*-axis), and pathways impact as determined by topology analysis (*x*-axis). Nodes in red indicate significance (*p* < 0.05), and the size of the nodes indicate impact. VLCFA, very long chain fatty acid; BCFA, branched chain fatty acid.

Results show that pathways with significantly altered metabolites and highest impact are involved in the metabolism of phenylalanine, tyrosine, histidine, and betaine (Figure 4 and Table S9). This is followed by pathways related to the biosynthesis of PLs, SLs, and methionine metabolism. Other pathways highlighted with significance, yet less impact, include oxidation of fatty acids, catecholamine synthesis, and phosphatidylcholine metabolism.

## 3. Discussion

WD is characterized by genetic, epigenetic, and metabolic changes. Our major findings are interconnected: (1) evidence of dysregulated choline and downstream PL metabolism in WD; (2) remarkable dysregulation in methionine and aromatic amino acids; and (3) signs of dysregulated urea, bile metabolism, and fatty acid oxidation in subjects with WD.

Our results show a serum choline increase of 4-fold in all WD phenotypes, suggesting elevated choline is a feature of WD, independent of diet and liver disease severity. The main metabolic fate of choline is PL synthesis, including PCs and SLs [14]. In our study, circulating PC and SL levels were differentially altered in WD compared to HC, indicating dysregulated PL metabolism. PC can be synthesized via the PEMT pathway and CDP-choline pathway. The PEMT pathway contributes 30% of the PC pool and is the source of de novo choline in mammals [36]. Results from tx-j mouse hepatic gene expression reveal significant reduction in *Pemt* transcript levels that were not corrected by copper chelation therapy. This indicates an impaired synthesis of PC and de novo choline via the PEMT pathway and possible involvement of mechanisms other than copper toxicity in regulating PEMT.

The majority of PC is synthesized by the CDP-choline pathway, a major consumer of choline [36], and PC profiles from CDP-choline and PEMT pathways are distinctive. CDP-choline produces mainly PCs with medium chain, saturated and monounsaturated FACs, whereas the liver-specific PEMT produces PCs containing longer, polyunsaturated FACs [36]. In our study, we observed a reduction in several PCs containing polyunsaturated FACs and an increase in many PCs with saturated FACs; choline was found to be positively correlated with the latter, implying an increase in these species is not met with choline clearance. Therefore, it seems likely the CDP-choline pathway is impaired. In support of this, hepatic transcript analyses from tx-j mice indicates down-regulation of the CDP-choline pathway independent of copper overload, as evident by a significant down-regulation of *Pcyt1a* and *Chpt1* that was not restored by PCA therapy.

We observed decreased levels of several circulating LysoPCs containing saturated FACs across all WD phenotypes. LysoPCs are generated by phospholipases A1 and A2 action on membrane-bound PCs, or lecithin-cholesterol acyltransferase action on lipoprotein-bound PCs [37,38]. Once formed, LysoPCs can be degraded or promptly transported to target tissue to activate signaling pathways involved in oxidative stress and inflammation [39,40,41,42]. The inflammation-mediating role of LysoPCs are thought to be a function of their FACs; the anti-inflammatory unsaturated LysoPCs offset the pro-inflammatory effect of saturated LysoPCs [43,44,45]. We and others have previously shown WD is an inflammatory state with elevated serum inflammation mediators [9,39,46]. The observed decreased level in multiple LysoPC species with saturated FACs may contradict an inflammatory role; however, we cannot conclude on the fate of these species as the rates of synthesis and clearance ultimately determine circulating levels. Of note, decreased LysoPC levels were also reported in other inflammatory states, including obesity and Alzheimer’s disease [47,48,49,50,51].

Altered PL levels may have implications for hepatic steatosis. PLs have a critical role in VLDL secretion and hepatic triglyceride export, and compelling evidence suggests hepatic PC synthesis via CDP-choline and PEMT pathways is independently essential for VLDL secretion [12,21]. Moreover, PC is a regulator of lipogenesis, as seen in mice and human HepG2 hepatoma cells; blocking PC synthesis up-regulates sterol regulatory element-binding protein 1 transcription and results in steatosis [52]. Additionally, mitochondrial lipidomic composition is critical for its membrane characteristics and bioenergetics [12]. Mitochondrial dysfunction and morphological changes are described in WD [6,7].

SMs are formed from PC and ceramide [22]. Our results reveal several altered SM levels mainly impacting symptomatic WD subjects; coincident with this, there was a significant decrease in S1P levels, evident in all WD phenotypes. This indicates dysregulated SL metabolism, a state reported in neurologic conditions and in nonalcoholic fatty liver [53,54,55,56,57,58,59]. S1P is formed from sphingosines via sphingosine kinases 1 and 2 (SPHK1 and SPHK2) activities and is interconvertible with sphingosine and ceramide [23]. These bioactive lipids effect opposing signals and through their interconversion, known as “the sphingolipid rheostat,” the cell’s fate is determined. Sphingosine and ceramide induce apoptosis, while S1P suppresses ceramide-mediated apoptosis and promotes proliferation and cell survival [24,25,60]. An up-regulated apoptosis contributes to hepatic pathologies, including fibrosis and inflammation, and some neurodegenerative disorders [61,62]. In subjects with WD, copper-induced apoptosis is mediated by suppression of survival and induction of caspase-3, TNFα, IL8, NF_K_B [63,64]. Of note, the activity of SPHK1 is shown to be inhibited by oxidative stress [65,66]. Therefore, it would be reasonable to postulate the observed reduction in S1P in WD patients may be due to copper-induced inhibition of SPHK1 by ROS, and this reduction may be involved in apoptotic mechanisms associated with WD.

We report a significant elevation in betaine in WD subjects, mainly driven by the WDH phenotype and suggesting a role for hepatic function. Betaine is the oxidized form of choline and is the methyl donor in methionine regeneration via BHMT [67]. The elevated betaine levels could be explained by the increased availability of choline. In addition, oxidative stress inhibits BHMT and results in elevated betaine [68]. In the livers of tx-j mice, decreased transcript levels of *Chdh* and *Aldh7a1* indicate down-regulation of choline’s conversion to betaine; therefore, the elevation in betaine may not be due to the increased availability of its substrate. However, tx-j mice also show a down-regulation in *Bhmt* hepatic transcript levels, possibly indicating indirect BHMT inhibition through decreased gene expression, thereby reducing betaine input toward methionine regeneration with consequent betaine accumulation.

Methionine is an essential amino acid, metabolized in the liver to form SAM via methionine adenosyltransferases (MAT) [69,70]. MAT is encoded by two genes, the liver-predominant *MAT1A* that encodes MATI/III and *MAT2A*, which is expressed less in the liver and more in extrahepatic tissues and encodes for MATII [71,72]. We and others have reported dysregulated methionine in animal models of WD [9,10] and, to our knowledge, hypermethioninemia has not been described previously in WD subjects. In our current results, methionine levels were increased by more than 2-fold in WD subjects compared to HC, and this elevation was significantly higher in WDH, suggesting liver pathology may have a role in our findings. The mechanisms leading to methionine elevation are varied and include MAT deficiency [73]. ROS inhibits MAT I/III, which are encoded by *MAT1A* [10,74]. In tx-j mice, we found hepatic *Mat1a* expression was significantly reduced and *Mat2a* was increased. This corroborates findings from other animal models of WD in which a reduction in *Mat1a* gene expression was compensated by an increase in *Mat2a*; however, this increase was not enough to sustain the total enzymatic function, as MATII has the lowest specific activity [10,75]. MAT III has the highest K_m_ for methionine (215 μM–7 mM); MAT I has an intermediate K_m_ (23 μM–1 mM); and MAT II has the lowest K_m_ (∼4–10 μM) [76,77].

Alternatively, elevated methionine levels can result from high homocysteine, which can induce hepatotoxic and neurotoxic effects in WD [78,79,80]. However, the involvement of homocysteine is uncertain, given we did not measure homocysteine, and hyperhomocysteinemia has not been described previously in WD. Homocysteine can regenerate methionine via MTR or BHMT with betaine as a methyl donor [81,82]. It was shown that a reduction in SAM deactivates cystathionine β-synthase, the first step in transsulfuration and a primary homocysteine clearance route [83,84]. Elevated copper inhibits S-adenosylhomocysteine hydrolase (SAHH) activity and expression [10,85,86,87]. Taken together, we propose these actions divert the homocysteine pool away from transsulfuration to re-methylation pathways, thereby contributing to the observed elevation in methionine. In our analysis, methionine correlated positively with betaine and choline (*r* = 0.8, *p* = 8.11 × 10^−20^ and *r* = 0.4, *p* = 2.39 × 10^−5^, respectively) suggesting an impaired BHMT pathway. Our findings from the tx-j mice substantiate this; hepatic *Mtr* transcript levels were up-regulated in untreated tx-j then showed a significant decrease in response to PCA, whereas *Bhmt* was down-regulated. This indicates homocysteine undergoes re-methylation to methionine via the folate-dependent pathway and is contributing to the elevated methionine pool in a copper-overload state.

Acetylcholine is synthesized from choline in cholinergic neurons and acts in a receptor-mediated fashion to regulate social behavior and cognitive function [28,88]. Our results show a non-significant elevation in acetylcholine in subjects with WD. When stratified by clinical presentation, a significant increase was observed in WDH patients. This rule out acetylcholine deficiency and indicates possible involvement of other mechanisms, including receptor function and signal termination, with regard to the development of neurologic symptoms in WD.

Acetylcholine is also relevant to hepatic manifestations as evidenced from animal and human stellate cells, which have shown cholinergic transmission to mediate hepatic stellate cell activation and fibrogenesis [89,90]. In contrast, an animal model of non-alcoholic steatohepatitis (NASH) points to an anti-inflammatory role exerted by cholinergic signaling in Kupffer cells, resulting in the suppression of NASH progression [91]. Further work is warranted to understand the role of acetylcholine and signaling pathways in hepatic WD pathogenesis.

We also found elevated phenylalanine and tyrosine levels in subjects with WD. Phenylalanine is a precursor for tyrosine and biogenic amine neurotransmitters dopamine, norepinephrine, and epinephrine, also known as catecholamines [92]. Defects in the catabolic pathways of phenylalanine and tyrosine deplete downstream neurotransmitters [93]. Abnormally elevated levels of these amino acids are reported with genetic enzymatic defects and are associated with neurologic and psychiatric symptoms [93,94,95,96].

Histidine levels were found to be significantly elevated in subjects with WD. Histidine is an essential amino acid and precursor for the biogenic amine neurotransmitter histamine, formed via oxidative decarboxylation by histidine-decarboxylase [97]. This is a catabolic pathway that overlaps with folate metabolism; histidine is converted to glutamic acid via multiple reactions involving folate-dependent enzymes [98]. Dysregulated biogenic amines neurotransmission is reported in WD with neuropsychiatric manifestations [99,100,101,102,103,104,105,106], and dysfunctions of the histaminergic nervous system are involved in many neurologic disorders [107,108,109]. Our findings indicate a possible defect in phenylalanine, tyrosine, and histidine catabolic pathways resulting in accumulation of these amino acids in WD, regardless of clinical presentation. They also suggest development of neurologic manifestations may be indirect and involve mechanisms other than simply neurotransmitter depletion.

Proline and ornithine were increased in subjects with WD. Proline is synthesized from ornithine and glutamic acid in reversible reactions through an intermediate mitochondrial metabolite [110]. Ornithine is also formed as a part of the urea cycle [111]. Although non-significant, reduced urea was observed in WD subjects. Elevated proline may be due to an increased availability of its precursors, ornithine and glutamic acid. We recently reported an elevation in glutamic acid and a decrease in urea levels in subjects with WD [35]. This, along with our current observation of reduced urea, suggests a dysregulated urea cycle in subjects with WD.

A non-significant reduction in serum cholesterol in WD subjects was also observed. A reduction in hepatic and circulating cholesterol levels is well-documented in animals and subjects with WD [34,112]. Bile is composed of bile acids, fatty acids, cholesterol, bilirubin, and PLs [113]. Secondary bile acids are produced by the action of microbiota on primary bile acids in the colonic environment [114]. Our results show a significant increase in secondary bile acids glycocholic acid and glycoursodeoxycholic acid, specific to symptomatic subjects. Collectively, the observed changes in cholesterol, PLs, and bile acids indicate a likely alteration in WD bile metabolism.

In our study, carnitine level was increased and there was a significant reduction in acylcarnitines CAR(10:0), CAR(12:0), and CAR(16:0). Long- and medium-chain CARs are the transport form of fatty acids, formed from acyl-CoAs and carnitine via carnitine palmitoyltransferase I in mitochondria and peroxisomes [115,116]. The circulating levels of long- and medium-chain CARs are thought to reflect tissue metabolic state and an increased level is proposed as a marker of metabolic dysfunction [117,118,119,120]. Elevated long- and medium-chain CARs are also observed in the fasted state, as fatty acid oxidation is at a peak [121,122]. The observed decreased circulating level of these CAR species and elevated carnitine in WD subjects indicate altered fatty acid oxidation and is an interesting observation worth further investigation.

Results from pathway analysis corroborate our metabolomic findings. Alterations in metabolite levels operating at the initiation of or junctions within pathways explain a high impact value. The observed elevation in phenylalanine and tyrosine signifies a dysregulated catabolic pathway of these amino acids with a cascading effect on the synthesis of catecholamine neurotransmitters epinephrine, norepinephrine, and dopamine. The overlapping choline and methionine metabolisms are dysregulated, with a down-stream effect on the biosynthesis of PLs, SLs, and bile. Although alteration in the latter was non-significant, this may be due to the limited bile acids detected with our metabolomic platform, and the observed alterations in PL levels and reduction in cholesterol highlight the need for further investigation. Moreover, alterations in carnitine and acylcarnitine levels affected oxidation pathways of fatty acids. Histidine metabolism was significantly altered, mainly driven by the elevated histidine. Although with lesser significance and impact value, our pathway analysis also highlighted a dysregulated ammonia recycling pathway. The elevated ornithine and non-significant urea reduction we found in WD subjects, along with our previous finding of elevated glutamic acid, highlights dysregulation in urea cycling and ammonia recycling that is worth continued investigation.

Our findings on the dysregulation in methionine, choline, aromatic amino acid, and urea metabolisms are interconnected. In copper overload, methionine metabolism is dysregulated, partly due to mechanisms involving ROS generation. Elevated copper, via ROS induction, inhibits SAHH activity and expression [10,85,86,87]. ROS also inhibit MAT I/III which are encoded by MAT1A [10,74]. This reaction is essential for the biosynthesis of the universal methyl donor SAM. Therefore, copper overload results in dysregulated methionine cycle and reduced methylation potential [9,10,11]. Choline metabolism is connected to the methionine cycle, as the biosynthesis of SAM and methylation reaction are essential for de novo choline synthesis [12,36]. In addition, choline contributes to the regeneration of methionine from homocysteine via the choline-dependent BHMT reaction [8].

**Figure 5:**
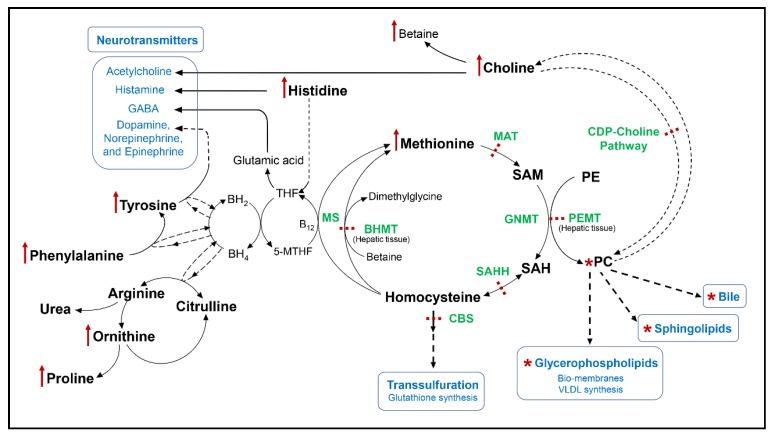
Schematic summary of targeted metabolomics results. Potential enzymatic defects in Wilson disease are depicted via interactions between methionine, choline, aromatic amino acid, and urea metabolisms. A red “up” arrow indicates differential increase; a red “*” indicates the class of metabolites includes differential increase for some and decrease for others; and a red dashed line indicates a potential enzymatic block. BH2, dihydrobiopterin; BH4, tetrahydrobiopterin; BHMT, betaine-homocysteine methyltransferase; CBS, cystathionine β-synthase; CDP-choline, cytidine-diphosphate-choline; GABA, gamma aminobutyric acid; Mat, methionine-adenosyl transferase; MS, methionine synthase; PC, phosphatidylcholine; PE, phosphatidylethanolamine; PEMT, phosphatidylethanolamine *N*-methyltransferase; SAHH, S-adenosylhomocysteine hydrolase; SAM, S-adenosylmethionine; THF, tetrahydrofolate; VLDL, very low density lipoprotein; 5-MTHF, 5-methyltetrahydrofolate.

Aromatic amino acid metabolism and the methionine cycle share folate as a cofactor. In one-carbon metabolism, homocysteine regenerates methionine via two routes; one is choline-dependent and the other is folate- and B12-dependent. Aromatic amino acid metabolism requires another cofactor, biopterin, that is also dependent upon the folate cycle for its recycling. An impaired folate cycle is suspected in WD, as reports of mutations in methylenetetrahydrofolate reductase are associated with early onset and hepatic phenotype [78]. However, the extent of folate cycle dysregulation is not clear and worth further investigation. Biopterin is also an essential cofactor for the conversion of arginine to citrulline and the synthesis of nitric oxide [123]. Both arginine and citrulline are intermediates in the urea cycle [124]. An illustration of our metabolomic results and interconnection of methionine, choline, aromatic amino acid, and urea metabolisms is shown in Figure 5.

## 4. Methods

### 4.1. Subject Recruitment and Features

Detailed subject recruitment and characteristics were previously described as part of the untargeted metabolomics study of the same cohort [35]. Briefly, serum samples of 76 subjects were obtained from the Institute of Neurology and Psychiatry in Warsaw, Poland. A total of 15 HC and 61 subjects diagnosed with WD according to Leipzig’s criteria [125] were studied. One subject was excluded due to incomplete information. Table S10 shows subject characteristics by clinical presentation. WD subjects were categorized according to presentation as prevalent neurologic (WDN, *n* = 22) or hepatic (WDH, *n* = 26). A subgroup of asymptomatic subjects was diagnosed based on family history and categorized as pre-clinical (WDP, *n* = 12). All patients were untreated as samples were obtained at the time of diagnosis. The study protocol was approved by the Institutional Review Board at the University of California, Davis, with IRB# 818454-13, on 27 December 2018.

### 4.2. Metabolomic Analysis

Hydrophilic interaction chromatography-quadrupole time-of-flight mass spectrometry (HILIC-QTOF MS) was used for polar phase lipid extraction to profile serum metabolites including choline, betaine, acetylcholine, acylcarnitines, PLs, SMs, and the amino acids phenylalanine, tyrosine, and histidine [126]. Data were collected on positive and negative ion mode; metabolite identification and annotation were done by matching retention time and mass-to-charge ratio to in-silico and in-house spectral libraries using MS-DIAL software [127]. Internal standards were added for quality control; details are listed in Table S13. Data were reported as normalized relative intensities. The metabolomics and metadata reported in this paper are available via Metabolomics Workbench; (http://www.metabolomicsworkbench.org) and study can be found under ST001259.

### 4.3. Mouse Models and Diets

Mouse experiments were carried out in the wild-type C3HeB/FeJ (C3H) and Jackson Laboratory toxic milk model of WD, C3He-Atp7b^tx-J^/J (tx-j). Colonies were maintained at 20–23 °C, 45–65% relative humidity, and a light cycle of 14 h light/10 h dark. C3H mice were maintained on LabDiet chow (Purina Mills, Inc., St. Louis, MO, USA; catalog #5001) and used as a control group, whereas tx-j mice received purified AIN-76A diet (Dyets Inc., Bethlehem, PA, USA; catalog #D110098). A subgroup of tx-j mice was treated with the copper chelator d-penicillamine (Sigma Inc., St. Louis, MO, USA; catalog #P4575; PCA, 100 mg/kg body *weight*/*day*) beginning at 12 weeks of age with oral administration through drinking water. For each group, *n* = 22 mice total–C3H control = 10 male/12 female, tx-j = 11 male/11 female, and tx-j treated with PCA = 12 male/10 female. As tx-j mice lack adequate copper concentration in breast milk for neonatal growth and development beyond day 10 on average, all tx-j pups were fostered to a lactating C3H dam by day 7 post-partum. At approximately 3 weeks of age, progeny were weaned and randomly divided into 2 treatment groups: tx-j (untreated) and PCA. At 24 weeks of age, mice were euthanized and livers were obtained for mRNA expression analysis.

All mouse protocols followed the guidelines of the American Association for Accreditation of Laboratory Animal Care and were reviewed and approved annually by the UC Davis Institutional Animal Care and Use Committee. All animals received humane care according to the criteria outlined in the “Guide for the Care and Use of Laboratory Animals” prepared by the National Academy of Sciences and published by the National Institutes of Health (NIH publication 86–23 revised 1985).

### 4.4. RNA Isolation and qPCR

Total RNA from frozen liver was isolated using the AllPrep DNA/RNA Mini Kit (QIAGEN, Germantown, MD, USA; catalog #80204). The concentration and purity of samples was determined by measuring the absorbency at A230, A260, and A280. Further, RNA integrity was checked by agarose gel electrophoresis. Total RNA was stored at −80 °C prior to downstream analysis. cDNA was synthesized using SuperScript III First-Strand cDNA Synthesis Kit (Invitrogen, catalog #18080051) according to the manufacturer’s protocol. Gene-specific primers were designed by Primer3 software and blasted against the mouse genome using NCBI blastn to check primer specificity (http://blast.ncbi.nlm.nih.gov/Blast.cgi). Primers were synthesized by (Eurofins Genomics, Louisville, KY, USA); sequences are listed in Table S12. Primer efficiency (E) was calculated from the slope of a standard curve generated via 10-fold serial dilution of pooled control cDNA using the formula (E = 10^(−1/slope)^-1). Next, cDNA was used as a template for amplification in qPCR (ViiA7 Real-Time PCR System; Applied Biosystems by Thermo Fisher Scientific, Carlsbad, CA, USA) using SYBR Green Master Mix (Applied Biosystems by Thermo Fisher Scientific, Carlsbad, CA, USA; catalog #4309155); all samples were plated in triplicate. Reaction conditions were programmed with initial denaturation at 50 °C for 2 min and 95 °C for 10 min followed by 40 cycles of 95 °C for 15 s and 60 °C for 1 min. Relative gene expression was calculated using 2^−ΔΔCT^ values and normalized to *Gapdh*.

### 4.5. Statistical Analysis

Statistical analysis was performed using MetaboAnalyst 4.0 (McGill University, Quebec, CA; http://metaboanalyst.ca) [128] and JMP (SAS Institute Inc., Cary, NC, USA; http://www.jmp.com). A total of 106 (1.2%) missing values were detected and replaced by the half of the minimum value for a feature, under the assumption that missing values are due to low abundance. Features with more than 20% of missing values were excluded from analysis. Data were normalized by the sum of the knowns, auto-scaled and transformed to the generalized log (glog 2). Features view showing distribution before and after normalization are shown in Figure S15. Principal component analysis was used to asses for outliers and unsupervised clustering. Volcano analysis was performed to compare differential metabolites between HC and WD and to compare the fold change and significance. Fold-change threshold was set to >1.2 and outcome *p*-value was adjusted for FDR using the Benjamini–Hochberg method, with *p* < 0.1 considered significant [129]. To identify differential metabolites within the WD subgroups, we performed volcano analyses comparing HC to WDP, WDN, and WDH separately, and heat-maps were generated to visualize intensities based on FDR-adjusted significant metabolites between HC and different WD phenotypes. Pearson’s correlation analysis was performed against metabolites of interest to determine associated metabolites, significance indicated with *p* < 0.05. Student’s t-test was performed to analyze gene transcript levels in tx-j mice compared to C3H control, significance indicated with *p* < 0.05.

### 4.6. Metabolite Enrichment and Pathway Analysis

To identify pathways impacted by the state of WD, The Pathway Analysis module in MetaboAnalyst 4.0 was employed to combine metabolite enrichment with pathway topology [130]. Metabolites with FDR-adjusted *p* < 0.1 were used as an input, compared against pathway-associated sets of metabolites from The Small Molecule Pathway Database. In metabolite enrichment, over-representation analysis was used to determine pathway significance. Hypergeometric, one-tailed *p*-values are reported after adjusting for multiple testing, and *p* < 0.05 was considered significant. In topology analysis, the positional importance of metabolites within a pathway is determined. The relative betweenness method was used and an impact score ranging between 0 and 1 was reported [131]. Results are represented as a node plot and a table of pathways with altered metabolite sets, impact scores, and associated *p*-values.

## 5. Conclusions

The present study employed metabolomic and univariate approaches to identify metabolites distinguishing subjects with WD from HC. Our results reveal WD is a state of dysregulated choline metabolism, potentially impacting downstream PL and bile biosynthesis and possibly playing a key role in the development of hepatic pathology associated with WD. Dysregulated choline metabolism may also alter acetylcholine biosynthesis. We also observed signs of impaired aromatic amino acid degradation pathways that could impact neurotransmitter synthesis. Together, these may present implications for the development of neurologic manifestations in WD. We also provide further insight into points of a dysregulated methionine cycle due to copper overload or related liver complications, evident by the state of hypermethioninemia, and possible impairment of BHMT and MAT reactions. The consequences of observed metabolite alterations with specific regard to the development of hepatic and neurologic manifestations require deeper examination. Findings from gene expression in tx-j mice suggest impaired PC synthesis, evident by the down-regulation of enzymatic genes in the CDP-choline and PEMT pathways. They also highlight the role of folate-dependent homocysteine re-methylation as the main contributor to the methionine pool with up-regulated transcript levels of *Mtr* and down-regulated levels of choline-dependent *Bhmt*.

Our findings contribute to the understanding of metabolic dysregulations associated with copper overload and highlight the possible involvement of choline and aromatic amino acids as well as folate and biopterin in the development of WD phenotypes. Further work is warranted to elucidate related mechanisms and to identify potential therapeutic targets aimed at restoring described altered metabolic pathways. One limitation of this study is that liver histology was not available to confirm the state of liver disease in WD subjects. This could possibly explain the partial overlap in metabolomic profiles observed between different phenotypes. Follow-up studies supported with biopsy-characterized liver disease state in WD would help elucidate effects of the observed metabolic perturbations on hepatic manifestations. The process of PL turnover and remodeling also needs further examination as a source of endogenous choline.

## Supplementary Materials

The supplementary materials are available online at https://www.mdpi.com/1422-0067/20/23/5937/s1.

## Abbreviations

*Aldh7a1*	Aldehyde dehydrogenase family 7, member A1
BHMT	Betaine-homocysteine S-methyltransferase
C3H	C3HeB/FeJ
CAR	Acylcarnitine
CDP-choline	Cytidine-diphosphate-choline
*Chdh*	Choline dehydrogenase
*Chpt1*	Cholinephosphotransferase 1
FAC	Fatty acyl chain
FDR	False discovery rate
HC	Healthy control
LysoPC	Lysophosphatidylcholine
*Mat1a*	Methionine adenosyltransferase I alpha
*Mat2a*	Methionine adenosyltransferase II, alpha
*Mtr*	Methionine synthase
NASH	Non-alcoholic steatohepatitis
PC	Phosphatidylcholine
PCA	Penicillamine
*Pcyt1a*	Phosphate cytidylyltransferase 1, choline, alpha isoform
PEMT	Phosphatidylethanolamine N-methyltransferase
PL	Phospholipid
PUFA	Polyunsaturated fatty acyl
ROS	Reactive oxygen species
S1P	Sphingosine-1-phosphate
SAH	S-adenosylhomocysteine
SAM	S-adenosylmethionine
SL	Sphingolipid
SM	Sphingomyelin
SPHK	Sphingosine kinase
tx-j	C3He-Atp7btx-J/J
VLDL	Very low-density lipoprotein
WD	Wilson disease
WDH	Wilson disease hepatic
WDN	Wilson disease neurologic
WDP	Wilson disease pre-clinical
