# Supplementary material for: Dysregulated Choline, Methionine, and Aromatic Amino Acid Metabolism in Patients with Wilson Disease: Exploratory Metabolomic Profiling and Implications for Hepatic and Neurologic Phenotypes
Source: Int J Mol Sci. 2019 Nov 26;20(23):5937. doi: 10.3390/ijms20235937 (PMC6928853; doi:10.3390/ijms20235937)

**Supplemental Material**

**Figure S1:** Principal component analysis based on all detected metabolites. The first and second principal component scores plot show separation and clustering of WD and HC groups with some overlapping. The explained variances are shown in brackets.


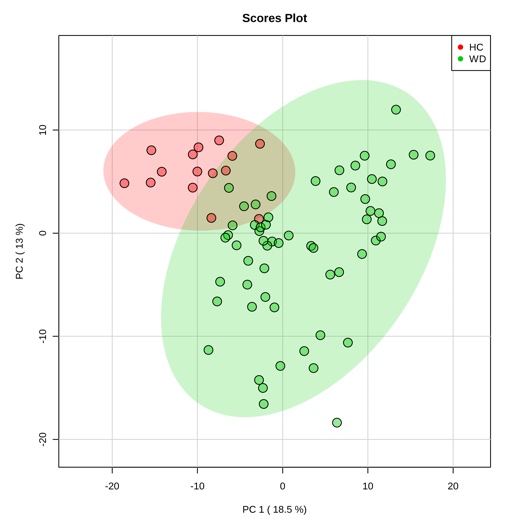


**Figure S2:** Volcano plot for WD compared to HC based on all detected metabolites. Important features selected with fold change (FC) threshold 1.2 and t-test (p) threshold 0.1. Both fold changes and *p*-values are log-transformed. The pink circles represent features above the threshold. The further the circle’s position away from (0,0), the more significant the feature.

**
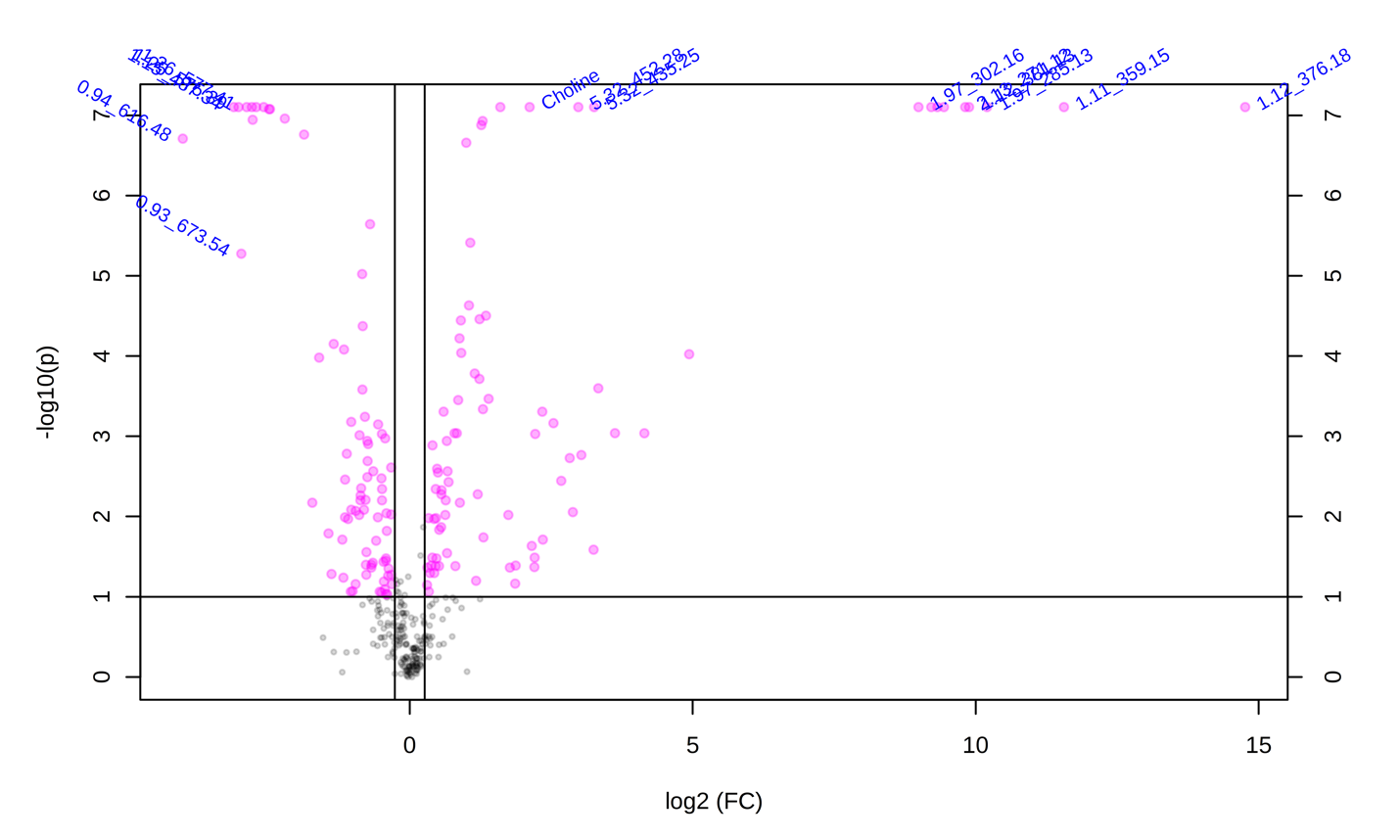
**

**Table S1:** Comprehensive list of differential metabolites comparing WD to HC. Significant metabolites were selected by volcano plot with fold change (FC) >1.2 and FDR-adjusted *p* <0.1 indicating significance.

| Metabolite Name/Identifier | InChI Key | FC | log2  (FC) | FDR  *p*-value | -log10  (p) | Species | m/z | RT |
| --- | --- | --- | --- | --- | --- | --- | --- | --- |
| 1.97_307.11 | NA | 594.48 | 9.2155 | 1.00E-73 | 72.998 | [M+H]+ | 307.1147 | 1.97 |
| 1.97_285.13 | NA | 1176.9 | 10.201 | 7.06E-64 | 63.151 | [M+H-H2O]+ | 285.1333 | 1.97 |
| 2.12_293.10 | NA | 640.54 | 9.3232 | 8.09E-64 | 63.092 | [2M+H]+ | 293.0991 | 2.12 |
| 1.11_359.15 | NA | 3018.3 | 11.56 | 2.28E-59 | 58.643 | [M+H]+ | 359.152 | 1.11 |
| 1.12_376.18 | NA | 27746 | 14.76 | 3.83E-58 | 57.417 | [M+H]+ | 376.1759 | 1.12 |
| 2.13_271.12 | NA | 900.89 | 9.8152 | 2.70E-57 | 56.569 | [2M+H]+ | 271.1173 | 2.13 |
| 2.13_288.14 | NA | 694.27 | 9.4394 | 8.44E-57 | 56.074 | [M+H]+ | 288.1439 | 2.13 |
| 1.97_302.16 | NA | 508.34 | 8.9897 | 8.00E-56 | 55.097 | [M+H]+ | 302.1595 | 1.97 |
| 1.12_381.13 | NA | 932.02 | 9.8642 | 2.78E-36 | 35.555 | [M+Na]+ | 381.1296 | 1.12 |
| 5.32_435.25 | NA | 9.5686 | 3.2583 | 2.56E-20 | 19.591 | [M-H]- | 435.2497 | 5.32 |
| 5.31_104.11 | NA | 3.0336 | 1.601 | 1.87E-17 | 16.729 | [M+H]+ | 104.1061 | 5.31 |
| 1.21_531.37 | NA | 0.15263 | -2.7119 | 5.34E-16 | 15.272 | [M+H]+ | 531.368 | 1.21 |
| Choline | OEYIOHPDSNJKLS-UHFFFAOYSA-N | 4.3424 | 2.1185 | 8.76E-16 | 15.058 | [M+H]+ | 104.1066 | 5.12 |
| 5.32_452.28 | NA | 7.8835 | 2.9788 | 9.66E-15 | 14.015 | [M-H]- | 452.2765 | 5.32 |
| 0.94_616.48 | NA | 0.06208 | -4.0097 | 7.96E-14 | 13.099 | [M+H]+ | 616.4769 | 0.94 |
| 1.26_577.41 | NA | 0.13579 | -2.8805 | 2.78E-13 | 12.557 | [M+H]+ | 577.4071 | 1.26 |
| 1.25_575.39 | NA | 0.12284 | -3.0252 | 4.11E-13 | 12.386 | [M+H]+ | 575.3942 | 1.25 |
| Phenylalanine | COLNVLDHVKWLRT-QMMMGPOBSA-N | 2.4421 | 1.2881 | 2.79E-12 | 11.554 | [M+H]+ | 166.0862 | 6.61 |
| 2-Hydroxy  carbamazepine | NA | 2.403 | 1.2649 | 3.19E-12 | 11.496 | [M+H]+ | 253.0974 | 1.24 |
| 1.16_489.36 | NA | 0.27424 | -1.8665 | 4.03E-12 | 11.395 | [M+H]+ | 489.3574 | 1.16 |
| 6.61_149.06 | NA | 1.9985 | 0.99891 | 4.96E-11 | 10.305 | [M-H]- | 149.0589 | 6.61 |
| 0.93_672.54 | NA | 0.14607 | -2.7752 | 6.42E-11 | 10.192 | [M+H]+ | 672.5381 | 0.93 |
| 1.23_533.38 | NA | 0.18064 | -2.4688 | 2.06E-10 | 9.6861 | [M+H]+ | 533.3839 | 1.23 |
| 0.93_673.54 | NA | 0.12712 | -2.9757 | 6.32E-10 | 9.1991 | [M+H]+ | 673.5387 | 0.93 |
| 1.15_487.34 | NA | 0.11591 | -3.109 | 8.29E-09 | 8.0815 | [M-H]- | 487.3427 | 1.15 |
| 1.26_555.40 | NA | 0.16726 | -2.5799 | 6.00E-08 | 7.222 | [M+NH4]+ | 555.3977 | 1.26 |
| 1.23_553.38 | NA | 0.14464 | -2.7895 | 1.52E-07 | 6.8196 | [M+H]+ | 553.3824 | 1.23 |
| 5.56_852.56 | NA | 2.0657 | 1.0467 | 2.47E-07 | 6.6072 | [M+H]+ | 852.5609 | 5.56 |
| PC(18:1/14:0) | KIVAJCJTVPWSRJ-OQHNRNOKSA-N | 1.8408 | 0.88035 | 1.07E-06 | 5.9716 | [M+H]+ | 732.5534 | 4.43 |
| 5.56_835.53 | NA | 1.8711 | 0.90387 | 1.07E-06 | 5.9716 | [M+H]+ | 835.5327 | 5.56 |
| 1.21_511.37 | NA | 0.21659 | -2.2069 | 1.24E-06 | 5.9057 | [M+H]+ | 511.3718 | 1.21 |
| 5.15_544.34 | NA | 0.5586 | -0.84012 | 1.43E-06 | 5.8433 | [M+Na]+ | 544.3397 | 5.15 |
| 1.45_738.49 | NA | 0.6154 | -0.7004 | 1.43E-06 | 5.8433 | [M+NH4]+ | 738.4926 | 1.45 |
| 0.99_394.35 | NA | 30.647 | 4.9377 | 1.43E-06 | 5.8433 | [M+NH4]+ | 394.3517 | 0.99 |
| 5.56_854.57 | NA | 2.5438 | 1.347 | 2.98E-06 | 5.5258 | [M+NH4]+ | 854.5748 | 5.56 |
| 5.56_837.55 | NA | 2.352 | 1.2339 | 5.34E-06 | 5.2725 | [M+H]+ | 837.5476 | 5.56 |
| Ornithine | AHLPHDHHMVZTML-BYPYZUCNSA-N | 2.3478 | 1.2313 | 9.17E-06 | 5.0379 | [M+H]+ | 133.0961 | 9.27 |
| PC(14:0/14:0) | CITHEXJVPOWHKC-UUWRZZSWSA-N | 2.0995 | 1.0701 | 1.13E-05 | 4.9477 | [M+H]+ | 678.5046 | 4.49 |
| 9.27_116.07 | NA | 2.2169 | 1.1485 | 3.22E-05 | 4.4922 | [M+H]+ | 116.0696 | 9.27 |
| LysoPC(P-18:0) | WBOMIOWRFSPZMC-AYICAFKVSA-N | 0.57314 | -0.80303 | 3.23E-05 | 4.4903 | [M+H]+ | 508.3748 | 5.22 |
| 1.18_509.36 | NA | 0.17857 | -2.4855 | 5.75E-05 | 4.2404 | [M+H]+ | 509.3562 | 1.18 |
| 3.07_400.34 | NA | 2.4515 | 1.2937 | 7.14E-05 | 4.1462 | [M+H]+ | 400.3414 | 3.07 |
| 0.99_377.32 | NA | 17.709 | 4.1464 | 7.20E-05 | 4.1424 | [M+H]+ | 377.3205 | 0.99 |
| 0.92_600.50 | NA | 0.60122 | -0.73405 | 8.27E-05 | 4.0824 | [M+H]+ | 600.4984 | 0.92 |
| 0.90_610.54 | NA | 1.5746 | 0.65502 | 8.51E-05 | 4.0699 | [M+H]+ | 610.5391 | 0.90 |
| Histidine | HNDVDQJCIGZPNO-YFKPBYRVSA-N | 1.7805 | 0.83231 | 9.31E-05 | 4.0313 | [M+H]+ | 156.0762 | 9.07 |
| Alanine | QNAYBMKLOCPYGJ-REOHCLBHSA-N | 1.3987 | 0.4841 | 0.00013065 | 3.8839 | [M+H]+ | 90.0542 | 7.79 |
| 1.08_541.45 | NA | 0.32984 | -1.6002 | 0.00014455 | 3.84 | [M+H]+ | 541.4482 | 1.08 |
| 0.93_364.27 | NA | 5.815 | 2.5398 | 0.00014455 | 3.84 | [M+H]+ | 364.2681 | 0.93 |
| 0.90_611.54 | NA | 1.5898 | 0.66887 | 0.00014666 | 3.8337 | [M+H]+ | 611.5373 | 0.90 |
| 2.87_426.36 | NA | 4.6498 | 2.2172 | 0.00018085 | 3.7427 | [M+H]+ | 426.3571 | 2.87 |
| 4.30_732.55 | NA | 1.8801 | 0.91079 | 0.00028574 | 3.544 | [M+H]+ | 732.5535 | 4.30 |
| 5.13_500.30 | NA | 8.1828 | 3.0326 | 0.00028574 | 3.544 | [M+H]+ | 500.303 | 5.13 |
| 1.00_551.55 | NA | 10.073 | 3.3324 | 0.00028574 | 3.544 | [M+H]+ | 551.5454 | 1.00 |
| PS(18:0/20:4) | SVOUGFFDROZBJI-DNALCEECSA-N | 0.39422 | -1.3429 | 0.00031406 | 3.503 | [M+H]+ | 812.5433 | 5.40 |
| LysoPC(15:0) | RJZVWDTYEWCUAR-JOCHJYFZSA-N | 0.30327 | -1.7213 | 0.00034956 | 3.4565 | [M+H]+ _[M+Na]+ | 482.3215 _504.3075 | 5.27 |
| 1.09_466.36 | NA | 0.4885 | -1.0336 | 0.00034956 | 3.4565 | [M+H]+ | 466.3605 | 1.09 |
| LysoPC(14:0) | VXUOFDJKYGDUJI-OAQYLSRUSA-N | 0.57791 | -0.79108 | 0.00034956 | 3.4565 | [M+H]+ | 468.3086 | 5.29 |
| S1P | DUYSYHSSBDVJSM-KRWOKUGFSA-N | 0.67908 | -0.55836 | 0.00034956 | 3.4565 | [M+H]+ | 380.2552 | 5.96 |
| Kynurenine | YGPSJZOEDVAXAB-QMMMGPOBSA-N | 1.5142 | 0.59854 | 0.00050566 | 3.2961 | [M+H]+ | 209.0914 | 6.62 |
| 5.16_482.32 | NA | 0.44705 | -1.1615 | 0.00075461 | 3.1223 | [M+H]+ | 482.3218 | 5.16 |
| LysoPC(24:0) | SKJMUADLQLZAGH-WJOKGBTCSA-N | 0.74014 | -0.43413 | 0.0010653 | 2.9725 | [M+H]+ | 608.4634 | 5.03 |
| 1.00_549.53 | NA | 7.3728 | 2.8822 | 0.0011621 | 2.9348 | [2M+H]+ | 549.5341 | 1.00 |
| Tyrosine | OUYCCCASQSFEME-QMMMGPOBSA-N | 1.7307 | 0.79133 | 0.0012417 | 2.906 | [M+H]+ | 182.0802 | 7.39 |
| 1.01_507.49 | NA | 7.0977 | 2.8274 | 0.0012417 | 2.906 | [2M+H]+ | 507.4856 | 1.01 |
| 0.99_591.58 | NA | 0.55183 | -0.85771 | 0.0013086 | 2.8832 | [M+H]+ | 591.5819 | 0.99 |
| PC(18:3/18:3) | XXKFQTJOJZELMD-JICBSJGISA-N | 0.59446 | -0.75035 | 0.0014346 | 2.8433 | [M+H]+ | 778.5374 | 4.18 |
| 1.14_481.35 | NA | 0.45316 | -1.1419 | 0.0019149 | 2.7178 | [M+H]+ | 481.3514 | 1.14 |
| PC(P-18:0/20:4) | FHHVIBPVBBRLOR-IYACIECVSA-N | 1.6094 | 0.68651 | 0.0020141 | 2.6959 | [M+H]+ | 794.6058 | 4.30 |
| 0.99_536.50 | NA | 2.3002 | 1.2017 | 0.0020916 | 2.6795 | [M+H]+ | 536.5023 | 0.99 |
| LysoPC(18:1) | YAMUFBLWGFFICM-PTGWMXDISA-N | 0.79667 | -0.32795 | 0.0022638 | 2.6452 | [M+H]+ | 522.3559 | 5.17 |
| 0.93_347.24 | NA | 5.0716 | 2.3424 | 0.0023539 | 2.6282 | [M+H-H2O]+ | 347.2419 | 0.93 |
| Proline | ONIBWKKTOPOVIA-BYPYZUCNSA-N | 1.4771 | 0.56272 | 0.0025545 | 2.5927 | [M+H]+ | 116.0696 | 7.44 |
| 4.58_432.31 | NA | 6.3961 | 2.6772 | 0.0026054 | 2.5841 | [M+H-H2O]+ | 432.3093 | 4.58 |
| 0.89_612.55 | NA | 1.8109 | 0.85668 | 0.0028009 | 2.5527 | [M+H]+ | 612.5537 | 0.89 |
| 1.18_490.37 | NA | 0.70748 | -0.49925 | 0.0032195 | 2.4922 | [M+H]+ | 490.3735 | 1.18 |
| PC(16:1/16:1) | GPWHCUUIQMGELX-VHQDNGOZSA-N | 0.75181 | -0.41156 | 0.0032737 | 2.485 | [M+H]+ | 730.5372 | 4.43 |
| 1.01_509.50 | NA | 4.4574 | 2.1562 | 0.0032737 | 2.485 | [M+H]+ | 509.5023 | 1.01 |
| PE(16:0/18:1) | FHQVHHIBKUMWTI-OTMQOFQLSA-N | 1.4353 | 0.52135 | 0.0041038 | 2.3868 | [M+H]+ | 718.5372 | 4.58 |
| N.epsilon.-Methyl-L-lysine | PQNASZJZHFPQLE-UHFFFAOYSA-N | 1.8484 | 0.88624 | 0.0041038 | 2.3868 | [M+H]+ | 161.1275 | 8.99 |
| 1.00_581.24 | NA | 0.51665 | -0.95274 | 0.0042745 | 2.3691 | [M+K]+ | 581.2378 | 1.00 |
| Lyso PAF C-16 | VLBPIWYTPAXCFJ-UHFFFAOYSA-N | 1.3151 | 0.39521 | 0.0043146 | 2.3651 | [M+H]+ | 482.3582 | 5.27 |
| 5.14_517.33 | NA | 12.35 | 3.6265 | 0.0044294 | 2.3537 | [M+H]+ | 517.3291 | 5.14 |
| LysoPC(16:0) | NA | 1.3057 | 0.38488 | 0.0047451 | 2.3238 | [M+H]+ | 496.3405 | 5.22 |
| 5.06_522.36 | NA | 0.71245 | -0.48914 | 0.0051345 | 2.2895 | [M+H]+ | 522.3557 | 5.06 |
| 1.08_558.47 | NA | 0.54813 | -0.86742 | 0.0053599 | 2.2708 | [M+NH4]+ | 558.4708 | 1.08 |
| 0.99_593.60 | NA | 0.58237 | -0.77999 | 0.0056395 | 2.2488 | [M+H]+ | 593.5972 | 0.99 |
| 1.12_535.40 | NA | 0.54669 | -0.87121 | 0.0063809 | 2.1951 | [M+NH4]+ | 535.3992 | 1.12 |
| 4.49_726.54 | NA | 0.67688 | -0.56302 | 0.0071769 | 2.1441 | [M+H]+ | 726.5413 | 4.49 |
| Methionine | FFEARJCKVFRZRR-BYPYZUCNSA-N | 2.628 | 1.394 | 0.0076592 | 2.1158 | [M+H]+ _[M+N-NH3]+ | 150.0575 _133.0308 | 7.16 |
| 1.09_484.40 | NA | 0.63975 | -0.64443 | 0.0077741 | 2.1093 | [M+NH4]+ | 484.3964 | 1.09 |
| 1.09_467.37 | NA | 0.597 | -0.74419 | 0.0089033 | 2.0504 | [M+H]+ | 467.3722 | 1.09 |
| [CAR(12:0)](https://www.ncbi.nlm.nih.gov/pcsubstance/?term=%22CAR(12%3A0)%22%5bCompleteSynonym%5d%20AND%20168381%5bStandardizedCID%5d) | FUJLYHJROOYKRA-QGZVFWFLSA-N | 0.45264 | -1.1435 | 0.0091634 | 2.0379 | [M+H]+ | 344.2791 | 3.69 |
| PC(O-16:0/22:6) | QQQQNYAHSSIZBU-HIQXTUQZSA-N | 0.47007 | -1.089 | 0.0096539 | 2.0153 | [M+H]+ | 792.5906 | 3.85 |
| 4.99_508.38 | NA | 0.75516 | -0.40514 | 0.0096539 | 2.0153 | [M+H]+ | 508.375 | 4.99 |
| Glycocholic acid | RFDAIACWWDREDC-FRVQLJSFSA-N | 3.3463 | 1.7425 | 0.0096539 | 2.0153 | [M+H]+ _[M+NH4]+ | 483.342 _466.3154 | 5.89 |
| 3.95_752.56 | NA | 0.43798 | -1.1911 | 0.0097492 | 2.011 | [M+H]+ | 752.5594 | 3.95 |
| PC(16:0/14:0) | UIXXHROAQSBBOV-PSXMRANNSA-N | 1.4733 | 0.55908 | 0.0099315 | 2.003 | [M+H]+ | 706.5375 | 4.46 |
| 1.07_590.48 | NA | 0.36984 | -1.435 | 0.009957 | 2.0019 | [M+H]+ | 590.4756 | 1.07 |
| 5.51_876.56 | NA | 1.3788 | 0.46342 | 0.010174 | 1.9925 | [M+H]+ | 876.5611 | 5.51 |
| 3.86_834.60 | NA | 0.53889 | -0.89193 | 0.01042 | 1.9821 | [M+H]+ | 834.6032 | 3.86 |
| 5.51_859.53 | NA | 1.3488 | 0.43169 | 0.01042 | 1.9821 | [M+H]+ | 859.5334 | 5.51 |
| Hippuric acid | QIAFMBKCNZACKA-UHFFFAOYSA-N | 0.4888 | -1.0327 | 0.011142 | 1.953 | [M+H]+ | 180.0645 | 3.38 |
| Carnitine | PHIQHXFUZVPYII-ZCFIWIBFSA-N | 1.3187 | 0.39913 | 0.011368 | 1.9443 | [M+H]+ _[M+Na]+ | 162.1127 _184.0937 | 7.48 |
| 1.12_530.44 | NA | 0.54093 | -0.8865 | 0.011789 | 1.9285 | [M+H]+ | 530.4398 | 1.12 |
| 0.90_855.74 | NA | 1.4127 | 0.49848 | 0.011789 | 1.9285 | [M+H]+ | 855.7403 | 0.90 |
| 4.34_706.54 | NA | 1.5463 | 0.62878 | 0.011789 | 1.9285 | [M+H]+ | 706.5372 | 4.34 |
| 4.09_806.57 | NA | 0.46259 | -1.1122 | 0.012115 | 1.9167 | [M+H]+ | 806.5707 | 4.09 |
| 4.59_450.32 | NA | 5.1065 | 2.3523 | 0.012196 | 1.9138 | [M+H]+ | 450.3201 | 4.59 |
| 5-Aminovaleric acid | JJMDCOVWQOJGCB-UHFFFAOYSA-N | 2.4665 | 1.3025 | 0.012615 | 1.8991 | [M+H-H2O]+ | 100.0752 | 1.50 |
| Betaine | NA | 1.3747 | 0.45909 | 0.012706 | 1.896 | [M+H]+ | 118.0863 | 6.98 |
| 1.00_561.53 | NA | 0.79599 | -0.32917 | 0.013599 | 1.8665 | [M+H]+ | 561.535 | 1.00 |
| 1.19_495.33 | NA | 0.7114 | -0.49127 | 0.01454 | 1.8374 | [M+K]+ | 495.3284 | 1.19 |
| 1.14_498.38 | NA | 0.57136 | -0.80754 | 0.015377 | 1.8131 | [M+NH4]+ | 498.3779 | 1.14 |
| 4.58_467.35 | NA | 4.6179 | 2.2072 | 0.015862 | 1.7996 | [M+NH4]+ | 467.3469 | 4.58 |
| [SM(d18:1/12:0)](https://www.ncbi.nlm.nih.gov/pcsubstance/?term=%22SM(d18%3A1%2F12%3A0)%22%5bCompleteSynonym%5d%20AND%2044260123%5bStandardizedCID%5d) | HZCLJRFPXMKWHR-FEBLJDHQSA-N | 0.74823 | -0.41844 | 0.01686 | 1.7732 | [M+H]+ | 647.5109 | 4.99 |
| 7-a-OH-3-oxo-Cholest-4-en-26-oic acid | SATGKQGFUDXGAX-SLTBQWEQSA-N | 1.5544 | 0.63632 | 0.016956 | 1.7707 | [M+H]+ | 431.3148 | 1.21 |
| 1.01_533.50 | NA | 2.2536 | 1.1722 | 0.017936 | 1.7463 | [M+H]+ | 533.5018 | 1.01 |
| Glycoursodeoxycholic acid | GHCZAUBVMUEKKP-TVQURETASA-N | 3.4105 | 1.77 | 0.017936 | 1.7463 | [M+H-H2O]+ | 432.3095 | 4.71 |
| 4.12_808.58 | NA | 0.59533 | -0.74825 | 0.019255 | 1.7155 | [M+NH4]+ | 808.5849 | 4.12 |
| 4.71_467.35 | NA | 4.6032 | 2.2026 | 0.019665 | 1.7063 | [M+NH4]+ | 467.3469 | 4.71 |
| 3.59_806.56 | NA | 9.5029 | 3.2484 | 0.021804 | 1.6615 | [M+H]+ | 806.5649 | 3.59 |
| 1.10_610.50 | NA | 0.6264 | -0.67485 | 0.021848 | 1.6606 | [M+NH4]+ | 610.5033 | 1.10 |
| 1.09_611.50 | NA | 0.62514 | -0.67776 | 0.023239 | 1.6338 | [M+H]+ | 611.5043 | 1.09 |
| 3.68_752.56 | NA | 1.4323 | 0.51834 | 0.024429 | 1.6121 | [M+H]+ | 752.5611 | 3.68 |
| 5.26_459.25 | NA | 1.2641 | 0.33816 | 0.024866 | 1.6044 | [M+Na]+ | 459.2493 | 5.26 |
| 1.11_465.36 | NA | 0.56038 | -0.83553 | 0.025615 | 1.5915 | [M+K]+ | 465.3566 | 1.11 |
| 5.53_880.59 | NA | 1.3875 | 0.47249 | 0.025615 | 1.5915 | [M+H]+ | 880.5926 | 5.53 |
| 4.33_704.52 | NA | 1.4312 | 0.51723 | 0.026407 | 1.5783 | [M-H]- | 704.5217 | 4.33 |
| PC(P-18:0/22:6) | TXHZYNSTTCIWMJ-RXSQUPBGSA-N | 1.5795 | 0.65944 | 0.030166 | 1.5205 | [M+H]+ | 818.605 | 4.28 |
| PC(18:0/22:6) | FAUYAENFVCNTAL-PFFNLMTBSA-N | 1.4693 | 0.55516 | 0.033211 | 1.4787 | [M+H]+ | 834.601 | 4.31 |
| CAR(16:0) | XOMRRQXKHMYMOC-OAQYLSRUSA-N | 0.58488 | -0.77378 | 0.035685 | 1.4475 | [M+H]+ | 400.3417 | 3.14 |
| 5.53_863.56 | NA | 1.3499 | 0.43288 | 0.037328 | 1.428 | [M+H]+ | 863.5644 | 5.53 |
| 4.71_450.32 | NA | 3.6375 | 1.863 | 0.037577 | 1.4251 | [M+H]+ | 450.3206 | 4.71 |
| 1.09_595.49 | NA | 0.58852 | -0.76483 | 0.039665 | 1.4016 | [M+H]+ | 595.494 | 1.09 |
| 1.13_463.34 | NA | 0.66275 | -0.59347 | 0.040435 | 1.3932 | [M+NH4]+ | 463.3411 | 1.13 |
| LysoPC(P-16:0) | HTZINLFNXLXRBC-CQLBIITFSA-N | 0.77154 | -0.37418 | 0.040435 | 1.3932 | [M+H]+ | 480.3446 | 5.05 |
| 5.26_476.28 | NA | 1.2362 | 0.30592 | 0.040435 | 1.3932 | [M+NH4]+ | 476.2762 | 5.26 |
| Leucine | ROHFNLRQFUQHCH-YFKPBYRVSA-N | 1.2424 | 0.31312 | 0.040965 | 1.3876 | [M+H]+ | 132.1013 | 6.76 |
| SM(d18:1/18:0) | LKQLRGMMMAHREN-YJFXYUILSA-N | 3.6618 | 1.8725 | 0.042682 | 1.3698 | [M+H]+ _[M+Na]+ | 731.6044 _753.5881 | 4.92 |
| 3.89_752.56 | NA | 0.49714 | -1.0083 | 0.044681 | 1.3499 | [M+H]+ | 752.561 | 3.89 |
| 1.08_469.39 | NA | 0.67654 | -0.56375 | 0.044817 | 1.3486 | [M-H+2Li]+ | 469.3884 | 1.08 |
| 4.30_792.59 | NA | 1.3796 | 0.46428 | 0.044817 | 1.3486 | [M+H]+ | 792.59 | 4.30 |
| [PC(O-16:0/20:4)](https://www.ncbi.nlm.nih.gov/pcsubstance/?term=%22PC(O-16%3A0%2F20%3A4(5Z%2C8Z%2C11Z%2C14Z))%22%5bCompleteSynonym%5d%20AND%206443139%5bStandardizedCID%5d) | NA | 1.2813 | 0.35766 | 0.048851 | 1.3111 | [M+H]+ | 768.5897 | 4.33 |
| [CAR(10:0)](https://www.ncbi.nlm.nih.gov/pcsubstance/?term=%22CAR(10%3A0)%22%5bCompleteSynonym%5d%20AND%2010245190%5bStandardizedCID%5d) | NA | 0.63626 | -0.65231 | 0.051466 | 1.2885 | [M+H]+ | 316.2478 | 4.11 |
| 0.91_565.40 | NA | 0.74674 | -0.42133 | 0.051466 | 1.2885 | [M+H]+ | 565.4021 | 0.91 |
| Trigonelline | WWNNZCOKKKDOPX-UHFFFAOYSA-N | 0.44383 | -1.1719 | 0.054675 | 1.2622 | [M+H]+ | 138.0544 | 7.20 |
| 3-Methylhistidine | JDHILDINMRGULE-LURJTMIESA-N | 1.7494 | 0.80683 | 0.055983 | 1.2519 | [M+H]+ | 170.0917 | 9.10 |
| 1.06_596.50 | NA | 0.6278 | -0.67162 | 0.057462 | 1.2406 | [M+NH4]+ | 596.5042 | 1.06 |
| Palmitoleoyl ethanolamide | WFRLANWAASSSFV-FPLPWBNLSA-N | 1.3027 | 0.38155 | 0.057462 | 1.2406 | [M+H]+ | 298.274 | 1.02 |
| 1.09_593.48 | NA | 0.69228 | -0.53056 | 0.065682 | 1.1826 | [M+H]+ | 593.478 | 1.09 |
| 4.00_794.61 | NA | 0.51541 | -0.9562 | 0.066688 | 1.176 | [M+H]+ | 794.6068 | 4.00 |
| 5.27_526.26 | NA | 0.38374 | -1.3818 | 0.069315 | 1.1592 | [M+H]+ | 526.2637 | 5.27 |
| 4.35_780.55 | NA | 0.80565 | -0.31177 | 0.070296 | 1.1531 | [M+K]+ | 780.5539 | 4.35 |
| Acetylcarnitine | RDHQFKQIGNGIED-MRVPVSSYSA-O | 1.3167 | 0.39688 | 0.074738 | 1.1265 | [M+H]+ | 204.1234 | 6.95 |
| 1.08_597.51 | NA | 0.7075 | -0.49919 | 0.076609 | 1.1157 | [M+H]+ | 597.5082 | 1.08 |
| 5.20_468.31 | NA | 0.71194 | -0.49016 | 0.081911 | 1.0867 | [M+H]+ | 468.3072 | 5.20 |
| 1.09_612.52 | NA | 0.68312 | -0.54979 | 0.089909 | 1.0462 | [M+NH4]+ | 612.5181 | 1.09 |

**Table S2:** Significant annotated metabolites comparing WD to HC. Metabolites were selected by volcano plot with fold change (FC) >1.2 and FDR-adjusted *p* <0.1 indicating significance.

| Metabolite Name/Identifier | InChI Key | FC | log2  (FC) | FDR  *p*-value | -log10  (p) | Species | m/z | RT |
| --- | --- | --- | --- | --- | --- | --- | --- | --- |
| Choline | OEYIOHPDSNJKLS-UHFFFAOYSA-N | 4.0012 | 2.0004 | 1.39E-16 | 15.857 | [M+H]+ | 104.1066 | 5.12 |
| Phenylalanine | COLNVLDHVKWLRT-QMMMGPOBSA-N | 2.2828 | 1.1908 | 8.69E-11 | 10.061 | [M+H]+ | 166.0862 | 6.61 |
| 2-Hydroxyphenethylamine | ULSIYEODSMZIPX-UHFFFAOYSA-N | 2.2469 | 1.1679 | 1.27E-10 | 9.895 | [M+H-H2O]+ | 120.0804 | 6.61 |
| PS(18:0/20:4) | SVOUGFFDROZBJI-DNALCEECSA-N | 0.37007 | -1.4341 | 1.75E-09 | 8.7569 | [M+H]+ | 812.5433 | 5.4 |
| S1P | DUYSYHSSBDVJSM-KRWOKUGFSA-N | 0.63312 | -0.65946 | 9.11E-09 | 8.0405 | [M+H]+ | 380.2552 | 5.96 |
| LysoPC(24:0) | SKJMUADLQLZAGH-WJOKGBTCSA-N | 0.69909 | -0.51645 | 0.00000118 | 5.9283 | [M+H]+ | 608.4634 | 5.03 |
| LysoPC(P-18:0) | WBOMIOWRFSPZMC-AYICAFKVSA-N | 0.53166 | -0.91143 | 0.00000211 | 5.6766 | [M+H]+ | 508.3748 | 5.22 |
| LysoPC(14:0) | VXUOFDJKYGDUJI-OAQYLSRUSA-N | 0.54089 | -0.88658 | 0.0000405 | 4.3922 | [M+H]+ | 468.3086 | 5.29 |
| Methionine | FFEARJCKVFRZRR-BYPYZUCNSA-N | 2.4829 | 1.312 | 0.0000824 | 4.084 | [M+H]+ _[M+N-NH3]+ | 150.0575 _133.0308 | 7.16 |
| PC(18:3/18:3) | XXKFQTJOJZELMD-JICBSJGISA-N | 0.55151 | -0.85853 | 0.0001062 | 3.9738 | [M+H]+ | 778.5374 | 4.18 |
| Hippuric acid | QIAFMBKCNZACKA-UHFFFAOYSA-N | 0.46299 | -1.111 | 0.0001276 | 3.8941 | [M+H]+ | 180.0645 | 3.38 |
| PC(18:1/14:0) | KIVAJCJTVPWSRJ-OQHNRNOKSA-N | 1.7027 | 0.76784 | 0.0004403 | 3.3562 | [M+H]+ | 732.5534 | 4.43 |
| SM(d18:1/18:1) | NBEADXWAAWCCDG-QDDWGVBQSA-N | 0.79652 | -0.32822 | 0.000517 | 3.2866 | [M+H]+ | 729.5913 | 4.93 |
| LysoPC(18:1) | YAMUFBLWGFFICM-PTGWMXDISA-N | 0.74449 | -0.42567 | 0.0007722 | 3.1123 | [M+H]+ | 522.3559 | 5.17 |
| Kynurenine | YGPSJZOEDVAXAB-QMMMGPOBSA-N | 1.4163 | 0.50211 | 0.0012296 | 2.9103 | [M+H]+ | 209.0914 | 6.62 |
| Tyrosine | OUYCCCASQSFEME-QMMMGPOBSA-N | 1.6318 | 0.70646 | 0.001598 | 2.7964 | [M+H]+ | 182.0802 | 7.39 |
| PC(14:0/18:0) | TYAQXZHDAGZOEO-KXQOOQHDSA-N | 1.9597 | 0.97061 | 0.0016193 | 2.7907 | [M+H]+ | 734.5685 | 4.45 |
| Betaine | KWIUHFFTVRNATP-UHFFFAOYSA-N | 1.2811 | 0.35743 | 0.001999 | 2.6992 | [M+H]+ | 118.0863 | 6.98 |
| SM(d18:1/12:0) | HZCLJRFPXMKWHR-FEBLJDHQSA-N | 0.70579 | -0.5027 | 0.0020034 | 2.6982 | [M+H]+ | 647.5109 | 4.99 |
| Histidine | HNDVDQJCIGZPNO-YFKPBYRVSA-N | 1.6583 | 0.72974 | 0.0035973 | 2.444 | [M+H]+ | 156.0762 | 9.07 |
| PC(O-16:0/22:6) | QQQQNYAHSSIZBU-HIQXTUQZSA-N | 0.45367 | -1.1403 | 0.0041881 | 2.378 | [M+H]+ | 792.5906 | 3.85 |
| PC(P-18:0/20:4) | FHHVIBPVBBRLOR-IYACIECVSA-N | 1.4915 | 0.57678 | 0.0056143 | 2.2507 | [M+H]+ | 794.6058 | 4.3 |
| 7-a-OH-3-oxo-Cholest-4-en-26-oic acid | SATGKQGFUDXGAX-SLTBQWEQSA-N | 1.4507 | 0.53671 | 0.0062521 | 2.204 | [M+H]+ | 431.3148 | 1.21 |
| PC(16:1/16:1) | GPWHCUUIQMGELX-VHQDNGOZSA-N | 0.69923 | -0.51616 | 0.0075823 | 2.1202 | [M+H]+ | 730.5372 | 4.43 |
| CAR(12:0) | FUJLYHJROOYKRA-QGZVFWFLSA-N | 0.42552 | -1.2327 | 0.0078147 | 2.1071 | [M+H]+ | 344.2791 | 3.69 |
| Ornithine | AHLPHDHHMVZTML-BYPYZUCNSA-N | 2.179 | 1.1237 | 0.012353 | 1.9082 | [M+H]+ | 133.0961 | 9.27 |
| 4-Aminomethylcyclo hexane  carboxylic acid | GYDJEQRTZSCIOI-LJGSYFOKSA-N | 0.76913 | -0.3787 | 0.015054 | 1.8223 | [M-H]- | 158.1166 | 6.62 |
| PHE-PHE | GKZIWHRNKRBEOH-UHFFFAOYSA-N | 0.64583 | -0.63078 | 0.015136 | 1.82 | [M+H]+ | 313.1546 | 5.57 |
| LysoPC(15:0) | RJZVWDTYEWCUAR-JOCHJYFZSA-N | 0.28009 | -1.8361 | 0.015555 | 1.8081 | [M+H]+ _[M+Na]+ | 482.3215 _504.3075 | 5.27 |
| Proline | ONIBWKKTOPOVIA-BYPYZUCNSA-N | 1.3805 | 0.46521 | 0.015555 | 1.8081 | [M+H]+ | 116.0696 | 7.44 |
| N-Methylproline | CWLQUGTUXBXTLF-YFKPBYRVSA-N | 1.7086 | 0.77284 | 0.020158 | 1.6956 | [M+H]+ | 130.0856 | 6.87 |
| PC(P-18:0/22:6) | TXHZYNSTTCIWMJ-RXSQUPBGSA-N | 1.4507 | 0.53671 | 0.020538 | 1.6874 | [M+H]+ | 818.605 | 4.28 |
| Alanine | QNAYBMKLOCPYGJ-REOHCLBHSA-N | 1.3144 | 0.39441 | 0.020763 | 1.6827 | [M+H]+ | 90.0542 | 7.79 |
| LysoPC(16:0).2 | ASWBNKHCZGQVJV-HSZRJFAPSA-N | 1.2194 | 0.28614 | 0.020763 | 1.6827 | [M+H]+ | 518.3223 | 5.24 |
| SM(d18:1/18:0) | LKQLRGMMMAHREN-YJFXYUILSA-N | 3.3044 | 1.7244 | 0.020763 | 1.6827 | [M+H]+ _[M+Na]+ | 731.6044 _753.5881 | 4.92 |
| PC(16:0/14:0) | UIXXHROAQSBBOV-PSXMRANNSA-N | 1.372 | 0.45623 | 0.020763 | 1.6827 | [M+H]+ | 706.5375 | 4.46 |
| PC(18:0/22:6) | FAUYAENFVCNTAL-PFFNLMTBSA-N | 1.3482 | 0.43104 | 0.021426 | 1.6691 | [M+H]+ | 834.601 | 4.31 |
| LysoPC(P-16:0) | HTZINLFNXLXRBC-CQLBIITFSA-N | 0.72122 | -0.4715 | 0.025922 | 1.5863 | [M+H]+ | 480.3446 | 5.05 |
| CAR(16:0) | XOMRRQXKHMYMOC-OAQYLSRUSA-N | 0.55595 | -0.84697 | 0.025922 | 1.5863 | [M+H]+ | 400.3417 | 3.14 |
| 5-Aminopentanoic acid | JJMDCOVWQOJGCB-UHFFFAOYSA-N | 2.2459 | 1.1673 | 0.025922 | 1.5863 | [M+H-H2O]+ | 100.0752 | 1.5 |
| Glycocholic acid | RFDAIACWWDREDC-FRVQLJSFSA-N | 3.2332 | 1.6929 | 0.027337 | 1.5633 | [M+H]+ _[M+NH4]+ | 483.342 _466.3154 | 5.89 |
| Glycoursodeoxycholic acid | GHCZAUBVMUEKKP-TVQURETASA-N | 3.3015 | 1.7231 | 0.027337 | 1.5633 | [M+H-H2O]+ | 432.3095 | 4.71 |
| Trigonelline | WWNNZCOKKKDOPX-UHFFFAOYSA-N | 0.43348 | -1.206 | 0.027337 | 1.5633 | [M+H]+ | 138.0544 | 7.2 |
| CAR(10:0) | LZOSYCMHQXPBFU-OAHLLOKOSA-N | 0.59019 | -0.76075 | 0.027337 | 1.5633 | [M+H]+ | 316.2478 | 4.11 |
| 3-Methylhistidine | JDHILDINMRGULE-LURJTMIESA-N | 1.6305 | 0.7053 | 0.048372 | 1.3154 | [M+H]+ | 170.0917 | 9.1 |
| LysoPE(18:1) | PYVRVRFVLRNJLY-MZMPXXGTSA-N | 0.83298 | -0.26365 | 0.048552 | 1.3138 | [M+H]+ | 480.308 | 5.44 |
| Carnitine | PHIQHXFUZVPYII-ZCFIWIBFSA-N | 1.2213 | 0.28843 | 0.053572 | 1.2711 | [M+H]+ _[M+Na]+ | 162.1127 _184.0937 | 7.48 |
| Palmitoleoyl Ethanolamide | WFRLANWAASSSFV-FPLPWBNLSA-N | 1.2205 | 0.28748 | 0.082333 | 1.0844 | [M+H]+ | 298.274 | 1.02 |

**Table S3:** Significant annotated metabolites comparing WD pre-clinical to HC. Metabolites were selected by volcano plot with fold change (FC) >1.2 and FDR-adjusted *p* <0.1 indicating significance.

| Metabolite Name/Identifier | InChI Key | FC | log2  (FC) | FDR  *p*-value | -log10  (p) | Species | m/z | RT |
| --- | --- | --- | --- | --- | --- | --- | --- | --- |
| Phenylalanine | COLNVLDHVKWLRT-QMMMGPOBSA-N | 2.1545 | 1.1073 | 1.35E-08 | 7.8688 | [M+H]+ | 166.0862 | 6.61 |
| 2-Hydroxy  phenethylamine | ULSIYEODSMZIPX-UHFFFAOYSA-N | 2.1174 | 1.0823 | 1.35E-08 | 7.8688 | [M+H-H2O]+ | 120.0804 | 6.61 |
| Choline | OEYIOHPDSNJKLS-UHFFFAOYSA-N | 3.3713 | 1.7533 | 0.00012916 | 3.8889 | [M+H]+ | 104.1066 | 5.12 |
| LysoPC(14:0) | VXUOFDJKYGDUJI-OAQYLSRUSA-N | 0.48547 | -1.0425 | 0.0005243 | 3.2804 | [M+H]+ | 468.3086 | 5.29 |
| LysoPC(18:1) | YAMUFBLWGFFICM-PTGWMXDISA-N | 0.72554 | -0.46288 | 0.0026638 | 2.5745 | [M+H]+ | 522.3559 | 5.17 |
| LysoPC(24:0) | SKJMUADLQLZAGH-WJOKGBTCSA-N | 0.63566 | -0.65366 | 0.0038754 | 2.4117 | [M+H]+ | 608.4634 | 5.03 |
| S1P | DUYSYHSSBDVJSM-KRWOKUGFSA-N | 0.66126 | -0.59671 | 0.0058796 | 2.2306 | [M+H]+ | 380.2552 | 5.96 |
| LysoPC(P-18:0) | WBOMIOWRFSPZMC-AYICAFKVSA-N | 0.61776 | -0.69488 | 0.0072996 | 2.1367 | [M+H]+ | 482.3235 | 5.42 |
| SM(d18:1/12:0) | HZCLJRFPXMKWHR-FEBLJDHQSA-N | 0.61614 | -0.69868 | 0.0084358 | 2.0739 | [M+H]+ | 647.5109 | 4.99 |
| PC(16:1/16:1) | GPWHCUUIQMGELX-VHQDNGOZSA-N | 0.66569 | -0.58708 | 0.01034 | 1.9855 | [M+H]+ | 730.5372 | 4.43 |
| PC(18:1/14:0) | KIVAJCJTVPWSRJ-OQHNRNOKSA-N | 1.5029 | 0.58776 | 0.013734 | 1.8622 | [M+H]+ | 732.5534 | 4.43 |
| PC(18:3/18:3) | XXKFQTJOJZELMD-JICBSJGISA-N | 0.61953 | -0.69075 | 0.013734 | 1.8622 | [M+H]+ | 778.5374 | 4.18 |
| LysoPC(P-16:0) | HTZINLFNXLXRBC-CQLBIITFSA-N | 0.5871 | -0.76832 | 0.013734 | 1.8622 | [M+H]+ | 508.3748 | 5.22 |
| PS(18:0/20:4) | SVOUGFFDROZBJI-DNALCEECSA-N | 0.37882 | -1.4004 | 0.013734 | 1.8622 | [M+H]+ | 812.5433 | 5.4 |
| Hippuric acid | QIAFMBKCNZACKA-UHFFFAOYSA-N | 0.26282 | -1.9279 | 0.013734 | 1.8622 | [M+H]+ | 180.0645 | 3.38 |
| PC(14:0/18:0) | TYAQXZHDAGZOEO-KXQOOQHDSA-N | 1.6287 | 0.70372 | 0.017233 | 1.7636 | [M+H]+ | 678.5046 | 4.49 |
| PC(O-16:0/22:6) | QQQQNYAHSSIZBU-HIQXTUQZSA-N | 0.32436 | -1.6244 | 0.017504 | 1.7569 | [M+H]+ | 792.5906 | 3.85 |
| 3-Methylhistidine | JDHILDINMRGULE-LURJTMIESA-N | 1.7099 | 0.77394 | 0.01771 | 1.7518 | [M+H]+ | 170.0917 | 9.1 |
| SM(d18:1/18:0) | LKQLRGMMMAHREN-YJFXYUILSA-N | 4.6239 | 2.2091 | 0.026926 | 1.5698 | [M+H]+ _[M+Na]+ | 731.6044 _753.5881 | 4.92 |
| Ornithine | AHLPHDHHMVZTML-BYPYZUCNSA-N | 1.8425 | 0.88165 | 0.031326 | 1.5041 | [M+H]+ | 133.0961 | 9.27 |
| SM(d18:1/24:1) | UYDFMJRVHYJLQZ-KVICREPESA-N | 2.9102 | 1.5411 | 0.032995 | 1.4816 | [M+H]+ | 813.6819 | 4.89 |
| PHE-PHE | GKZIWHRNKRBEOH-UHFFFAOYSA-N | 0.46692 | -1.0988 | 0.032995 | 1.4816 | [M+H]+ | 313.1546 | 5.57 |
| CAR(16:0) | XOMRRQXKHMYMOC-OAQYLSRUSA-N | 0.39103 | -1.3546 | 0.032995 | 1.4816 | [M+H]+ | 400.3417 | 3.14 |
| LysoPC(15:0) | RJZVWDTYEWCUAR-JOCHJYFZSA-N | 0.29578 | -1.7574 | 0.032995 | 1.4816 | [M+H]+ _[M+Na]+ | 482.3215 _504.3075 | 5.27 |
| Trigonelline | WWNNZCOKKKDOPX-UHFFFAOYSA-N | 0.27292 | -1.8734 | 0.032995 | 1.4816 | [M+H]+ | 138.0544 | 7.2 |
| Alanine | QNAYBMKLOCPYGJ-REOHCLBHSA-N | 1.3097 | 0.38919 | 0.034779 | 1.4587 | [M+H]+ | 90.0542 | 7.79 |
| Kynurenine | YGPSJZOEDVAXAB-QMMMGPOBSA-N | 1.2392 | 0.30943 | 0.043007 | 1.3665 | [M+H]+ | 209.0914 | 6.62 |
| Histidine | HNDVDQJCIGZPNO-YFKPBYRVSA-N | 1.3423 | 0.42472 | 0.045202 | 1.3448 | [M+H]+ | 156.0762 | 9.07 |
| Proline | ONIBWKKTOPOVIA-BYPYZUCNSA-N | 1.3413 | 0.42368 | 0.047436 | 1.3239 | [M+H]+ | 116.0696 | 7.44 |
| Lyso(PE18:1) | PYVRVRFVLRNJLY-MZMPXXGTSA-N | 0.71988 | -0.47418 | 0.047436 | 1.3239 | [M+H]+ | 480.308 | 5.44 |
| Methionine | FFEARJCKVFRZRR-BYPYZUCNSA-N | 1.3041 | 0.38304 | 0.053444 | 1.2721 | [M+H]+ _[M+N-NH3]+ | 150.0575 _133.0308 | 7.16 |
| Gal ceramide (d18:1/24:1) | WBOZIXHPUPAOIA-YEWIENRVSA-N | 1.628 | 0.70311 | 0.060268 | 1.2199 | [M+H]+ | 810.6806 | 1.46 |
| PC(O-18:1/2:0) | NA | 0.73524 | -0.44372 | 0.060268 | 1.2199 | [M+H]+ | 550.3851 | 5.01 |
| LysoPC(18:0) | ATHVAWFAEPLPPQ-QPOMNCEOSA-N | 0.70519 | -0.50393 | 0.060268 | 1.2199 | [M+H]+ | 506.3592 | 5.02 |
| N6-Methyllysine | PQNASZJZHFPQLE-UHFFFAOYSA-N | 1.3341 | 0.41584 | 0.077546 | 1.1104 | [M+H]+ | 161.1275 | 8.99 |
| Carnitine | PHIQHXFUZVPYII-ZCFIWIBFSA-N | 1.2445 | 0.31559 | 0.0819 | 1.0867 | [M+H]+ _[M+Na]+ | 162.1127 _184.0937 | 7.48 |
| PC(P-18:0/20:4) | FHHVIBPVBBRLOR-IYACIECVSA-N | 1.3596 | 0.44317 | 0.082391 | 1.0841 | [M+H]+ | 794.6058 | 4.3 |

**Table S4:** Significant annotated metabolites comparing WD neurologic to HC. Metabolites were selected by volcano plot with fold change (FC) >1.2 and FDR-adjusted *p* <0.1 indicating significance.

| Metabolite Name/Identifier | InChI Key | FC | log2  (FC) | FDR  *p*-value | -log10  (p) | Species | m/z | RT |
| --- | --- | --- | --- | --- | --- | --- | --- | --- |
| Choline | OEYIOHPDSNJKLS-UHFFFAOYSA-N | 3.9237 | 1.9722 | 2.15E-13 | 12.668 | [M+H]+ | 104.107 | 5.12 |
| Phenylalanine | COLNVLDHVKWLRT-QMMMGPOBSA-N | 2.1497 | 1.1041 | 6.94E-09 | 8.1585 | [M+H]+ | 166.086 | 6.61 |
| 2-Hydroxy phenethylamine | ULSIYEODSMZIPX-UHFFFAOYSA-N | 2.1188 | 1.0833 | 6.94E-09 | 8.1585 | [M+H-H2O]+ | 120.0804 | 6.61 |
| S1P | DUYSYHSSBDVJSM-KRWOKUGFSA-N | 0.60486 | -0.7253 | 1.20E-06 | 5.921 | [M+H]+ | 380.255 | 5.96 |
| PS(18:0/20:4) | SVOUGFFDROZBJI-DNALCEECSA-N | 0.31296 | -1.6759 | 6.43E-05 | 4.1919 | [M+H]+ | 812.543 | 5.4 |
| LysoPC(P-18:0) | WBOMIOWRFSPZMC-AYICAFKVSA-N | 0.58891 | -0.7639 | 0.0002 | 3.6889 | [M+H]+ | 482.324 | 5.42 |
| Methionine | FFEARJCKVFRZRR-BYPYZUCNSA-N | 1.5205 | 0.60458 | 0.00056 | 3.2522 | [M+H]+ _[M+N-NH3]+ | 150.0575 _133.0308 | 7.16 |
| PC(18:1/14:0) | KIVAJCJTVPWSRJ-OQHNRNOKSA-N | 1.6642 | 0.73486 | 0.00131 | 2.8839 | [M+H]+ | 732.553 | 4.43 |
| PC(14:0/18:0) | TYAQXZHDAGZOEO-KXQOOQHDSA-N | 1.8995 | 0.9256 | 0.00215 | 2.6685 | [M+H]+ | 734.569 | 4.45 |
| LysoPC(24:0) | SKJMUADLQLZAGH-WJOKGBTCSA-N | 0.73279 | -0.4485 | 0.00259 | 2.5873 | [M+H]+ | 608.463 | 5.03 |
| LysoPC(14:0) | VXUOFDJKYGDUJI-OAQYLSRUSA-N | 0.58288 | -0.7787 | 0.00259 | 2.5873 | [M+H]+ | 468.309 | 5.29 |
| Kynurenine | YGPSJZOEDVAXAB-QMMMGPOBSA-N | 1.3776 | 0.46218 | 0.00301 | 2.5221 | [M+H]+ | 209.091 | 6.62 |
| Tyrosine | OUYCCCASQSFEME-QMMMGPOBSA-N | 1.5647 | 0.64589 | 0.00395 | 2.4036 | [M+H]+ | 182.08 | 7.39 |
| PC(18:3/18:3) | XXKFQTJOJZELMD-JICBSJGISA-N | 0.57205 | -0.8058 | 0.00395 | 2.4036 | [M+H]+ | 778.537 | 4.18 |
| LysoPC(18:1) | YAMUFBLWGFFICM-PTGWMXDISA-N | 0.75887 | -0.3981 | 0.00467 | 2.3308 | [M+H]+ | 522.356 | 5.17 |
| SM(d18:1/18:1) | NBEADXWAAWCCDG-QDDWGVBQSA-N | 0.80369 | -0.3153 | 0.00561 | 2.2514 | [M+H]+ | 729.591 | 4.93 |
| Histidine | HNDVDQJCIGZPNO-YFKPBYRVSA-N | 1.5291 | 0.61269 | 0.01307 | 1.8837 | [M+H]+ | 156.076 | 9.07 |
| PC(P-18:0/20:4) | FHHVIBPVBBRLOR-IYACIECVSA-N | 1.4379 | 0.52401 | 0.01663 | 1.7792 | [M+H]+ | 794.606 | 4.3 |
| PC(18:0/22:6) | FAUYAENFVCNTAL-PFFNLMTBSA-N | 1.4297 | 0.51567 | 0.01887 | 1.7243 | [M+H]+ | 834.601 | 4.31 |
| CAR(12:0) | FUJLYHJROOYKRA-QGZVFWFLSA-N | 0.35652 | -1.488 | 0.01887 | 1.7243 | [M+H]+ | 344.279 | 3.69 |
| LysoPC(16:0).2 | ASWBNKHCZGQVJV-HSZRJFAPSA-N | 1.205 | 0.26901 | 0.0191 | 1.7191 | [M+H]+ | 518.322 | 5.24 |
| Glycocholic acid | RFDAIACWWDREDC-FRVQLJSFSA-N | 2.6526 | 1.4074 | 0.01957 | 1.7085 | [M+H]+ _[M+NH4]+ | 483.342 _466.3154 | 5.89 |
| SM(d18:1/12:0) | HZCLJRFPXMKWHR-FEBLJDHQSA-N | 0.71532 | -0.4834 | 0.01957 | 1.7085 | [M+H]+ | 647.511 | 4.99 |
| Ornithine | AHLPHDHHMVZTML-BYPYZUCNSA-N | 1.9751 | 0.98194 | 0.02315 | 1.6355 | [M+H]+ | 133.096 | 9.27 |
| PC(P-18:0/22:6) | FAUYAENFVCNTAL-PFFNLMTBSA-N | 1.5152 | 0.5995 | 0.02869 | 1.5423 | [M+H]+ | 818.605 | 4.28 |
| SM(d18:1/18:0) | LKQLRGMMMAHREN-YJFXYUILSA-N | 3.4924 | 1.8042 | 0.03491 | 1.4571 | [M+H]+ _[M+Na]+ | 731.6044 _753.5881 | 4.92 |
| LysoPC(P-16:0) | HTZINLFNXLXRBC-CQLBIITFSA-N | 0.69518 | -0.5246 | 0.0354 | 1.4511 | [M+H]+ | 508.375 | 5.22 |
| 1-Methylnicotinamide | LDHMAVIPBRSVRG-UHFFFAOYSA-O | 0.6415 | -0.6405 | 0.0354 | 1.4511 | [M+H-C4H8]+ | 137.07 | 6.02 |
| 4-Aminomethylcyclo hexanecarboxylic acid | GYDJEQRTZSCIOI-LJGSYFOKSA-N | 0.62293 | -0.6829 | 0.03789 | 1.4215 | [M-H]- | 158.117 | 6.62 |
| 7-a-OH-3-oxo-Cholest-4-en-26-oic acid | SATGKQGFUDXGAX-SLTBQWEQSA-N | 1.2746 | 0.35006 | 0.03818 | 1.4182 | [M+H]+ | 431.315 | 1.21 |
| PC(16:1/16:1) | GPWHCUUIQMGELX-VHQDNGOZSA-N | 0.74529 | -0.4241 | 0.03884 | 1.4107 | [M+H]+ | 730.537 | 4.43 |
| Hippuric acid | QIAFMBKCNZACKA-UHFFFAOYSA-N | 0.59177 | -0.7569 | 0.03884 | 1.4107 | [M+H]+ | 180.065 | 3.38 |
| Alanine | QNAYBMKLOCPYGJ-REOHCLBHSA-N | 1.2821 | 0.35849 | 0.04182 | 1.3787 | [M+H]+ | 90.0542 | 7.79 |
| Glycoursodeoxycholic acid | GHCZAUBVMUEKKP-TVQURETASA-N | 2.3972 | 1.2614 | 0.04663 | 1.3313 | [M+H-H2O]+ | 432.31 | 4.71 |
| Carnitine | PHIQHXFUZVPYII-ZCFIWIBFSA-N | 1.2558 | 0.32862 | 0.04663 | 1.3313 | [M+H]+ _[M+Na]+ | 162.1127 _184.0937 | 7.48 |
| PC(O-16:0/20:1).1 | NA | 0.82328 | -0.2805 | 0.04663 | 1.3313 | [M+H]+ | 524.372 | 5.16 |
| CAR(16:0) | XOMRRQXKHMYMOC-OAQYLSRUSA-N | 0.49048 | -1.0277 | 0.04663 | 1.3313 | [M+H]+ | 400.342 | 3.14 |
| Proline | ONIBWKKTOPOVIA-BYPYZUCNSA-N | 1.2783 | 0.35426 | 0.04709 | 1.3271 | [M+H]+ | 116.07 | 7.44 |
| PC(16:0/14:0) | UIXXHROAQSBBOV-PSXMRANNSA-N | 1.3549 | 0.43818 | 0.04726 | 1.3255 | [M+H]+ | 706.538 | 4.46 |
| LysoPC(15:0) | RJZVWDTYEWCUAR-JOCHJYFZSA-N | 0.37956 | -1.3976 | 0.04815 | 1.3174 | [M+H]+ _[M+Na]+ | 482.3215 _504.3075 | 5.27 |
| PC(O-16:0/22:6) | TXHZYNSTTCIWMJ-RXSQUPBGSA-N | 0.58631 | -0.7703 | 0.05322 | 1.2739 | [M+H]+ | 792.591 | 3.85 |
| N6-Methyllysine | PQNASZJZHFPQLE-UHFFFAOYSA-N | 1.4885 | 0.57382 | 0.0591 | 1.2284 | [M+H]+ | 161.128 | 8.99 |
| CAR(10:0) | LZOSYCMHQXPBFU-OAHLLOKOSA-N | 0.58469 | -0.7743 | 0.07324 | 1.1353 | [M+H]+ | 316.248 | 4.11 |
| SM(d18:1/24:1) | UYDFMJRVHYJLQZ-KVICREPESA-N | 2.2895 | 1.195 | 0.07844 | 1.1055 | [M+H]+ | 813.682 | 4.89 |
| PE(16:0/18:1) | FHQVHHIBKUMWTI-OTMQOFQLSA-N | 1.3741 | 0.45849 | 0.09024 | 1.0446 | [M+H]+ | 718.537 | 4.58 |

**Tabel S5:** Significant annotated metabolites comparing WD hepatic to HC. Metabolites were selected by volcano plot with fold- change (FC) >1.2 and FDR-adjusted *p* <0.1 indicating significance.

| Metabolite Name/Identifier | InChI Key | FC | log2  (FC) | FDR  *p*-value | -log10  (p) | Species | m/z | RT |
| --- | --- | --- | --- | --- | --- | --- | --- | --- |
| Choline | OEYIOHPDSNJKLS-UHFFFAOYSA-N | 4.3574 | 2.1235 | 2.18E-13 | 12.661 | [M+H]+ | 104.1066 | 5.12 |
| Phenylalanine | COLNVLDHVKWLRT-QMMMGPOBSA-N | 2.4547 | 1.2956 | 2.26E-08 | 7.646 | [M+H]+ | 166.0862 | 6.61 |
| 2-Hydroxy phenethylamine | ULSIYEODSMZIPX-UHFFFAOYSA-N | 2.415 | 1.2721 | 2.30E-08 | 7.6378 | [M+H-H2O]+ | 120.0804 | 6.61 |
| LysoPC(P-18:0) | WBOMIOWRFSPZMC-AYICAFKVSA-N | 0.44348 | -1.1731 | 4.53E-07 | 6.3443 | [M+H]+ | 508.3748 | 5.22 |
| LysoPC(24:0) | SKJMUADLQLZAGH-WJOKGBTCSA-N | 0.69985 | -0.51489 | 0.000182 | 3.7402 | [M+H]+ | 608.4634 | 5.03 |
| S1P | DUYSYHSSBDVJSM-KRWOKUGFSA-N | 0.64404 | -0.63478 | 0.000231 | 3.6359 | [M+H]+ | 380.2552 | 5.96 |
| PC(18:3/18:3) | XXKFQTJOJZELMD-JICBSJGISA-N | 0.50274 | -0.99211 | 0.000231 | 3.6359 | [M+H]+ | 778.5374 | 4.18 |
| LysoPC(14:0) | VXUOFDJKYGDUJI-OAQYLSRUSA-N | 0.53095 | -0.91336 | 0.000313 | 3.5043 | [M+H]+ | 468.3086 | 5.29 |
| PS(18:0/20:4) | FHHVIBPVBBRLOR-IYACIECVSA-N | 0.41435 | -1.2711 | 0.000313 | 3.5043 | [M+H]+ | 812.5433 | 5.4 |
| PC(18:1/14:0) | KIVAJCJTVPWSRJ-OQHNRNOKSA-N | 1.8275 | 0.86988 | 0.000493 | 3.3073 | [M+H]+ | 732.5534 | 4.43 |
| SM(d18:1/18:1) | NBEADXWAAWCCDG-QDDWGVBQSA-N | 0.7124 | -0.48924 | 0.000764 | 3.1167 | [M+H]+ | 729.5913 | 4.93 |
| Histidine | HNDVDQJCIGZPNO-YFKPBYRVSA-N | 1.9135 | 0.93624 | 0.001947 | 2.7106 | [M+H]+ | 156.0762 | 9.07 |
| LysoPC(18:1) | YAMUFBLWGFFICM-PTGWMXDISA-N | 0.74108 | -0.4323 | 0.001947 | 2.7106 | [M+H]+ | 522.3559 | 5.17 |
| Hippuric acid | QIAFMBKCNZACKA-UHFFFAOYSA-N | 0.44641 | -1.1636 | 0.003238 | 2.4897 | [M+H]+ | 180.0645 | 3.38 |
| PC(14:0/18:0) | TYAQXZHDAGZOEO-KXQOOQHDSA-N | 2.1634 | 1.1133 | 0.00351 | 2.4547 | [M+H]+ | 678.5046 | 4.49 |
| Tyrosine | OUYCCCASQSFEME-QMMMGPOBSA-N | 1.8955 | 0.92255 | 0.003674 | 2.4348 | [M+H]+ | 182.0802 | 7.39 |
| Methionine | FFEARJCKVFRZRR-BYPYZUCNSA-N | 3.8412 | 1.9416 | 0.004076 | 2.3898 | [M+H]+ _[M+N-NH3]+ | 150.0575 _133.0308 | 7.16 |
| 7-a-OH-3-oxo-Cholest-4-en-26-oic acid | SATGKQGFUDXGAX-SLTBQWEQSA-N | 1.8093 | 0.8554 | 0.004566 | 2.3405 | [M+H]+ | 431.3148 | 1.21 |
| PC(16:1/16:1) | GPWHCUUIQMGELX-VHQDNGOZSA-N | 0.67574 | -0.56546 | 0.005196 | 2.2843 | [M+H]+ | 730.5372 | 4.43 |
| Kynurenine | YGPSJZOEDVAXAB-QMMMGPOBSA-N | 1.5307 | 0.61421 | 0.006967 | 2.157 | [M+H]+ | 209.0914 | 6.62 |
| Betaine | KWIUHFFTVRNATP-UHFFFAOYSA-N | 1.4629 | 0.54886 | 0.006967 | 2.157 | [M+H]+ | 118.0863 | 6.98 |
| PC(O-16:0/22:6) | QQQQNYAHSSIZBU-HIQXTUQZSA-N | 0.40111 | -1.3179 | 0.008002 | 2.0968 | [M+H]+ | 792.5906 | 3.85 |
| Ornithine | AHLPHDHHMVZTML-BYPYZUCNSA-N | 2.5069 | 1.3259 | 0.008597 | 2.0656 | [M+H]+ | 133.0961 | 9.27 |
| LysoPC(15:0) | RJZVWDTYEWCUAR-JOCHJYFZSA-N | 0.18868 | -2.406 | 0.008981 | 2.0467 | [M+H]+ _[M+Na]+ | 482.3215 _504.3075 | 5.27 |
| PC(P-18:0/20:4) | FHHVIBPVBBRLOR-IYACIECVSA-N | 1.5977 | 0.67603 | 0.009536 | 2.0206 | [M+H]+ | 794.6058 | 4.3 |
| PC(20:4/20:4) | LZLVZIFMYXDKCN-QJWFYWCHSA-N | 0.34292 | -1.5441 | 0.009536 | 2.0206 | [M+H]+ | 830.5692 | 4 |
| LysoPC(16:0).2 | ASWBNKHCZGQVJV-HSZRJFAPSA-N | 1.3432 | 0.42563 | 0.01187 | 1.9255 | [M+H]+ | 518.3223 | 5.24 |
| N6-Methyllysine | PQNASZJZHFPQLE-UHFFFAOYSA-N | 2.0678 | 1.0481 | 0.013173 | 1.8803 | [M+H]+ | 161.1275 | 8.99 |
| Glycoursodeoxy cholic acid | GHCZAUBVMUEKKP-TVQURETASA-N | 5.1039 | 2.3516 | 0.013505 | 1.8695 | [M+H-H2O]+ | 432.3095 | 4.71 |
| PC(16:0/14:0) | UIXXHROAQSBBOV-PSXMRANNSA-N | 1.5046 | 0.5894 | 0.015233 | 1.8172 | [M+H]+ | 706.5375 | 4.46 |
| SM(d18:1/12:0) | HZCLJRFPXMKWHR-FEBLJDHQSA-N | 0.7391 | -0.43616 | 0.015233 | 1.8172 | [M+H]+ | 647.5109 | 4.99 |
| CAR(12:0) | FUJLYHJROOYKRA-QGZVFWFLSA-N | 0.43623 | -1.1969 | 0.015233 | 1.8172 | [M+H]+ | 344.2791 | 3.69 |
| Acetylcholine | OIPILFWXSMYKGL-UHFFFAOYSA-N | 1.499 | 0.58399 | 0.017776 | 1.7502 | [M+H]+ | 146.1168 | 7.52 |
| Palmitoleoyl Ethanolamide | WFRLANWAASSSFV-FPLPWBNLSA-N | 1.3211 | 0.40178 | 0.017776 | 1.7502 | [M+H]+ | 298.274 | 1.02 |
| Proline | ONIBWKKTOPOVIA-BYPYZUCNSA-N | 1.4851 | 0.57054 | 0.018759 | 1.7268 | [M+H]+ | 116.0696 | 7.44 |
| 5-Aminopentanoic acid | JJMDCOVWQOJGCB-UHFFFAOYSA-N | 2.7402 | 1.4543 | 0.019844 | 1.7024 | [M+H-H2O]+ | 100.0752 | 1.5 |
| Alanine | QNAYBMKLOCPYGJ-REOHCLBHSA-N | 1.3439 | 0.42646 | 0.019844 | 1.7024 | [M+H]+ | 90.0542 | 7.79 |
| CAR(10:0) | RFDAIACWWDREDC-FRVQLJSFSA-N | 0.5259 | -0.92714 | 0.021989 | 1.6578 | [M+H]+ | 316.2478 | 4.11 |
| Glycocholic acid | RFDAIACWWDREDC-FRVQLJSFSA-N | 4.9625 | 2.3111 | 0.022623 | 1.6454 | [M+H]+ _[M+NH4]+ | 483.342 _466.3154 | 5.89 |
| PHE-PHE | GKZIWHRNKRBEOH-UHFFFAOYSA-N | 0.60592 | -0.72279 | 0.024145 | 1.6172 | [M+H]+ | 313.1546 | 5.57 |
| Lyso PAF C-16 | VLBPIWYTPAXCFJ-UHFFFAOYSA-N | 1.4482 | 0.53426 | 0.036462 | 1.4382 | [M+H]+ | 482.3582 | 5.27 |
| Trigonelline | WWNNZCOKKKDOPX-UHFFFAOYSA-N | 138.0544 | -1.7781 | 0.038987 | 1.4091 | [M+H]+ | 138.0544 | 7.2 |
| Acetylcarnitine | RDHQFKQIGNGIED-MRVPVSSYSA-O | 1.3438 | 0.42632 | 0.042535 | 1.3713 | [M+H]+ | 204.1234 | 6.95 |
| PC(O-16:0/20:1).1 | NA | 0.82713 | -0.27381 | 0.042535 | 1.3713 | [M+H]+ | 524.3716 | 5.16 |
| PE(O-18:1/20:4) | URPXXNCTXCOATD-FXMFQVEGSA-N | 0.50772 | -0.9779 | 0.050332 | 1.2982 | [M+H]+ | 752.5604 | 3.81 |
| PC(P-18:0/22:6) | TXHZYNSTTCIWMJ-RXSQUPBGSA-N | 1.491 | 0.57628 | 0.051153 | 1.2911 | [M+H]+ | 818.605 | 4.28 |
| PC(O-16:0/20:4) | NA | 1.2836 | 0.36024 | 0.084826 | 1.0715 | [M+H]+ | 768.5897 | 4.33 |
| 3-Methylhistidine | JDHILDINMRGULE-LURJTMIESA-N | 1.9118 | 0.93494 | 0.096339 | 1.0162 | [M+H]+ | 170.0917 | 9.1 |
| PI(18:0/20:4) | KRTOMQDUKGRFDJ-MWZLTEOLSA-N | 0.82755 | -0.27308 | 0.096339 | 1.0162 | [M+H]+ _[M+NH4]+ | 904.5929 _887.5652 | 5.48 |

**Table S6:** Metabolite correlation analysis with choline. Correlation coefficients were calculated based on Pearson's correlation with *p* <0.05 indicating significance.

| Metabolite | correlation | *t-*stat | *p*-value | FDR *p*-value |
| --- | --- | --- | --- | --- |
| Phenylalanine | 0.78285 | 10.75 | 1.07E-16 | 3.95E-15 |
| LysoPC(14:0) | -0.67477 | -7.8116 | 3.18E-11 | 7.84E-10 |
| PC(14:0/18:0) | 0.65672 | 7.4403 | 1.58E-10 | 2.92E-09 |
| LysoPC(18:1) | -0.64136 | -7.1423 | 5.67E-10 | 8.39E-09 |
| PC(P-18:0/20:4) | 0.6345 | 7.0138 | 9.82E-10 | 1.21E-08 |
| PC(O-16:0/2:0).1 | -0.62883 | -6.9099 | 1.53E-09 | 1.62E-08 |
| S1P | -0.62261 | -6.798 | 2.46E-09 | 2.28E-08 |
| Histidine | 0.60254 | 6.4506 | 1.07E-08 | 8.79E-08 |
| LysoPC(P-18:0) | -0.59826 | -6.379 | 1.44E-08 | 1.07E-07 |
| PC(18:1/14:0) | 0.57975 | 6.0793 | 5.03E-08 | 3.23E-07 |
| LysoPC(24:0) | -0.5791 | -6.0691 | 5.24E-08 | 3.23E-07 |
| Ornithine | 0.56956 | 5.9205 | 9.67E-08 | 5.51E-07 |
| Alanine | 0.5419 | 5.509 | 5.14E-07 | 2.72E-06 |
| PC(P-18:0/22:6) | 0.53696 | 5.4383 | 6.82E-07 | 3.36E-06 |
| PC(O-16:0/22:6) | -0.52538 | -5.2755 | 1.30E-06 | 6.02E-06 |
| PC(O-16:0/2:0).2 | -0.51975 | -5.198 | 1.77E-06 | 7.68E-06 |
| CAR(12:0) | -0.51847 | -5.1805 | 1.89E-06 | 7.77E-06 |
| PS(18:0/20:4) | -0.50082 | -4.9436 | 4.74E-06 | 1.84E-05 |
| PC(O-16:0/20:4) | 0.49988 | 4.9313 | 4.97E-06 | 1.84E-05 |
| Tyrosine | 0.49827 | 4.9101 | 5.39E-06 | 1.90E-05 |
| Betaine | 0.49619 | 4.883 | 5.98E-06 | 2.01E-05 |
| Proline | 0.48422 | 4.7285 | 1.07E-05 | 3.46E-05 |
| Carnitine | 0.48105 | 4.6882 | 1.25E-05 | 3.85E-05 |
| SM(d18:1/18:1) | -0.47946 | -4.6681 | 1.35E-05 | 3.99E-05 |
| LysoPC(P-16:0) | -0.47674 | -4.6337 | 1.53E-05 | 4.36E-05 |
| Kynurenine | 0.46938 | 4.5418 | 2.16E-05 | 5.91E-05 |
| Methionine | 0.46714 | 4.5141 | 2.39E-05 | 6.31E-05 |
| PC(18:3/18:3) | -0.46356 | -4.4699 | 2.81E-05 | 7.17E-05 |
| LysoPC(15:0) | -0.45408 | -4.3545 | 4.28E-05 | 0.00010555 |
| PC(16:0/14:0) | 0.444 | 4.2337 | 6.60E-05 | 0.00015762 |
| PI(18:0/20:4) | -0.43925 | -4.1775 | 8.06E-05 | 0.00018649 |
| SM(d18:1/18:0) | 0.42464 | 4.0074 | 0.0001464 | 0.00032829 |
| CAR(16:0) | -0.41967 | -3.9503 | 0.00017826 | 0.00038798 |
| LysoPC(16:0).1 | -0.41242 | -3.868 | 0.00023618 | 0.00049934 |
| PC(18:1/18:1) | 0.40541 | 3.7892 | 0.00030829 | 0.0006337 |
| PC(O-18:1/2:0) | -0.38708 | -3.5868 | 0.00060209 | 0.0012042 |
| Gal ceramide (d18:1/24:1) | 0.38412 | 3.5546 | 0.00066844 | 0.0013017 |
| Glycoursodeoxycholic acid | 0.36504 | 3.3501 | 0.0012813 | 0.0024313 |
| PC(18:0/22:6) | 0.36292 | 3.3277 | 0.0013738 | 0.0025416 |
| LysoPC(16:0).2 | 0.36162 | 3.314 | 0.0014339 | 0.002588 |
| CAR(10:0) | -0.35951 | -3.2917 | 0.0015362 | 0.0027067 |
| Lyso(PE18:1) | -0.3479 | -3.1705 | 0.0022248 | 0.0037199 |
| Glycocholic acid | 0.34752 | 3.1666 | 0.0022514 | 0.0037199 |
| SMd(18:1/12:0) | -0.34737 | -3.165 | 0.0022621 | 0.0037199 |
| PC(16:1/16:1) | -0.34611 | -3.152 | 0.0023528 | 0.0037291 |
| PE(16:0/18:1) | 0.34532 | 3.1438 | 0.0024113 | 0.0037291 |
| PC(O-16:0/20:5) | -0.34522 | -3.1428 | 0.0024189 | 0.0037291 |
| CAR(18:1) | -0.33834 | -3.072 | 0.0029874 | 0.0045116 |
| Leucine | 0.33095 | 2.9965 | 0.0037292 | 0.0055192 |
| SMd(18:1/24:1) | 0.31426 | 2.8284 | 0.0060357 | 0.0087577 |
| PC(20:4/20:4) | -0.273 | -2.4246 | 0.0178 | 0.02533 |
| PC(18:0/18:1) | -0.27215 | -2.4164 | 0.018174 | 0.025374 |
| LysoPC(18:0) | -0.2604 | -2.3044 | 0.02405 | 0.032958 |
| PC(18:0/18:0) | 0.2553 | 2.256 | 0.027064 | 0.036339 |
| PE(O-18:1/20:4) | -0.2546 | -2.2494 | 0.0275 | 0.036339 |
| LysoPE(18:0) | -0.25156 | -2.2208 | 0.02947 | 0.03826 |
| Acetylcarnitine | 0.23095 | 2.0281 | 0.046202 | 0.058947 |
| Lyso PAF C-16 | 0.20636 | 1.802 | 0.075681 | 0.094922 |
| PC(16:0/18:2) | 0.19049 | 1.6579 | 0.10162 | 0.12534 |
| Acetylcholine | 0.18224 | 1.5836 | 0.11761 | 0.14267 |
| PC(20:1/20:1) | 0.11639 | 1.0012 | 0.32002 | 0.38196 |
| PE(16:0/18:2) | 0.098718 | 0.84759 | 0.39944 | 0.46918 |
| Isoleucine | 0.079209 | 0.6789 | 0.49935 | 0.57737 |
| PC(16:0/18:1) | 0.063271 | 0.54168 | 0.58969 | 0.67134 |
| PC(16:0/22:6) | -0.060903 | -0.52132 | 0.60372 | 0.6769 |
| TMAO | 0.036081 | 0.30848 | 0.7586 | 0.83785 |
| Cholesterol | 0.032133 | 0.27468 | 0.78434 | 0.85354 |
| PC(16:0/18:0) | 0.023331 | 0.1994 | 0.84251 | 0.90356 |
| Tryptophan | 0.020291 | 0.1734 | 0.86282 | 0.91212 |
| PE(18:0/22:6) | 0.017483 | 0.14939 | 0.88166 | 0.91891 |
| PC(14:0/14:0) | 0.015561 | 0.13297 | 0.89458 | 0.91943 |
| Deoxycholic acid | -0.011273 | -0.096324 | 0.92353 | 0.93618 |
| PC(O-16:0/20:3) | 0.0050072 | 0.042782 | 0.96599 | 0.96599 |

**Table S7:** Metabolite correlation analysis with phenylalanine. Correlations coefficients were calculated based on Pearson's correlation with *p* <0.05 indicating significance.

| Metabolite | Correlation | *t*-stat | *p*-value | FDR *p*-value |
| --- | --- | --- | --- | --- |
| Choline | 0.78285 | 10.75 | 1.07E-16 | 3.95E-15 |
| Kynurenine | 0.71661 | 8.7785 | 4.85E-13 | 1.20E-11 |
| Ornithine | 0.68024 | 7.929 | 1.91E-11 | 3.54E-10 |
| LysoPC(P-18:0) | -0.67262 | -7.7662 | 3.87E-11 | 5.73E-10 |
| Histidine | 0.6656 | 7.62 | 7.27E-11 | 8.20E-10 |
| Alanine | 0.66487 | 7.6051 | 7.76E-11 | 8.20E-10 |
| PC(14:0/18:0) | 0.6622 | 7.5506 | 9.81E-11 | 9.07E-10 |
| Methionine | 0.65379 | 7.3824 | 2.02E-10 | 1.66E-09 |
| Tyrosine | 0.64632 | 7.2367 | 3.78E-10 | 2.80E-09 |
| Proline | 0.64466 | 7.205 | 4.33E-10 | 2.92E-09 |
| LysoPC(14:0) | -0.62219 | -6.7904 | 2.54E-09 | 1.57E-08 |
| LysoPC(18:1) | -0.60125 | -6.4289 | 1.17E-08 | 6.66E-08 |
| PC(O-16:0/2:0).1 | -0.58175 | -6.111 | 4.41E-08 | 2.33E-07 |
| S1P | -0.57324 | -5.9774 | 7.66E-08 | 3.78E-07 |
| Betaine | 0.56287 | 5.8184 | 1.47E-07 | 6.80E-07 |
| PC(18:1/14:0) | 0.5494 | 5.6179 | 3.32E-07 | 1.44E-06 |
| Glycoursodeoxycholic acid | 0.54624 | 5.5717 | 4.00E-07 | 1.64E-06 |
| PC(O-16:0/2:0).2 | -0.53322 | -5.3853 | 8.42E-07 | 3.14E-06 |
| PC(P-18:0/20:4) | 0.5331 | 5.3837 | 8.48E-07 | 3.14E-06 |
| Carnitine | 0.51775 | 5.1707 | 1.96E-06 | 6.92E-06 |
| LysoPC(P-16:0) | -0.51274 | -5.1027 | 2.56E-06 | 8.62E-06 |
| SM(d18:1/18:1) | -0.50177 | -4.9562 | 4.52E-06 | 1.45E-05 |
| PC(18:3/18:3) | -0.49334 | -4.8458 | 6.89E-06 | 2.12E-05 |
| PC(18:1/18:1) | 0.49047 | 4.8086 | 7.94E-06 | 2.35E-05 |
| Glycocholic acid | 0.48672 | 4.7605 | 9.52E-06 | 2.71E-05 |
| Leucine | 0.48405 | 4.7263 | 1.08E-05 | 2.97E-05 |
| PE(16:0/18:1) | 0.47874 | 4.6589 | 1.40E-05 | 3.69E-05 |
| LysoPC(15:0) | -0.47104 | -4.5624 | 2.00E-05 | 5.10E-05 |
| PC(16:0/14:0) | 0.46886 | 4.5353 | 2.21E-05 | 5.45E-05 |
| PC(O-16:0/20:4) | 0.46517 | 4.4898 | 2.61E-05 | 6.24E-05 |
| LysoPC(16:0).1 | -0.4626 | -4.4581 | 2.93E-05 | 6.78E-05 |
| PC(O-18:1/2:0) | -0.45834 | -4.4061 | 3.55E-05 | 7.96E-05 |
| CAR(12:0) | -0.43876 | -4.1718 | 8.23E-05 | 0.00017911 |
| PC(16:1/16:1) | -0.42845 | -4.0513 | 0.00012567 | 0.00026569 |
| PS(18:0/20:4) | -0.42178 | -3.9745 | 0.00016403 | 0.00033718 |
| PC(P-18:0/22:6) | 0.40446 | 3.7786 | 0.00031948 | 0.00063896 |
| CAR(10:0) | -0.38385 | -3.5517 | 0.00067482 | 0.0013141 |
| LysoPC(24:0) | -0.38077 | -3.5184 | 0.00075141 | 0.0014258 |
| Gal ceramide (d18:1/24:1) | 0.37736 | 3.4816 | 0.00084552 | 0.0015642 |
| LysoPC(18:0) | -0.37017 | -3.4046 | 0.0010796 | 0.0019486 |
| PC(O-16:0/22:6) | -0.35908 | -3.2872 | 0.0015576 | 0.0027444 |
| PI(18:0/20:4) | -0.34916 | -3.1836 | 0.0021388 | 0.0036808 |
| LysoPC(16:0).2 | 0.33856 | 3.0742 | 0.0029679 | 0.0049914 |
| SMd(18:1/24:1) | 0.33167 | 3.0039 | 0.0036504 | 0.0060028 |
| CAR(18:1) | -0.32369 | -2.923 | 0.0046131 | 0.0074211 |
| SM(d18:1/18:0) | 0.31728 | 2.8585 | 0.0055434 | 0.008728 |
| CAR(16:0) | -0.3157 | -2.8428 | 0.005796 | 0.0089355 |
| PC(20:4/20:4) | -0.29113 | -2.6 | 0.011275 | 0.017028 |
| SMd(18:1/12:0) | -0.28137 | -2.5052 | 0.014468 | 0.021412 |
| LysoPE(18:0) | -0.27456 | -2.4396 | 0.017132 | 0.024858 |
| PC(16:0/22:6) | -0.25211 | -2.2259 | 0.029108 | 0.041423 |
| PE(O-18:1/20:4) | -0.24841 | -2.1911 | 0.031637 | 0.044173 |
| PC(18:0/22:6) | 0.23607 | 2.0757 | 0.041446 | 0.056796 |
| Tryptophan | 0.22615 | 1.9837 | 0.051055 | 0.068692 |
| Acetylcholine | 0.21979 | 1.9249 | 0.058133 | 0.076818 |
| PC(O-16:0/20:5) | -0.21409 | -1.8726 | 0.065131 | 0.084557 |
| Isoleucine | 0.21283 | 1.861 | 0.066768 | 0.085187 |
| PE(16:0/18:2) | 0.21107 | 1.845 | 0.069096 | 0.086662 |
| PC(16:0/18:2) | 0.20773 | 1.8145 | 0.073714 | 0.090914 |
| Lyso PAF C-16 | 0.20283 | 1.7698 | 0.080942 | 0.098192 |
| Lyso(PE18:1) | -0.19675 | -1.7145 | 0.090671 | 0.10822 |
| PC(20:1/20:1) | 0.19255 | 1.6765 | 0.097909 | 0.115 |
| TMAO | 0.18835 | 1.6386 | 0.10561 | 0.12211 |
| Acetylcarnitine | 0.17263 | 1.4975 | 0.13859 | 0.15777 |
| PC(18:0/18:0) | 0.16592 | 1.4376 | 0.15483 | 0.1736 |
| PC(18:0/18:1) | -0.1649 | -1.4285 | 0.15743 | 0.17387 |
| PC(14:0/14:0) | 0.10626 | 0.91305 | 0.36422 | 0.39636 |
| Cholesterol | 0.10285 | 0.88344 | 0.3799 | 0.40743 |
| PC(16:0/18:1) | -0.0815 | -0.69866 | 0.48699 | 0.51207 |
| PE(18:0/22:6) | 0.080695 | 0.69172 | 0.49131 | 0.51207 |
| PC(O-16:0/20:3) | 0.073132 | 0.62651 | 0.53293 | 0.54774 |
| Deoxycholic acid | 0.054473 | 0.46611 | 0.64253 | 0.65133 |
| PC(16:0/18:0) | -0.014654 | -0.12522 | 0.9007 | 0.9007 |

**Table S8:** Metabolite correlation analysis with methionine. Correlations coefficients were calculated based on Pearson's correlation with *p* <0.05 indicating significance.

| Metabolite | Correlation | *t*-stat | *p*-value | FDR *p*-value |
| --- | --- | --- | --- | --- |
| Betaine | 0.82558 | 12.5 | 8.11E-20 | 3.00E-18 |
| Proline | 0.74838 | 9.6404 | 1.18E-14 | 2.92E-13 |
| Tyrosine | 0.70885 | 8.5864 | 1.11E-12 | 2.06E-11 |
| SM(d18:1/18:1) | -0.69699 | -8.3046 | 3.77E-12 | 5.58E-11 |
| Glycoursodeoxycholic acid | 0.68654 | 8.0675 | 1.05E-11 | 1.30E-10 |
| LysoPC(P-18:0) | -0.65772 | -7.4603 | 1.45E-10 | 1.53E-09 |
| Phenylalanine | 0.65379 | 7.3824 | 2.02E-10 | 1.87E-09 |
| Histidine | 0.64291 | 7.1715 | 5.00E-10 | 4.11E-09 |
| PC(16:0/14:0) | 0.61806 | 6.7173 | 3.47E-09 | 2.57E-08 |
| PC(14:0/18:0) | 0.61513 | 6.6661 | 4.31E-09 | 2.90E-08 |
| Kynurenine | 0.596 | 6.3417 | 1.69E-08 | 1.04E-07 |
| LysoPC(16:0).1 | -0.58055 | -6.0919 | 4.77E-08 | 2.72E-07 |
| PC(18:3/18:3) | -0.55698 | -5.73 | 2.11E-07 | 1.11E-06 |
| LysoPC(16:0).2 | 0.55385 | 5.6834 | 2.54E-07 | 1.26E-06 |
| PC(18:1/14:0) | 0.53623 | 5.4278 | 7.11E-07 | 3.29E-06 |
| PC(O-16:0/20:4) | 0.53008 | 5.3412 | 1.00E-06 | 4.37E-06 |
| PC(16:0/22:6) | -0.52137 | -5.2202 | 1.62E-06 | 6.65E-06 |
| PC(O-16:0/2:0).1 | -0.51847 | -5.1805 | 1.89E-06 | 7.36E-06 |
| Glycocholic acid | 0.51191 | 5.0914 | 2.68E-06 | 9.90E-06 |
| PC(18:1/18:1) | 0.50407 | 4.9866 | 4.02E-06 | 1.42E-05 |
| S1P | -0.49904 | -4.9203 | 5.18E-06 | 1.74E-05 |
| Alanine | 0.47497 | 4.6115 | 1.67E-05 | 5.36E-05 |
| Ornithine | 0.47308 | 4.5879 | 1.82E-05 | 5.61E-05 |
| PC(16:0/18:1) | -0.46893 | -4.5362 | 2.20E-05 | 6.52E-05 |
| Choline | 0.46714 | 4.5141 | 2.39E-05 | 6.65E-05 |
| PC(P-18:0/20:4) | 0.46668 | 4.5084 | 2.44E-05 | 6.65E-05 |
| PI(18:0/20:4) | -0.466 | -4.4999 | 2.52E-05 | 6.65E-05 |
| PE(16:0/18:1) | 0.46447 | 4.4812 | 2.70E-05 | 6.88E-05 |
| Leucine | 0.43841 | 4.1677 | 8.35E-05 | 0.00020599 |
| PC(O-16:0/2:0).2 | -0.42314 | -3.9901 | 0.00015541 | 0.00037098 |
| LysoPC(14:0) | -0.41801 | -3.9315 | 0.0001902 | 0.00043984 |
| Acetylcholine | 0.41587 | 3.907 | 0.00020678 | 0.00046369 |
| PC(14:0/14:0) | 0.39262 | 3.6474 | 0.00049384 | 0.0010748 |
| CAR(10:0) | -0.36295 | -3.328 | 0.0013727 | 0.0029024 |
| TMAO | 0.34668 | 3.1579 | 0.0023114 | 0.0047511 |
| Tryptophan | 0.31885 | 2.8743 | 0.0053019 | 0.010394 |
| Isoleucine | 0.31861 | 2.8719 | 0.0053377 | 0.010394 |
| PE(O-18:1/20:4) | -0.30846 | -2.7706 | 0.0070917 | 0.013456 |
| Carnitine | 0.30317 | 2.7182 | 0.008194 | 0.015159 |
| PC(18:0/18:0) | 0.29153 | 2.604 | 0.011157 | 0.020137 |
| Gal ceramide (d18:1/24:1) | 0.29048 | 2.5937 | 0.011466 | 0.020202 |
| PC(O-18:1/2:0) | -0.28399 | -2.5306 | 0.013543 | 0.023306 |
| PC(P-18:0/22:6) | 0.27058 | 2.4015 | 0.018877 | 0.031135 |
| Cholesterol | 0.27046 | 2.4003 | 0.018934 | 0.031135 |
| Acetylcarnitine | 0.2416 | 2.1272 | 0.036779 | 0.059166 |
| PC(20:1/20:1) | 0.23464 | 2.0623 | 0.042736 | 0.067287 |
| PC(20:4/20:4) | -0.2282 | -2.0026 | 0.04894 | 0.075449 |
| LysoPE(18:0) | -0.22699 | -1.9913 | 0.050185 | 0.07579 |
| CAR(12:0) | -0.22528 | -1.9756 | 0.051983 | 0.076935 |
| PC(O-16:0/22:6) | -0.21598 | -1.89 | 0.062733 | 0.090437 |
| PC(O-16:0/20:5) | -0.21448 | -1.8762 | 0.064627 | 0.090437 |
| PC(O-16:0/20:3) | 0.21343 | 1.8666 | 0.065974 | 0.090437 |
| LysoPC(15:0) | -0.21342 | -1.8665 | 0.065994 | 0.090437 |
| PC(16:1/16:1) | -0.20659 | -1.804 | 0.075358 | 0.10139 |
| LysoPC(18:1) | -0.19303 | -1.6808 | 0.097067 | 0.12827 |
| CAR(18:1) | -0.18175 | -1.5792 | 0.11862 | 0.154 |
| PC(16:0/18:0) | 0.16337 | 1.4148 | 0.16138 | 0.20589 |
| PC(16:0/18:2) | 0.16152 | 1.3984 | 0.16622 | 0.20848 |
| PE(18:0/22:6) | -0.15741 | -1.3619 | 0.17743 | 0.21883 |
| PS(18:0/20:4) | -0.15335 | -1.3259 | 0.189 | 0.22927 |
| PC(18:0/22:6) | -0.14168 | -1.2228 | 0.22532 | 0.26893 |
| Lyso PAF C-16 | 0.12484 | 1.075 | 0.2859 | 0.33582 |
| Lyso(PE18:1) | 0.12195 | 1.0498 | 0.29727 | 0.33921 |
| LysoPC(P-16:0) | -0.12178 | -1.0483 | 0.29796 | 0.33921 |
| SMd(18:1/12:0) | 0.099904 | 0.85787 | 0.39377 | 0.4415 |
| PE(16:0/18:2) | 0.094416 | 0.81031 | 0.42039 | 0.46431 |
| LysoPC(24:0) | -0.088926 | -0.76281 | 0.44804 | 0.48757 |
| CAR(16:0) | -0.061345 | -0.52512 | 0.60109 | 0.64465 |
| SMd(18:1/24:1) | 0.057931 | 0.4958 | 0.62153 | 0.65704 |
| Deoxycholic acid | -0.040352 | -0.34505 | 0.73105 | 0.76194 |
| LysoPC(18:0) | -0.03866 | -0.33056 | 0.74192 | 0.76253 |
| SM(d18:1/18:0) | 0.031214 | 0.26682 | 0.79036 | 0.80118 |
| PC(18:0/18:1) | -0.0092433 | -0.078979 | 0.93727 | 0.93727 |

**Table S9:** Pathway analysis results. Statistical *p*-values are determined by enrichment analysis and adjusted for multiple testing. Pathway impact is determined by pathway topology analysis.  “Total” = total number of compounds in the pathway; “Hits” = matched number of metabolites from the current analysis; “Raw *p*-value” = original *p*-value calculated from the enrichment analysis; “ Holm adjust” = *p*-value adjusted by Holm-Bonferroni method; “FDR *p*-value” = *p*-value adjusted using the false discovery rate; “Impact” = pathway impact value calculated from pathway topology analysis.

| Pathway | Total | Expected | Hits | Raw  *p*-value | -log(p) | Holm adjust | FDR  *p*-value | Impact |
| --- | --- | --- | --- | --- | --- | --- | --- | --- |
| Beta Oxidation of Very Long Chain Fatty Acids | 13 | 0.63 | 2 | 1.28E-01 | 2.05E+00 | 1.00E+00 | 1.00E+00 | 0.05 |
| Betaine Metabolism | 18 | 0.87 | 2 | 2.16E-01 | 1.53E+00 | 1.00E+00 | 1.00E+00 | 0.14 |
| Oxidation of Branched Chain Fatty Acids | 22 | 1.07 | 2 | 2.90E-01 | 1.24E+00 | 1.00E+00 | 1.00E+00 | 0.00 |
| Phospholipid Biosynthesis | 25 | 1.21 | 2 | 3.45E-01 | 1.06E+00 | 1.00E+00 | 1.00E+00 | 0.09 |
| Phenylalanine and Tyrosine Metabolism | 25 | 1.21 | 2 | 3.45E-01 | 1.06E+00 | 1.00E+00 | 1.00E+00 | 0.22 |
| Beta-Alanine Metabolism | 26 | 1.26 | 2 | 3.63E-01 | 1.01E+00 | 1.00E+00 | 1.00E+00 | 0.00 |
| Phosphatidylethanolamine Biosynthesis | 13 | 0.63 | 1 | 4.79E-01 | 7.37E-01 | 1.00E+00 | 1.00E+00 | 0.00 |
| Catecholamine Biosynthesis | 14 | 0.68 | 1 | 5.04E-01 | 6.85E-01 | 1.00E+00 | 1.00E+00 | 0.00 |
| Histidine Metabolism | 35 | 1.70 | 2 | 5.16E-01 | 6.62E-01 | 1.00E+00 | 1.00E+00 | 0.24 |
| Sphingolipid Metabolism | 36 | 1.75 | 2 | 5.31E-01 | 6.62E-01 | 1.00E+00 | 1.00E+00 | 0.07 |
| Carnitine Synthesis | 16 | 0.78 | 1 | 5.52E-01 | 6.33E-01 | 1.00E+00 | 1.00E+00 | 0.00 |
| Methionine Metabolism | 39 | 1.89 | 2 | 5.76E-01 | 5.94E-01 | 1.00E+00 | 1.00E+00 | 0.04 |
| Fatty Acid Metabolism | 40 | 1.94 | 2 | 5.90E-01 | 5.52E-01 | 1.00E+00 | 1.00E+00 | 0.00 |
| Phosphatidylcholine Biosynthesis | 18 | 0.87 | 1 | 5.95E-01 | 5.28E-01 | 1.00E+00 | 1.00E+00 | 0.00 |
| Mitochondrial β-Oxidation of Long Chain Saturated Fatty Acids | 24 | 1.17 | 1 | 7.01E-01 | 3.55E-01 | 1.00E+00 | 1.00E+00 | 0.00 |
| Glycine and Serine Metabolism | 50 | 2.43 | 2 | 7.13E-01 | 3.38E-01 | 1.00E+00 | 1.00E+00 | 0.00 |
| Ammonia Recycling | 25 | 1.21 | 1 | 7.16E-01 | 3.34E-01 | 1.00E+00 | 1.00E+00 | 0.00 |
| Porphyrin Metabolism | 36 | 1.75 | 1 | 8.39E-01 | 1.76E-01 | 1.00E+00 | 1.00E+00 | 0.00 |
| Tyrosine Metabolism | 55 | 2.67 | 1 | 9.40E-01 | 6.17E-02 | 1.00E+00 | 1.00E+00 | 0.00 |
| Tryptophan Metabolism | 55 | 2.67 | 1 | 9.40E-01 | 6.17E-02 | 1.00E+00 | 1.00E+00 | 0.09 |
| Bile Acid Biosynthesis | 59 | 2.87 | 1 | 9.52E-01 | 4.97E-02 | 1.00E+00 | 1.00E+00 | 0.00 |
| Arachidonic Acid Metabolism | 65 | 3.16 | 1 | 9.65E-01 | 3.59E-02 | 1.00E+00 | 1.00E+00 | 0.00 |

**Table S10:** Subject characteristics by clinical presentation. Data are expressed as mean ± SD. Statistical difference between groups was calculated by one-way ANOVA, with *p*-value <0.05 indicating significance.

|  | Healthy Control  (n=15) | WD Pre-clinical  (n=12) | WD Neurologic  (n=22) | WD Hepatic  (n=26) | *p*-value |
| --- | --- | --- | --- | --- | --- |
| Male (%) | 5 (33.3%) | 5 (41.6%) | 13 (59.0%) | 12 (46.1%) | - |
| Female (%) | 10 (66.6%) | 7 (58.3%) | 9 (40.9%) | 14 (53.8) |  |
| Age (years) | 36.13 ± 9.20 | 31.25 ± 13.22 | 36.50 ± 11.47 | 33.08 ± 11.50 | 0.503 |
| BMI (Kg/m^2^) | 23.52 ± 3.88 | 25.56 ± 3.30 | 26.01 ± 6.30 | 25.31 ± 4.47 | 0.480 |

**Table S11:** List of internal standards used for quality control. RT, retention time; m/z, mass-to-charge ratio.

| **Metabolite name** | **Species** | **m/z** | **RT** |
| --- | --- | --- | --- |
| 1_15N2-L-Arginine iSTD | [2M+H]+ | 177.1122 | 9.15 |
| 1_CUDA iSTD | [M+H]+ | 341.2809 | 1.15 |
| 1_D3-1-Methylnicotinamide iSTD | [M-H]- | 141.0937 | 6.03 |
| 1_D3-Creatine iSTD | [M+H]+ | 135.0952 | 7.78 |
| 1_D3-Creatinine iSTD | [M+H]+ | 117.0846 | 4.88 |
| 1_D3-DL-Alanine iSTD | [M+H]+ | 93.0731 | 7.80 |
| 1_D3-DL-Aspartic acid iSTD | [M-H]- | 137.0626 | 8.89 |
| 1_D3-DL-Glutamic acid iSTD | [M+H]+ | 151.0785 | 8.45 |
| 1_D3-Histamine, N-methyl- iSTD | [M+H]+ | 129.1207 | 7.10 |
| 1_D3-L-Carnitine iSTD | [M+H]+ | 165.131 | 7.47 |
| 1_D5-L-Glutamine iSTD | [M+H]+ | 152.1069 | 8.29 |
| 1_D9-Betaine iSTD | [M+H-H2O]+ | 127.142 | 6.99 |
| 1_D9-Butyrobetaine iSTD | [M+H]+ | 155.174 | 7.45 |
| 1_D9-Caffeine iSTD | [M+H]+ | 204.1438 | 1.21 |
| 1_D9-Choline iSTD | [M+H]+ | 113.1627 | 5.10 |
| 1_D9-Crotonobetaine iSTD | [M+H]+ | 153.1582 | 7.53 |
| 1_D9-TMAO iSTD | [M+H]+ | 85.1316 | 5.39 |
| 1_Val-Tyr-Val iSTD | [M+H]+ | 380.2181 | 6.64 |

**Table S12:** List of primers for mouse gene expression.

| Gene | Gene name | Primer | Sequence 5' to 3' | Exon-exon overlap | % Primer efficiency |
| --- | --- | --- | --- | --- | --- |
| *Aldh7a1* | aldehyde dehydrogenase family 7, member A1 | F | CCTGGGACCCCAATATCCTC | Yes | 100.5 |
|  |  | R | ACCTTGCCCCCATAGACCAC | No |  |
| *Bhmt* | Betaine-homocysteine methyltransferase 2 | F | TTGGATTGGAACCCCGAGT | Yes | 104.0 |
|  |  | R | CCTGATGTGGTAGGGCTCAAA | No |  |
| *Bhmt2* | Betaine-homocysteine methyltransferase | F | GAAAGAGGGCCTCAGAGATGC | No | 101.5 |
|  |  | R | GGATACTCTGGGAGGTCCACAA | No |  |
| *Chdh* | Choline dehydrogenase | F | GAAGCCCCGACAGCTATTCC | No | 101.8 |
|  |  | R | ACTTCCAGACAACCCCAGCA | No |  |
| *Chpt1* | Choline phosphotransferase 1 | F | AAGCACCGGAACAGGTTCAA | Yes | 101.7 |
|  |  | R | CATGAAACAGCAGCAGCAAGA | No |  |
| *Gapdh* | glyceraldehyde-3-phosphate dehydrogenase | F | GAAGCTTGTCATCAACGGGAAG | No | 102.1 |
|  |  | R | TTTGATGTTAGTGGGGTCTCGC | No |  |
| *Mat1a* | methionine adenosyltransferase I, alpha | F | TCTGTCCCATACTCACCTCTTCAG | No | 98.4 |
|  |  | R | TGCCCTGAGGGTAGAAGGC | No |  |
| *Mat2a* | methionine adenosyltransferase II, alpha | F | CAGGAGACCAGGGTTTGATGTT | Yes | 95.8 |
|  |  | R | GCGTAACCAAGGCAATGTACC | No |  |
| *Mtr* | methionine synthase | F | CTGCAGATGTGGCCAGAAAAG | No | 98.9 |
|  |  | R | CAGCCACAAACCTCTTGACTCC | Yes |  |
| *Pcyt1a* | Phosphate cytidylyltransferase 1, choline, alpha isoform | F | GAAGAGCATCGACCTCATCCA | No | 101.3 |
|  |  | R | CTTCAGCATGTGCTTCAGTGC | Yes |  |
| *Pemt* | phosphatidylethanolamine N-methyltransferase | F | TACTTCCTGGGCCTTGCATTC | No | 100.1 |
|  |  | R | ACGCTGAAGGGAAATGTGGTC | No |  |

**Figure S3:** Features view showing distribution before and after normalization. Density plots represent all features. Boxplots display distribution for only 50 features due to limited space.


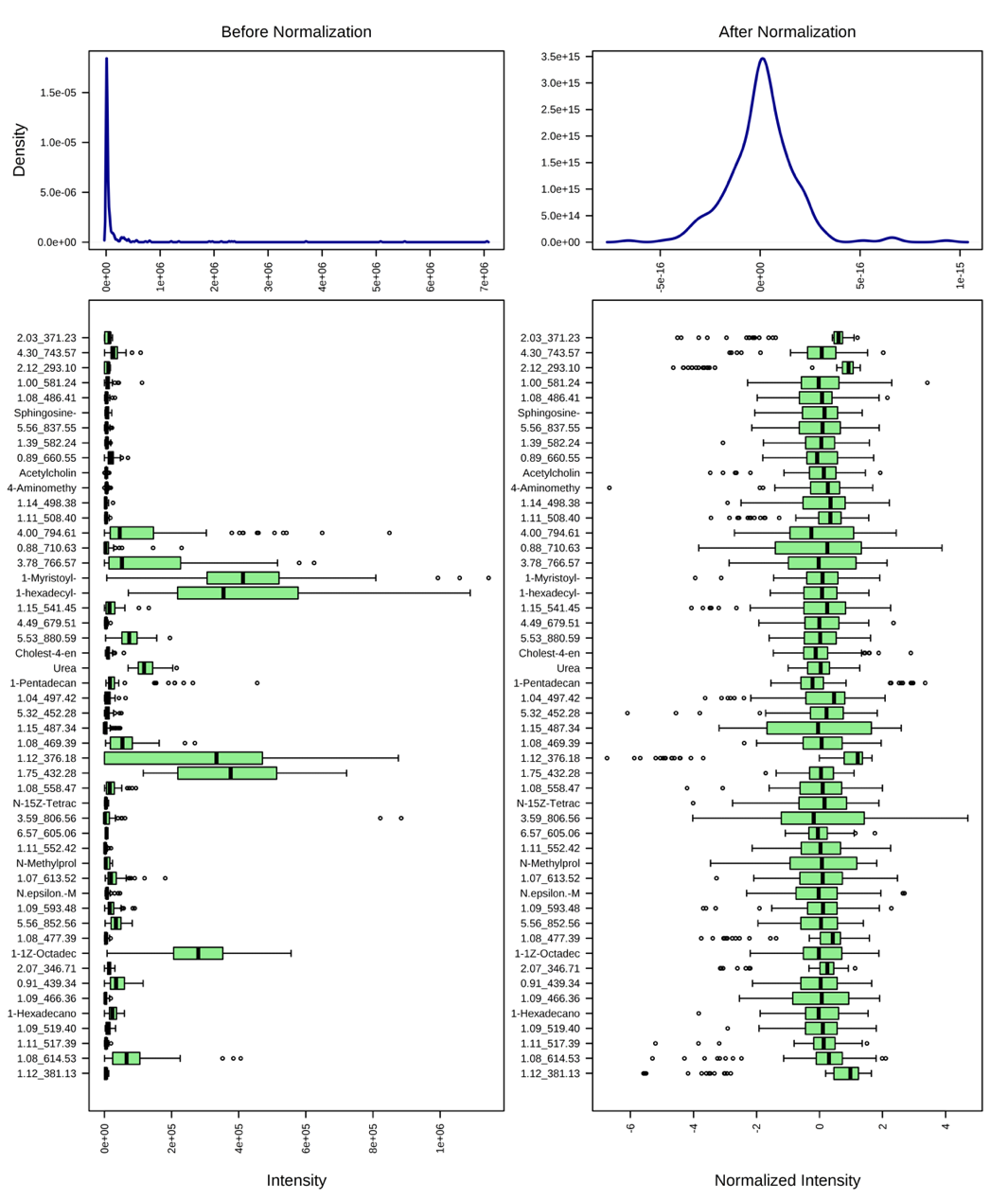

Supplement: Supplementary file 1 [file ijms-20-05937-s001.zip › Supplements/IJMS-Supplemental-Highlight.docx]
