# Supplementary material for: Dysregulated Choline, Methionine, and Aromatic Amino Acid Metabolism in Patients with Wilson Disease: Exploratory Metabolomic Profiling and Implications for Hepatic and Neurologic Phenotypes
Source: Int J Mol Sci. 2019 Nov 26;20(23):5937. doi: 10.3390/ijms20235937 (PMC6928853; doi:10.3390/ijms20235937)

Supplemental Material

Figure S1: Principal component analysis based on all detected metabolites. The first and second principal component scores plot show separation and clustering of WD and HC groups with some overlapping. The explained variances are shown in brackets.

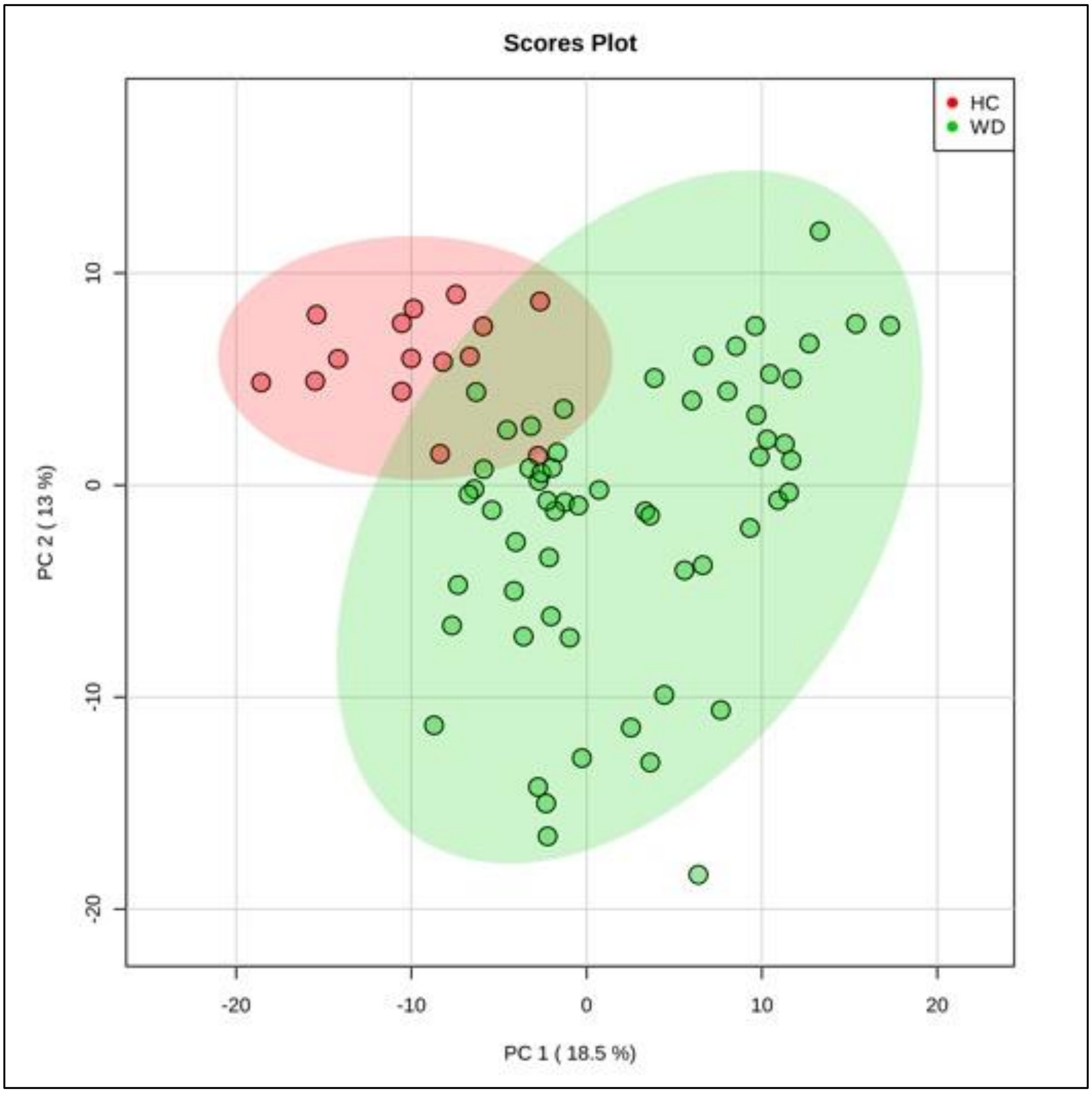

Figure S2: Volcano plot for WD compared to HC based on all detected metabolites. Important features selected with fold change (FC) threshold 1.2 and t-test (p) threshold 0.1. Both fold changes and *p*-values are log-transformed. The pink circles represent features above the threshold. The further the circle's position away from (0,0), the more significant the feature.

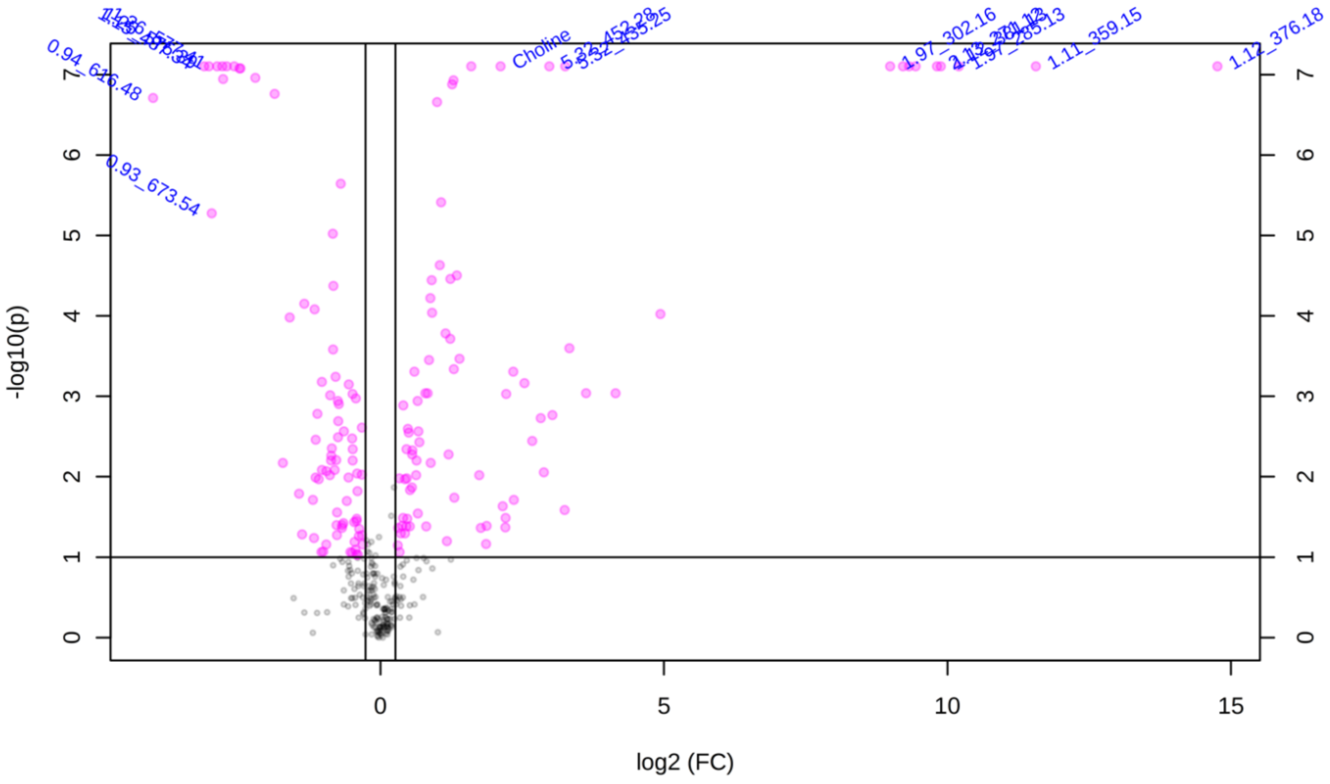

Table S1: Comprehensive list of differential metabolites comparing WD to HC. Significant metabolites were selected by volcano plot with fold change (FC) >1.2 and FDR-adjusted *p* <0.1 indicating significance.

Metabolite Name/Identifier	InChI Key	FC	log2 (FC)	FDR *p*-value	-log10 (p)	Species	m/z	RT
1.97_307.11	NA	594.48	9.2155	1.00E-73	72.998	[M+H]+	307.1147	1.97
1.97_285.13	NA	1176.9	10.201	7.06E-64	63.151	[M+H-H2O]+	285.1333	1.97
2.12_293.10	NA	640.54	9.3232	8.09E-64	63.092	[2M+H]+	293.0991	2.12
1.11_359.15	NA	3018.3	11.56	2.28E-59	58.643	[M+H]+	359.152	1.11
1.12_376.18	NA	27746	14.76	3.83E-58	57.417	[M+H]+	376.1759	1.12
2.13_271.12	NA	900.89	9.8152	2.70E-57	56.569	[2M+H]+	271.1173	2.13
2.13_288.14	NA	694.27	9.4394	8.44E-57	56.074	[M+H]+	288.1439	2.13
1.97_302.16	NA	508.34	8.9897	8.00E-56	55.097	[M+H]+	302.1595	1.97
1.12_381.13	NA	932.02	9.8642	2.78E-36	35.555	[M+Na]+	381.1296	1.12
5.32_435.25	NA	9.5686	3.2583	2.56E-20	19.591	[M-H]-	435.2497	5.32
5.31_104.11	NA	3.0336	1.601	1.87E-17	16.729	[M+H]+	104.1061	5.31
1.21_531.37	NA	0.15263	-2.7119	5.34E-16	15.272	[M+H]+	531.368	1.21
Choline	OEYIOHPDSNJKLS-UHFFFAOYSA-N	4.3424	2.1185	8.76E-16	15.058	[M+H]+	104.1066	5.12
5.32_452.28	NA	7.8835	2.9788	9.66E-15	14.015	[M-H]-	452.2765	5.32
0.94_616.48	NA	0.06208	-4.0097	7.96E-14	13.099	[M+H]+	616.4769	0.94
1.26_577.41	NA	0.13579	-2.8805	2.78E-13	12.557	[M+H]+	577.4071	1.26
1.25_575.39	NA	0.12284	-3.0252	4.11E-13	12.386	[M+H]+	575.3942	1.25
Phenylalanine	COLNVLDHVKWLRT-QMMMGPOBSA-N	2.4421	1.2881	2.79E-12	11.554	[M+H]+	166.0862	6.61
2-Hydroxy carbamazepine	NA	2.403	1.2649	3.19E-12	11.496	[M+H]+	253.0974	1.24
1.16_489.36	NA	0.27424	-1.8665	4.03E-12	11.395	[M+H]+	489.3574	1.16
6.61_149.06	NA	1.9985	0.99891	4.96E-11	10.305	[M-H]-	149.0589	6.61
0.93_672.54	NA	0.14607	-2.7752	6.42E-11	10.192	[M+H]+	672.5381	0.93
1.23_533.38	NA	0.18064	-2.4688	2.06E-10	9.6861	[M+H]+	533.3839	1.23
0.93_673.54	NA	0.12712	-2.9757	6.32E-10	9.1991	[M+H]+	673.5387	0.93
1.15_487.34	NA	0.11591	-3.109	8.29E-09	8.0815	[M-H]-	487.3427	1.15
1.26_555.40	NA	0.16726	-2.5799	6.00E-08	7.222	[M+NH4]+	555.3977	1.26
1.23_553.38	NA	0.14464	-2.7895	1.52E-07	6.8196	[M+H]+	553.3824	1.23
5.56_852.56	NA	2.0657	1.0467	2.47E-07	6.6072	[M+H]+	852.5609	5.56
PC(18:1/14:0)	KIVAJCJTVPWSRJ-OQHNRNOKSA-N	1.8408	0.88035	1.07E-06	5.9716	[M+H]+	732.5534	4.43
5.56_835.53	NA	1.8711	0.90387	1.07E-06	5.9716	[M+H]+	835.5327	5.56
1.21_511.37	NA	0.21659	-2.2069	1.24E-06	5.9057	[M+H]+	511.3718	1.21
5.15_544.34	NA	0.5586	-0.84012	1.43E-06	5.8433	[M+Na]+	544.3397	5.15
1.45_738.49	NA	0.6154	-0.7004	1.43E-06	5.8433	[M+NH4]+	738.4926	1.45
0.99_394.35	NA	30.647	4.9377	1.43E-06	5.8433	[M+NH4]+	394.3517	0.99

5.56_854.57	NA	2.5438	1.347	2.98E-06	5.5258	[M+NH4]+	854.5748	5.56
5.56_837.55	NA	2.352	1.2339	5.34E-06	5.2725	[M+H]+	837.5476	5.56
Ornithine	AHLPHDHHMVZTML-BYPYZUCNSA-N	2.3478	1.2313	9.17E-06	5.0379	[M+H]+	133.0961	9.27
PC(14:0/14:0)	CITHEXJVPOWHKC-UUWRZZSWSA-N	2.0995	1.0701	1.13E-05	4.9477	[M+H]+	678.5046	4.49
9.27_116.07	NA	2.2169	1.1485	3.22E-05	4.4922	[M+H]+	116.0696	9.27
LysoPC(P-18:0)	WBOMIOWRFSPZMC-AYICAFKVSA-N	0.57314	-0.80303	3.23E-05	4.4903	[M+H]+	508.3748	5.22
1.18_509.36	NA	0.17857	-2.4855	5.75E-05	4.2404	[M+H]+	509.3562	1.18
3.07_400.34	NA	2.4515	1.2937	7.14E-05	4.1462	[M+H]+	400.3414	3.07
0.99_377.32	NA	17.709	4.1464	7.20E-05	4.1424	[M+H]+	377.3205	0.99
0.92_600.50	NA	0.60122	-0.73405	8.27E-05	4.0824	[M+H]+	600.4984	0.92
0.90_610.54	NA	1.5746	0.65502	8.51E-05	4.0699	[M+H]+	610.5391	0.90
Histidine	HNDVDQJCIGZPNO-YFKPBYRVSA-N	1.7805	0.83231	9.31E-05	4.0313	[M+H]+	156.0762	9.07
Alanine	QNAYBMKLOCPYGJ-REOHCLBHSA-N	1.3987	0.4841	0.00013065	3.8839	[M+H]+	90.0542	7.79
1.08_541.45	NA	0.32984	-1.6002	0.00014455	3.84	[M+H]+	541.4482	1.08
0.93_364.27	NA	5.815	2.5398	0.00014455	3.84	[M+H]+	364.2681	0.93
0.90_611.54	NA	1.5898	0.66887	0.00014666	3.8337	[M+H]+	611.5373	0.90
2.87_426.36	NA	4.6498	2.2172	0.00018085	3.7427	[M+H]+	426.3571	2.87
4.30_732.55	NA	1.8801	0.91079	0.00028574	3.544	[M+H]+	732.5535	4.30
5.13_500.30	NA	8.1828	3.0326	0.00028574	3.544	[M+H]+	500.303	5.13
1.00_551.55	NA	10.073	3.3324	0.00028574	3.544	[M+H]+	551.5454	1.00
PS(18:0/20:4)	SVOUGFFDROZBJI-DNALCEECSA-N	0.39422	-1.3429	0.00031406	3.503	[M+H]+	812.5433	5.40
LysoPC(15:0)	RJZVWDTYEWCUAR-JOCHJYFZSA-N	0.30327	-1.7213	0.00034956	3.4565	[M+H]+ _[M+Na]+	482.3215 _504.3075	5.27
1.09_466.36	NA	0.4885	-1.0336	0.00034956	3.4565	[M+H]+	466.3605	1.09
LysoPC(14:0)	VXUOFDJKYGDUJI-OAQYLSRUSA-N	0.57791	-0.79108	0.00034956	3.4565	[M+H]+	468.3086	5.29
S1P	DUYSYHSSBDVJSM-KRWOKUGFSA-N	0.67908	-0.55836	0.00034956	3.4565	[M+H]+	380.2552	5.96
Kynurenine	YGPSJZOEDVAXAB-QMMMGPOBSA-N	1.5142	0.59854	0.00050566	3.2961	[M+H]+	209.0914	6.62
5.16_482.32	NA	0.44705	-1.1615	0.00075461	3.1223	[M+H]+	482.3218	5.16
LysoPC(24:0)	SKJMUADLQLZAGH-WJOKGBTCSA-N	0.74014	-0.43413	0.0010653	2.9725	[M+H]+	608.4634	5.03
1.00_549.53	NA	7.3728	2.8822	0.0011621	2.9348	[2M+H]+	549.5341	1.00
Tyrosine	OUYCCCASQSFEME-QMMMGPOBSA-N	1.7307	0.79133	0.0012417	2.906	[M+H]+	182.0802	7.39
1.01_507.49	NA	7.0977	2.8274	0.0012417	2.906	[2M+H]+	507.4856	1.01
0.99_591.58	NA	0.55183	-0.85771	0.0013086	2.8832	[M+H]+	591.5819	0.99
PC(18:3/18:3)	XXKFQTJOJZELMD-JICBSJGISA-N	0.59446	-0.75035	0.0014346	2.8433	[M+H]+	778.5374	4.18
1.14_481.35	NA	0.45316	-1.1419	0.0019149	2.7178	[M+H]+	481.3514	1.14
PC(P-18:0/20:4)	FHHVIBPVBBRLOR-IYACIECVSA-N	1.6094	0.68651	0.0020141	2.6959	[M+H]+	794.6058	4.30
0.99_536.50	NA	2.3002	1.2017	0.0020916	2.6795	[M+H]+	536.5023	0.99
LysoPC(18:1)	YAMUFBLWGFFICM-PTGWMXDISA-N	0.79667	-0.32795	0.0022638	2.6452	[M+H]+	522.3559	5.17
0.93_347.24	NA	5.0716	2.3424	0.0023539	2.6282	[M+H-H2O]+	347.2419	0.93

Proline	ONIBWKKTOPOVIA-BYPYZUCNSA-N	1.4771	0.56272	0.0025545	2.5927	[M+H]+	116.0696	7.44
4.58_432.31	NA	6.3961	2.6772	0.0026054	2.5841	[M+H-H2O]+	432.3093	4.58
0.89_612.55	NA	1.8109	0.85668	0.0028009	2.5527	[M+H]+	612.5537	0.89
1.18_490.37	NA	0.70748	-0.49925	0.0032195	2.4922	[M+H]+	490.3735	1.18
PC(16:1/16:1)	GPWHCUUIQMGELX-VHQDNGOZSA-N	0.75181	-0.41156	0.0032737	2.485	[M+H]+	730.5372	4.43
1.01_509.50	NA	4.4574	2.1562	0.0032737	2.485	[M+H]+	509.5023	1.01
PE(16:0/18:1)	FHQVHHIBKUMWTI-OTMQOFQLSA-N	1.4353	0.52135	0.0041038	2.3868	[M+H]+	718.5372	4.58
N.epsilon.-Methyl-L-lysine	PQNASZJZHFPQLE-UHFFFAOYSA-N	1.8484	0.88624	0.0041038	2.3868	[M+H]+	161.1275	8.99
1.00_581.24	NA	0.51665	-0.95274	0.0042745	2.3691	[M+K]+	581.2378	1.00
Lyso PAF C-16	VLBPIWYTPAXCFJ-UHFFFAOYSA-N	1.3151	0.39521	0.0043146	2.3651	[M+H]+	482.3582	5.27
5.14_517.33	NA	12.35	3.6265	0.0044294	2.3537	[M+H]+	517.3291	5.14
LysoPC(16:0)	NA	1.3057	0.38488	0.0047451	2.3238	[M+H]+	496.3405	5.22
5.06_522.36	NA	0.71245	-0.48914	0.0051345	2.2895	[M+H]+	522.3557	5.06
1.08_558.47	NA	0.54813	-0.86742	0.0053599	2.2708	[M+NH4]+	558.4708	1.08
0.99_593.60	NA	0.58237	-0.77999	0.0056395	2.2488	[M+H]+	593.5972	0.99
1.12_535.40	NA	0.54669	-0.87121	0.0063809	2.1951	[M+NH4]+	535.3992	1.12
4.49_726.54	NA	0.67688	-0.56302	0.0071769	2.1441	[M+H]+	726.5413	4.49
Methionine	FFEARJCKVFRZRR-BYPYZUCNSA-N	2.628	1.394	0.0076592	2.1158	[M+H]+ _[M+N-NH3]+	150.0575 _133.0308	7.16
1.09_484.40	NA	0.63975	-0.64443	0.0077741	2.1093	[M+NH4]+	484.3964	1.09
1.09_467.37	NA	0.597	-0.74419	0.0089033	2.0504	[M+H]+	467.3722	1.09
CAR(12:0)	FUJLYHJROOYKRA-QGZVFWFLSA-N	0.45264	-1.1435	0.0091634	2.0379	[M+H]+	344.2791	3.69
PC(O-16:0/22:6)	QQQQNYAHSSIZBU-HIQXTUQZSA-N	0.47007	-1.089	0.0096539	2.0153	[M+H]+	792.5906	3.85
4.99_508.38	NA	0.75516	-0.40514	0.0096539	2.0153	[M+H]+	508.375	4.99
Glycocholic acid	RFDAIACWWDREDC-FRVQLJSFSA-N	3.3463	1.7425	0.0096539	2.0153	[M+H]+ _[M+NH4]+	483.342 _466.3154	5.89
3.95_752.56	NA	0.43798	-1.1911	0.0097492	2.011	[M+H]+	752.5594	3.95
PC(16:0/14:0)	UIXXHROAQSBBOV-PSXMRANNSA-N	1.4733	0.55908	0.0099315	2.003	[M+H]+	706.5375	4.46
1.07_590.48	NA	0.36984	-1.435	0.009957	2.0019	[M+H]+	590.4756	1.07
5.51_876.56	NA	1.3788	0.46342	0.010174	1.9925	[M+H]+	876.5611	5.51
3.86_834.60	NA	0.53889	-0.89193	0.01042	1.9821	[M+H]+	834.6032	3.86
5.51_859.53	NA	1.3488	0.43169	0.01042	1.9821	[M+H]+	859.5334	5.51
Hippuric acid	QIAFMBKCNZACKA-UHFFFAOYSA-N	0.4888	-1.0327	0.011142	1.953	[M+H]+	180.0645	3.38
Carnitine	PHIQHXFUZVPYII-ZCFIWIBFSA-N	1.3187	0.39913	0.011368	1.9443	[M+H]+ _[M+Na]+	162.1127 _184.0937	7.48
1.12_530.44	NA	0.54093	-0.8865	0.011789	1.9285	[M+H]+	530.4398	1.12
0.90_855.74	NA	1.4127	0.49848	0.011789	1.9285	[M+H]+	855.7403	0.90
4.34_706.54	NA	1.5463	0.62878	0.011789	1.9285	[M+H]+	706.5372	4.34
4.09_806.57	NA	0.46259	-1.1122	0.012115	1.9167	[M+H]+	806.5707	4.09
4.59_450.32	NA	5.1065	2.3523	0.012196	1.9138	[M+H]+	450.3201	4.59
5-Aminovaleric acid	JJMDCOVWQOJGCB-UHFFFAOYSA-N	2.4665	1.3025	0.012615	1.8991	[M+H-H2O]+	100.0752	1.50

Betaine	NA	1.3747	0.45909	0.012706	1.896	[M+H]+	118.0863	6.98
1.00_561.53	NA	0.79599	-0.32917	0.013599	1.8665	[M+H]+	561.535	1.00
1.19_495.33	NA	0.7114	-0.49127	0.01454	1.8374	[M+K]+	495.3284	1.19
1.14_498.38	NA	0.57136	-0.80754	0.015377	1.8131	[M+NH4]+	498.3779	1.14
4.58_467.35	NA	4.6179	2.2072	0.015862	1.7996	[M+NH4]+	467.3469	4.58
SM(d18:1/12:0)	HZCLJRFPXMKWHR-FEBLJDHQSA-N	0.74823	-0.41844	0.01686	1.7732	[M+H]+	647.5109	4.99
7-a-OH-3-oxo-Cholest-4-en-26-oic acid	SATGKQGFUDXGAX-SLTBQWEQSA-N	1.5544	0.63632	0.016956	1.7707	[M+H]+	431.3148	1.21
1.01_533.50	NA	2.2536	1.1722	0.017936	1.7463	[M+H]+	533.5018	1.01
Glycoursodeoxycholic acid	GHCZAUBVMUEKKP-TVQURETASA-N	3.4105	1.77	0.017936	1.7463	[M+H-H2O]+	432.3095	4.71
4.12_808.58	NA	0.59533	-0.74825	0.019255	1.7155	[M+NH4]+	808.5849	4.12
4.71_467.35	NA	4.6032	2.2026	0.019665	1.7063	[M+NH4]+	467.3469	4.71
3.59_806.56	NA	9.5029	3.2484	0.021804	1.6615	[M+H]+	806.5649	3.59
1.10_610.50	NA	0.6264	-0.67485	0.021848	1.6606	[M+NH4]+	610.5033	1.10
1.09_611.50	NA	0.62514	-0.67776	0.023239	1.6338	[M+H]+	611.5043	1.09
3.68_752.56	NA	1.4323	0.51834	0.024429	1.6121	[M+H]+	752.5611	3.68
5.26_459.25	NA	1.2641	0.33816	0.024866	1.6044	[M+Na]+	459.2493	5.26
1.11_465.36	NA	0.56038	-0.83553	0.025615	1.5915	[M+K]+	465.3566	1.11
5.53_880.59	NA	1.3875	0.47249	0.025615	1.5915	[M+H]+	880.5926	5.53
4.33_704.52	NA	1.4312	0.51723	0.026407	1.5783	[M-H]-	704.5217	4.33
PC(P-18:0/22:6)	TXHZYNSTTCIWMJ-RXSQUPBGSA-N	1.5795	0.65944	0.030166	1.5205	[M+H]+	818.605	4.28
PC(18:0/22:6)	FAUYAENFVCNTAL-PFFNLMTBSA-N	1.4693	0.55516	0.033211	1.4787	[M+H]+	834.601	4.31
CAR(16:0)	XOMRRQXKHMYMOC-OAQYLSRUSA-N	0.58488	-0.77378	0.035685	1.4475	[M+H]+	400.3417	3.14
5.53_863.56	NA	1.3499	0.43288	0.037328	1.428	[M+H]+	863.5644	5.53
4.71_450.32	NA	3.6375	1.863	0.037577	1.4251	[M+H]+	450.3206	4.71
1.09_595.49	NA	0.58852	-0.76483	0.039665	1.4016	[M+H]+	595.494	1.09
1.13_463.34	NA	0.66275	-0.59347	0.040435	1.3932	[M+NH4]+	463.3411	1.13
LysoPC(P-16:0)	HTZINLFNXLXRBC-CQLBIITFSA-N	0.77154	-0.37418	0.040435	1.3932	[M+H]+	480.3446	5.05
5.26_476.28	NA	1.2362	0.30592	0.040435	1.3932	[M+NH4]+	476.2762	5.26
Leucine	ROHFNLRQFUQHCH-YFKPBYRVSA-N	1.2424	0.31312	0.040965	1.3876	[M+H]+	132.1013	6.76
SM(d18:1/18:0)	LKQLRGMMMAHREN-YJFXYUILSA-N	3.6618	1.8725	0.042682	1.3698	[M+H]+ _[M+Na]+	731.6044 _753.5881	4.92
3.89_752.56	NA	0.49714	-1.0083	0.044681	1.3499	[M+H]+	752.561	3.89
1.08_469.39	NA	0.67654	-0.56375	0.044817	1.3486	[M-H+2Li]+	469.3884	1.08
4.30_792.59	NA	1.3796	0.46428	0.044817	1.3486	[M+H]+	792.59	4.30
PC(O-16:0/20:4)	NA	1.2813	0.35766	0.048851	1.3111	[M+H]+	768.5897	4.33
CAR(10:0)	NA	0.63626	-0.65231	0.051466	1.2885	[M+H]+	316.2478	4.11
0.91_565.40	NA	0.74674	-0.42133	0.051466	1.2885	[M+H]+	565.4021	0.91
Trigonelline	WWNNZCOKKKDOPX-UHFFFAOYSA-N	0.44383	-1.1719	0.054675	1.2622	[M+H]+	138.0544	7.20
3-Methylhistidine	JDHILDINMRGULE-LURJTMIESA-N	1.7494	0.80683	0.055983	1.2519	[M+H]+	170.0917	9.10

1.06_596.50	NA	0.6278	-0.67162	0.057462	1.2406	[M+NH4]+	596.5042	1.06
Palmitoleoyl ethanolamide	WFRLANWAASSSFV-FPLPWBNLSA-N	1.3027	0.38155	0.057462	1.2406	[M+H]+	298.274	1.02
1.09_593.48	NA	0.69228	-0.53056	0.065682	1.1826	[M+H]+	593.478	1.09
4.00_794.61	NA	0.51541	-0.9562	0.066688	1.176	[M+H]+	794.6068	4.00
5.27_526.26	NA	0.38374	-1.3818	0.069315	1.1592	[M+H]+	526.2637	5.27
4.35_780.55	NA	0.80565	-0.31177	0.070296	1.1531	[M+K]+	780.5539	4.35
Acetylcarnitine	RDHQFKQIGNGIED-MRVPVSSYSA-O	1.3167	0.39688	0.074738	1.1265	[M+H]+	204.1234	6.95
1.08_597.51	NA	0.7075	-0.49919	0.076609	1.1157	[M+H]+	597.5082	1.08
5.20_468.31	NA	0.71194	-0.49016	0.081911	1.0867	[M+H]+	468.3072	5.20
1.09_612.52	NA	0.68312	-0.54979	0.089909	1.0462	[M+NH4]+	612.5181	1.09

Table S2: Significant annotated metabolites comparing WD to HC. Metabolites were selected by volcano plot with fold change (FC) >1.2 and FDR-adjusted *p* <0.1 indicating significance.

Metabolite Name/Identifier	InChI Key	FC	log2 (FC)	FDR *p*-value	-log10 (p)	Species	m/z	RT
Choline	OEYIOHPDSNJKLS-UHFFFAOYSA-N	4.0012	2.0004	1.39E-16	15.857	[M+H]+	104.1066	5.12
Phenylalanine	COLNVLDHVKWLRT-QMMMGPOBSA-N	2.2828	1.1908	8.69E-11	10.061	[M+H]+	166.0862	6.61
2-Hydroxyphenethylamine	ULSIYEODSMZIPX-UHFFFAOYSA-N	2.2469	1.1679	1.27E-10	9.895	[M+H-H2O]+	120.0804	6.61
PS(18:0/20:4)	SVOUGFFDROZBJI-DNALCEECSA-N	0.37007	-1.4341	1.75E-09	8.7569	[M+H]+	812.5433	5.4
S1P	DUYSYHSSBDVJSM-KRWOKUGFSA-N	0.63312	-0.65946	9.11E-09	8.0405	[M+H]+	380.2552	5.96
LysoPC(24:0)	SKJMUADLQLZAGH-WJOKGBTCSA-N	0.69909	-0.51645	0.00000118	5.9283	[M+H]+	608.4634	5.03
LysoPC(P-18:0)	WBOMIOWRFSPZMC-AYICAFKVSA-N	0.53166	-0.91143	0.00000211	5.6766	[M+H]+	508.3748	5.22
LysoPC(14:0)	VXUOFDJKYGDUJI-OAQYLSRUSA-N	0.54089	-0.88658	0.0000405	4.3922	[M+H]+	468.3086	5.29
Methionine	FFEARJCKVFRZRR-BYPYZUCNSA-N	2.4829	1.312	0.0000824	4.084	[M+H]+ _[M+N-NH3]+	150.0575 _133.030	7.16
PC(18:3/18:3)	XXKFQTJOJZELMD-JICBSJGISA-N	0.55151	-0.85853	0.0001062	3.9738	[M+H]+	778.5374	4.18
Hippuric acid	QIAFMBKCNZACKA-UHFFFAOYSA-N	0.46299	-1.111	0.0001276	3.8941	[M+H]+	180.0645	3.38
PC(18:1/14:0)	KIVAJCJTVPWSRJ-OQHNRNOKSA-N	1.7027	0.76784	0.0004403	3.3562	[M+H]+	732.5534	4.43
SM(d18:1/18:1)	NBEADXWAAWCCDG-QDDWGVBQSA-N	0.79652	-0.32822	0.000517	3.2866	[M+H]+	729.5913	4.93
LysoPC(18:1)	YAMUFBLWGFFICM-PTGWMXDISA-N	0.74449	-0.42567	0.0007722	3.1123	[M+H]+	522.3559	5.17
Kynurenine	YGPSJZOEDVAXAB-QMMMGPOBSA-N	1.4163	0.50211	0.0012296	2.9103	[M+H]+	209.0914	6.62
Tyrosine	OUYCCCASQSFEME-QMMMGPOBSA-N	1.6318	0.70646	0.001598	2.7964	[M+H]+	182.0802	7.39
PC(14:0/18:0)	TYAQXZHDAGZOEO-KXQOOQHDSA-N	1.9597	0.97061	0.0016193	2.7907	[M+H]+	734.5685	4.45
Betaine	KWIUHFFTVRNATP-UHFFFAOYSA-N	1.2811	0.35743	0.001999	2.6992	[M+H]+	118.0863	6.98
SM(d18:1/12:0)	HZCLJRFPXMKWHR-FEBLJDHQSA-N	0.70579	-0.5027	0.0020034	2.6982	[M+H]+	647.5109	4.99
Histidine	HNDVDQJCIGZPNO-YFKPBYRVSA-N	1.6583	0.72974	0.0035973	2.444	[M+H]+	156.0762	9.07
PC(O-16:0/22:6)	QQQQNYAHSSIZBU-HIQXTUQZSA-N	0.45367	-1.1403	0.0041881	2.378	[M+H]+	792.5906	3.85
PC(P-18:0/20:4)	FHHVIBPVBBRLOR-IYACIECVSA-N	1.4915	0.57678	0.0056143	2.2507	[M+H]+	794.6058	4.3
7-a-OH-3-oxo-Cholest-4-en-26-oic acid	SATGKQGFUDXGAX-SLTBQWEQSA-N	1.4507	0.53671	0.0062521	2.204	[M+H]+	431.3148	1.21
PC(16:1/16:1)	GPWHCUUIQMGELX-VHQDNGOZSA-N	0.69923	-0.51616	0.0075823	2.1202	[M+H]+	730.5372	4.43
CAR(12:0)	FUJLYHJROOYKRA-QGZVFWFLSA-N	0.42552	-1.2327	0.0078147	2.1071	[M+H]+	344.2791	3.69
Ornithine	AHLPHDHHMVZTML-BYPYZUCNSA-N	2.179	1.1237	0.012353	1.9082	[M+H]+	133.0961	9.27
4-Aminomethylcyclo hexane carboxylic acid	GYDJEQRTZSCIOI-LJGSYFOKSA-N	0.76913	-0.3787	0.015054	1.8223	[M-H]-	158.1166	6.62
PHE-PHE	GKZIWHRNKRBEOH-UHFFFAOYSA-N	0.64583	-0.63078	0.015136	1.82	[M+H]+	313.1546	5.57
LysoPC(15:0)	RJZVWDTYEWCUAR-JOCHJYFZSA-N	0.28009	-1.8361	0.015555	1.8081	[M+H]+ _[M+Na]+	482.3215 _504.307	5.27
Proline	ONIBWKKTOPOVIA-BYPYZUCNSA-N	1.3805	0.46521	0.015555	1.8081	[M+H]+	116.0696	7.44
N-Methylproline	CWLQUGTUXBXTLF-YFKPBYRVSA-N	1.7086	0.77284	0.020158	1.6956	[M+H]+	130.0856	6.87
PC(P-18:0/22:6)	TXHZYNSTTCIWMJ-RXSQUPBGSA-N	1.4507	0.53671	0.020538	1.6874	[M+H]+	818.605	4.28
Alanine	QNAYBMKLOCPYGJ-REOHCLBHSA-N	1.3144	0.39441	0.020763	1.6827	[M+H]+	90.0542	7.79
LysoPC(16:0).2	ASWBNKHCZGQVJV-HSZRJFAPSA-N	1.2194	0.28614	0.020763	1.6827	[M+H]+	518.3223	5.24

SM(d18:1/18:0)	LKQLRGMMMAHREN-YJFXYUILSA-N	3.3044	1.7244	0.020763	1.6827	[M+H]+ _[M+Na]+	731.6044 _753.588	4.92
PC(16:0/14:0)	UIXXHROAQSBBOV-PSXMRANNSA-N	1.372	0.45623	0.020763	1.6827	[M+H]+	706.5375	4.46
PC(18:0/22:6)	FAUYAENFVCNTAL-PFFNLMTBSA-N	1.3482	0.43104	0.021426	1.6691	[M+H]+	834.601	4.31
LysoPC(P-16:0)	HTZINLFNXLXRBC-CQLBIITFSA-N	0.72122	-0.4715	0.025922	1.5863	[M+H]+	480.3446	5.05
CAR(16:0)	XOMRRQXKHMYMOC-OAQYLSRUSA-N	0.55595	-0.84697	0.025922	1.5863	[M+H]+	400.3417	3.14
5-Aminopentanoic acid	JJMDCOVWQOJGCB-UHFFFAOYSA-N	2.2459	1.1673	0.025922	1.5863	[M+H-H2O]+	100.0752	1.5
Glycocholic acid	RFDAIACWWDREDC-FRVQLJSFSA-N	3.2332	1.6929	0.027337	1.5633	[M+H]+ _[M+NH4]+	483.342 _466.315	5.89
Glycoursodeoxycholic acid	GHCZAUBVMUEKKP-TVQURETASA-N	3.3015	1.7231	0.027337	1.5633	[M+H-H2O]+	432.3095	4.71
Trigonelline	WWNNZCOKKKDOPX-UHFFFAOYSA-N	0.43348	-1.206	0.027337	1.5633	[M+H]+	138.0544	7.2
CAR(10:0)	LZOSYCMHQXPBFU-OAHLLOKOSA-N	0.59019	-0.76075	0.027337	1.5633	[M+H]+	316.2478	4.11
3-Methylhistidine	JDHILDINMRGULE-LURJTMIESA-N	1.6305	0.7053	0.048372	1.3154	[M+H]+	170.0917	9.1
LysoPE(18:1)	PYVRVRFVLRNJLY-MZMPXXGTSA-N	0.83298	-0.26365	0.048552	1.3138	[M+H]+	480.308	5.44
Carnitine	PHIQHXFUZVPYII-ZCFIWIBFSA-N	1.2213	0.28843	0.053572	1.2711	[M+H]+ _[M+Na]+	162.1127 _184.093	7.48
Palmitoleoyl Ethanolamide	WFRLANWAASSSFV-FPLPWBNLSA-N	1.2205	0.28748	0.082333	1.0844	[M+H]+	298.274	1.02

Table S3: Significant annotated metabolites comparing WD pre-clinical to HC. Metabolites were selected by volcano plot with fold change (FC) >1.2 and FDR-adjusted *p* <0.1 indicating significance.

Metabolite Name/Identifier	InChI Key	FC	log2 (FC)	FDR *p*-value	-log10 (p)	Species	m/z	RT
Phenylalanine	COLNVLDHVKWLRT-QMMMGPOBSA-N	2.1545	1.1073	1.35E-08	7.8688	[M+H]+	166.0862	6.61
2-Hydroxy phenethylamine	ULSIYEODSMZIPX-UHFFFAOYSA-N	2.1174	1.0823	1.35E-08	7.8688	[M+H-H2O]+	120.0804	6.61
Choline	OEYIOHPDSNJKLS-UHFFFAOYSA-N	3.3713	1.7533	0.0001291 6	3.8889	[M+H]+	104.1066	5.12
LysoPC(14:0)	VXUOFDJKYGDUJI-OAQYLSRUSA-N	0.48547	-1.0425	0.0005243	3.2804	[M+H]+	468.3086	5.29
LysoPC(18:1)	YAMUFBLWGFFICM-PTGWMXDISA-N	0.72554	-0.46288	0.0026638	2.5745	[M+H]+	522.3559	5.17
LysoPC(24:0)	SKJMUADLQLZAGH-WJOKGBTCSA-N	0.63566	-0.65366	0.0038754	2.4117	[M+H]+	608.4634	5.03
S1P	DUYSYHSSBDVJSM-KRWOKUGFSA-N	0.66126	-0.59671	0.0058796	2.2306	[M+H]+	380.2552	5.96
LysoPC(P-18:0)	WBOMIOWRFSPZMC-AYICAFKVSA-N	0.61776	-0.69488	0.0072996	2.1367	[M+H]+	482.3235	5.42
SM(d18:1/12:0)	HZCLJRFPXMKWHR-FEBLJDHQSA-N	0.61614	-0.69868	0.0084358	2.0739	[M+H]+	647.5109	4.99
PC(16:1/16:1)	GPWHCUUIQMGELX-VHQDNGOZSA-N	0.66569	-0.58708	0.01034	1.9855	[M+H]+	730.5372	4.43
PC(18:1/14:0)	KIVAJCJTVPWSRJ-OQHNRNOKSA-N	1.5029	0.58776	0.013734	1.8622	[M+H]+	732.5534	4.43
PC(18:3/18:3)	XXKFQTJOJZELMD-JICBSJGISA-N	0.61953	-0.69075	0.013734	1.8622	[M+H]+	778.5374	4.18
LysoPC(P-16:0)	HTZINLFNXLXRBC-CQLBIITFSA-N	0.5871	-0.76832	0.013734	1.8622	[M+H]+	508.3748	5.22
PS(18:0/20:4)	SVOUGFFDROZBJI-DNALCEECSA-N	0.37882	-1.4004	0.013734	1.8622	[M+H]+	812.5433	5.4
Hippuric acid	QIAFMBKCNZACKA-UHFFFAOYSA-N	0.26282	-1.9279	0.013734	1.8622	[M+H]+	180.0645	3.38
PC(14:0/18:0)	TYAQXZHDAGZOEO-KXQOOQHDSA-N	1.6287	0.70372	0.017233	1.7636	[M+H]+	678.5046	4.49
PC(O-16:0/22:6)	QQQQNYAHSSIZBU-HIQXTUQZSA-N	0.32436	-1.6244	0.017504	1.7569	[M+H]+	792.5906	3.85
3-Methylhistidine	JDHILDINMRGULE-LURJTMIESA-N	1.7099	0.77394	0.01771	1.7518	[M+H]+	170.0917	9.1
SM(d18:1/18:0)	LKQLRGMMMAHREN-YJFXYUILSA-N	4.6239	2.2091	0.026926	1.5698	[M+H]+ _[M+Na]+	731.6044 _753.588	4.92
Ornithine	AHLPHDHHMVZTML-BYPYZUCNSA-N	1.8425	0.88165	0.031326	1.5041	[M+H]+	133.0961	9.27
SM(d18:1/24:1)	UYDFMJRVHYJLQZ-KVICREPESA-N	2.9102	1.5411	0.032995	1.4816	[M+H]+	813.6819	4.89
PHE-PHE	GKZIWHRNKRBEOH-UHFFFAOYSA-N	0.46692	-1.0988	0.032995	1.4816	[M+H]+	313.1546	5.57
CAR(16:0)	XOMRRQXKHMYMOC-OAQYLSRUSA-N	0.39103	-1.3546	0.032995	1.4816	[M+H]+	400.3417	3.14
LysoPC(15:0)	RJZVWDTYEWCUAR-JOCHJYFZSA-N	0.29578	-1.7574	0.032995	1.4816	[M+H]+ _[M+Na]+	482.3215 _504.307	5.27
Trigonelline	WWNNZCOKKKDOPX-UHFFFAOYSA-N	0.27292	-1.8734	0.032995	1.4816	[M+H]+	138.0544	7.2
Alanine	QNAYBMKLOCPYGJ-REOHCLBHSA-N	1.3097	0.38919	0.034779	1.4587	[M+H]+	90.0542	7.79
Kynurenine	YGPSJZOEDVAXAB-QMMMGPOBSA-N	1.2392	0.30943	0.043007	1.3665	[M+H]+	209.0914	6.62
Histidine	HNDVDQJCIGZPNO-YFKPBYRVSA-N	1.3423	0.42472	0.045202	1.3448	[M+H]+	156.0762	9.07
Proline	ONIBWKKTOPOVIA-BYPYZUCNSA-N	1.3413	0.42368	0.047436	1.3239	[M+H]+	116.0696	7.44
Lyso(PE18:1)	PYVRVRFVLRNJLY-MZMPXXGTSA-N	0.71988	-0.47418	0.047436	1.3239	[M+H]+	480.308	5.44
Methionine	FFEARJCKVFRZRR-BYPYZUCNSA-N	1.3041	0.38304	0.053444	1.2721	[M+H]+ _[M+N-NH3]+	150.0575 _133.030	7.16
Gal ceramide (d18:1/24:1)	WBOZIXHPUPAOIA-YEWIENRVSA-N	1.628	0.70311	0.060268	1.2199	[M+H]+	810.6806	1.46
PC(O-18:1/2:0)	NA	0.73524	-0.44372	0.060268	1.2199	[M+H]+	550.3851	5.01
LysoPC(18:0)	ATHVAWFAEPLPPQ-QPOMNCEOSA-N	0.70519	-0.50393	0.060268	1.2199	[M+H]+	506.3592	5.02

N6-Methyllysine	PQNASZJZHFPQLE-UHFFFAOYSA-N	1.3341	0.41584	0.077546	1.1104	[M+H]+	161.1275	8.99
Carnitine	PHIQHXFUZVPYII-ZCFIWIBFSA-N	1.2445	0.31559	0.0819	1.0867	[M+H]+ _[M+Na]+	162.1127 _184.093	7.48
PC(P-18:0/20:4)	FHHVIBPVBBRLOR-IYACIECVSA-N	1.3596	0.44317	0.082391	1.0841	[M+H]+	794.6058	4.3

Table S4: Significant annotated metabolites comparing WD neurologic to HC. Metabolites were selected by volcano plot with fold change (FC) >1.2 and FDR-adjusted *p* <0.1 indicating significance.

Metabolite Name/Identifier	InChI Key	FC	log2 (FC)	FDR *p*-value	-log10 (p)	Species	m/z	RT
Choline	OEYIOHPDSNJKLS-UHFFFAOYSA-N	3.9237	1.9722	2.15E-13	12.668	[M+H]+	104.107	5.12
Phenylalanine	COLNVLDHVKWLRT-QMMMGPOBSA-N	2.1497	1.1041	6.94E-09	8.1585	[M+H]+	166.086	6.61
2-Hydroxy phenethylamine	ULSIYEODSMZIPX-UHFFFAOYSA-N	2.1188	1.0833	6.94E-09	8.1585	[M+H-H2O]+	120.0804	6.61
S1P	DUYSYHSSBDVJSM-KRWOKUGFSA-N	0.60486	-0.7253	1.20E-06	5.921	[M+H]+	380.255	5.96
PS(18:0/20:4)	SVOUGFFDROZBJI-DNALCEECSA-N	0.31296	-1.6759	6.43E-05	4.1919	[M+H]+	812.543	5.4
LysoPC(P-18:0)	WBOMIOWRFSPZMC-AYICAFKVSA-N	0.58891	-0.7639	0.0002	3.6889	[M+H]+	482.324	5.42
Methionine	FFEARJCKVFRZRR-BYPYZUCNSA-N	1.5205	0.60458	0.00056	3.2522	[M+H]+ _[M+N-NH3]+	150.0575 _133.030	7.16
PC(18:1/14:0)	KIVAJCJTVPWSRJ-OQHNRNOKSA-N	1.6642	0.73486	0.00131	2.8839	[M+H]+	732.553	4.43
PC(14:0/18:0)	TYAQXZHDAGZOEO-KXQOOQHDSA-N	1.8995	0.9256	0.00215	2.6685	[M+H]+	734.569	4.45
LysoPC(24:0)	SKJMUADLQLZAGH-WJOKGBTCSA-N	0.73279	-0.4485	0.00259	2.5873	[M+H]+	608.463	5.03
LysoPC(14:0)	VXUOFDJKYGDUJI-OAQYLSRUSA-N	0.58288	-0.7787	0.00259	2.5873	[M+H]+	468.309	5.29
Kynurenine	YGPSJZOEDVAXAB-QMMMGPOBSA-N	1.3776	0.46218	0.00301	2.5221	[M+H]+	209.091	6.62
Tyrosine	OUYCCCASQSFEME-QMMMGPOBSA-N	1.5647	0.64589	0.00395	2.4036	[M+H]+	182.08	7.39
PC(18:3/18:3)	XXKFQTJOJZELMD-JICBSJGISA-N	0.57205	-0.8058	0.00395	2.4036	[M+H]+	778.537	4.18
LysoPC(18:1)	YAMUFBLWGFFICM-PTGWMXDISA-N	0.75887	-0.3981	0.00467	2.3308	[M+H]+	522.356	5.17
SM(d18:1/18:1)	NBEADXWAAWCCDG-QDDWGVBQSA-N	0.80369	-0.3153	0.00561	2.2514	[M+H]+	729.591	4.93
Histidine	HNDVDQJCIGZPNO-YFKPBYRVSA-N	1.5291	0.61269	0.01307	1.8837	[M+H]+	156.076	9.07
PC(P-18:0/20:4)	FHHVIBPVBBRLOR-IYACIECVSA-N	1.4379	0.52401	0.01663	1.7792	[M+H]+	794.606	4.3
PC(18:0/22:6)	FAUYAENFVCNTAL-PFFNLMTBSA-N	1.4297	0.51567	0.01887	1.7243	[M+H]+	834.601	4.31
CAR(12:0)	FUJLYHJROOYKRA-QGZVFWFLSA-N	0.35652	-1.488	0.01887	1.7243	[M+H]+	344.279	3.69
LysoPC(16:0).2	ASWBNKHCZGQVJV-HSZRJFAPSA-N	1.205	0.26901	0.0191	1.7191	[M+H]+	518.322	5.24
Glycocholic acid	RFDAIACWWDREDC-FRVQLJSFSA-N	2.6526	1.4074	0.01957	1.7085	[M+H]+ _[M+NH4]+	483.342 _466.315	5.89
SM(d18:1/12:0)	HZCLJRFPXMKWHR-FEBLJDHQSA-N	0.71532	-0.4834	0.01957	1.7085	[M+H]+	647.511	4.99
Ornithine	AHLPHDHHMVZTML-BYPYZUCNSA-N	1.9751	0.98194	0.02315	1.6355	[M+H]+	133.096	9.27
PC(P-18:0/22:6)	FAUYAENFVCNTAL-PFFNLMTBSA-N	1.5152	0.5995	0.02869	1.5423	[M+H]+	818.605	4.28
SM(d18:1/18:0)	LKQLRGMMMAHREN-YJFXYUILSA-N	3.4924	1.8042	0.03491	1.4571	[M+H]+ _[M+Na]+	731.6044 _753.588	4.92
LysoPC(P-16:0)	HTZINLFNXLXRBC-CQLBIITFSA-N	0.69518	-0.5246	0.0354	1.4511	[M+H]+	508.375	5.22
1-Methylnicotinamide	LDHMAVIPBRSVRG-UHFFFAOYSA-O	0.6415	-0.6405	0.0354	1.4511	[M+H-C4H8]+	137.07	6.02
4-Aminomethylcyclo hexanecarboxylic acid	GYDJEQRTZSCIOI-LJGSYFOKSA-N	0.62293	-0.6829	0.03789	1.4215	[M-H]-	158.117	6.62
7-a-OH-3-oxo-Cholest-4-en-26-oic acid	SATGKQGFUDXGAX-SLTBQWEQSA-N	1.2746	0.35006	0.03818	1.4182	[M+H]+	431.315	1.21
PC(16:1/16:1)	GPWHCUUIQMGELX-VHQDNGOZSA-N	0.74529	-0.4241	0.03884	1.4107	[M+H]+	730.537	4.43
Hippuric acid	QIAFMBKCNZACKA-UHFFFAOYSA-N	0.59177	-0.7569	0.03884	1.4107	[M+H]+	180.065	3.38
Alanine	QNAYBMKLOCPYGJ-REOHCLBHSA-N	1.2821	0.35849	0.04182	1.3787	[M+H]+	90.0542	7.79
Glycoursodeoxycholic acid	GHCZAUBVMUEKKP-TVQURETASA-N	2.3972	1.2614	0.04663	1.3313	[M+H-H2O]+	432.31	4.71

Carnitine	PHIQHXFUZVPYII-ZCFIWIBFSA-N	1.2558	0.32862	0.04663	1.3313	[M+H]+ _[M+Na]+	162.1127 _184.093	7.48
PC(O-16:0/20:1).1	NA	0.82328	-0.2805	0.04663	1.3313	[M+H]+	524.372	5.16
CAR(16:0)	XOMRRQXKHMYMOC-OAQYLSRUSA-N	0.49048	-1.0277	0.04663	1.3313	[M+H]+	400.342	3.14
Proline	ONIBWKKTOPOVIA-BYPYZUCNSA-N	1.2783	0.35426	0.04709	1.3271	[M+H]+	116.07	7.44
PC(16:0/14:0)	UIXXHROAQSBBOV-PSXMRANNSA-N	1.3549	0.43818	0.04726	1.3255	[M+H]+	706.538	4.46
LysoPC(15:0)	RJZVWDTYEWCUAR-JOCHJYFZSA-N	0.37956	-1.3976	0.04815	1.3174	[M+H]+ _[M+Na]+	482.3215 _504.307	5.27
PC(O-16:0/22:6)	TXHZYNSTTCIWMJ-RXSQUPBGSA-N	0.58631	-0.7703	0.05322	1.2739	[M+H]+	792.591	3.85
N6-Methyllysine	PQNASZJZHFPQLE-UHFFFAOYSA-N	1.4885	0.57382	0.0591	1.2284	[M+H]+	161.128	8.99
CAR(10:0)	LZOSYCMHQXPBFU-OAHLLOKOSA-N	0.58469	-0.7743	0.07324	1.1353	[M+H]+	316.248	4.11
SM(d18:1/24:1)	UYDFMJRVHYJLQZ-KVICREPESA-N	2.2895	1.195	0.07844	1.1055	[M+H]+	813.682	4.89
PE(16:0/18:1)	FHQVHHIBKUMWTI-OTMQOFQLSA-N	1.3741	0.45849	0.09024	1.0446	[M+H]+	718.537	4.58

Tabel S5: Significant annotated metabolites comparing WD hepatic to HC. Metabolites were selected by volcano plot with fold- change (FC) >1.2 and FDR-adjusted *p* <0.1 indicating significance.

Metabolite Name/Identifier	InChI Key	FC	log2 (FC)	FDR *p*-value	-log10 (p)	Species	m/z	RT
Choline	OEYIOHPDSNJKLS-UHFFFAOYSA-N	4.3574	2.1235	2.18E-13	12.661	[M+H]+	104.1066	5.12
Phenylalanine	COLNVLDHVKWLRT-QMMMGPOBSA-N	2.4547	1.2956	2.26E-08	7.646	[M+H]+	166.0862	6.61
2-Hydroxy phenethylamine	ULSIYEODSMZIPX-UHFFFAOYSA-N	2.415	1.2721	2.30E-08	7.6378	[M+H-H2O]+	120.0804	6.61
LysoPC(P-18:0)	WBOMIOWRFSPZMC-AYICAFKVSA-N	0.44348	-1.1731	4.53E-07	6.3443	[M+H]+	508.3748	5.22
LysoPC(24:0)	SKJMUADLQLZAGH-WJOKGBTCSA-N	0.69985	-0.51489	0.000182	3.7402	[M+H]+	608.4634	5.03
S1P	DUYSYHSSBDVJSM-KRWOKUGFSA-N	0.64404	-0.63478	0.000231	3.6359	[M+H]+	380.2552	5.96
PC(18:3/18:3)	XXKFQTJOJZELMD-JICBSJGISA-N	0.50274	-0.99211	0.000231	3.6359	[M+H]+	778.5374	4.18
LysoPC(14:0)	VXUOFDJKYGDUJI-OAQYLSRUSA-N	0.53095	-0.91336	0.000313	3.5043	[M+H]+	468.3086	5.29
PS(18:0/20:4)	FHHVIBPVBBRLOR-IYACIECVSA-N	0.41435	-1.2711	0.000313	3.5043	[M+H]+	812.5433	5.4
PC(18:1/14:0)	KIVAJCJTVPWSRJ-OQHNRNOKSA-N	1.8275	0.86988	0.000493	3.3073	[M+H]+	732.5534	4.43
SM(d18:1/18:1)	NBEADXWAAWCCDG-QDDWGVBQSA-N	0.7124	-0.48924	0.000764	3.1167	[M+H]+	729.5913	4.93
Histidine	HNDVDQJCIGZPNO-YFKPBYRVSA-N	1.9135	0.93624	0.001947	2.7106	[M+H]+	156.0762	9.07
LysoPC(18:1)	YAMUFBLWGFFICM-PTGWMXDISA-N	0.74108	-0.4323	0.001947	2.7106	[M+H]+	522.3559	5.17
Hippuric acid	QIAFMBKCNZACKA-UHFFFAOYSA-N	0.44641	-1.1636	0.003238	2.4897	[M+H]+	180.0645	3.38
PC(14:0/18:0)	TYAQXZHDAGZOEO-KXQOOQHDSA-N	2.1634	1.1133	0.00351	2.4547	[M+H]+	678.5046	4.49
Tyrosine	OUYCCCASQSFEME-QMMMGPOBSA-N	1.8955	0.92255	0.003674	2.4348	[M+H]+	182.0802	7.39
Methionine	FFEARJCKVFRZRR-BYPYZUCNSA-N	3.8412	1.9416	0.004076	2.3898	[M+H]+ _[M+N-NH3]+	150.0575 _133.0308	7.16
7-a-OH-3-oxo-Cholest-4-en-26-oic acid	SATGKQGFUDXGAX-SLTBQWEQSA-N	1.8093	0.8554	0.004566	2.3405	[M+H]+	431.3148	1.21
PC(16:1/16:1)	GPWHCUUIQMGELX-VHQDNGOZSA-N	0.67574	-0.56546	0.005196	2.2843	[M+H]+	730.5372	4.43
Kynurenine	YGPSJZOEDVAXAB-QMMMGPOBSA-N	1.5307	0.61421	0.006967	2.157	[M+H]+	209.0914	6.62
Betaine	KWIUHFFTVRNATP-UHFFFAOYSA-N	1.4629	0.54886	0.006967	2.157	[M+H]+	118.0863	6.98
PC(O-16:0/22:6)	QQQQNYAHSSIZBU-HIQXTUQZSA-N	0.40111	-1.3179	0.008002	2.0968	[M+H]+	792.5906	3.85
Ornithine	AHLPHDHHMVZTML-BYPYZUCNSA-N	2.5069	1.3259	0.008597	2.0656	[M+H]+	133.0961	9.27
LysoPC(15:0)	RJZVWDTYEWCUAR-JOCHJYFZSA-N	0.18868	-2.406	0.008981	2.0467	[M+H]+ _[M+Na]+	482.3215 _504.3075	5.27
PC(P-18:0/20:4)	FHHVIBPVBBRLOR-IYACIECVSA-N	1.5977	0.67603	0.009536	2.0206	[M+H]+	794.6058	4.3
PC(20:4/20:4)	LZLVZIFMYXDKCN-QJWFYWCHSA-N	0.34292	-1.5441	0.009536	2.0206	[M+H]+	830.5692	4
LysoPC(16:0).2	ASWBNKHCZGQVJV-HSZRJFAPSA-N	1.3432	0.42563	0.01187	1.9255	[M+H]+	518.3223	5.24
N6-Methyllysine	PQNASZJZHFPQLE-UHFFFAOYSA-N	2.0678	1.0481	0.013173	1.8803	[M+H]+	161.1275	8.99
Glycoursodeoxy cholic acid	GHCZAUBVMUEKKP-TVQURETASA-N	5.1039	2.3516	0.013505	1.8695	[M+H-H2O]+	432.3095	4.71
PC(16:0/14:0)	UIXXHROAQSBBOV-PSXMRANNSA-N	1.5046	0.5894	0.015233	1.8172	[M+H]+	706.5375	4.46
SM(d18:1/12:0)	HZCLJRFPXMKWHR-FEBLJDHQSA-N	0.7391	-0.43616	0.015233	1.8172	[M+H]+	647.5109	4.99
CAR(12:0)	FUJLYHJROOYKRA-QGZVFWFLSA-N	0.43623	-1.1969	0.015233	1.8172	[M+H]+	344.2791	3.69
Acetylcholine	OIPILFWXSMYKGL-UHFFFAOYSA-N	1.499	0.58399	0.017776	1.7502	[M+H]+	146.1168	7.52
Palmitoleoyl Ethanolamide	WFRLANWAASSSFV-FPLPWBNLSA-N	1.3211	0.40178	0.017776	1.7502	[M+H]+	298.274	1.02

Proline	ONIBWKKTOPOVIA-BYPYZUCNSA-N	1.4851	0.57054	0.018759	1.7268	[M+H]+	116.0696	7.44
5-Aminopentanoic acid	JJMDCOVWQOJGCB-UHFFFAOYSA-N	2.7402	1.4543	0.019844	1.7024	[M+H-H2O]+	100.0752	1.5
Alanine	QNAYBMKLOCPYGJ-REOHCLBHSA-N	1.3439	0.42646	0.019844	1.7024	[M+H]+	90.0542	7.79
CAR(10:0)	RFDAIACWWDREDC-FRVQLJSFSA-N	0.5259	-0.92714	0.021989	1.6578	[M+H]+	316.2478	4.11
Glycocholic acid	RFDAIACWWDREDC-FRVQLJSFSA-N	4.9625	2.3111	0.022623	1.6454	[M+H]+ _[M+NH4]+	483.342 _466.3154	5.89
PHE-PHE	GKZIWHRNKRBEOH-UHFFFAOYSA-N	0.60592	-0.72279	0.024145	1.6172	[M+H]+	313.1546	5.57
Lyso PAF C-16	VLBPIWYTPAXCFJ-UHFFFAOYSA-N	1.4482	0.53426	0.036462	1.4382	[M+H]+	482.3582	5.27
Trigonelline	WWNNZCOKKKDOPX-UHFFFAOYSA-N	138.0544	-1.7781	0.038987	1.4091	[M+H]+	138.0544	7.2
Acetylcarnitine	RDHQFKQIGNGIED-MRVPVSSYSA-O	1.3438	0.42632	0.042535	1.3713	[M+H]+	204.1234	6.95
PC(O-16:0/20:1).1	NA	0.82713	-0.27381	0.042535	1.3713	[M+H]+	524.3716	5.16
PE(O-18:1/20:4)	URPXXNCTXCOATD-FXMFQVEGSA-N	0.50772	-0.9779	0.050332	1.2982	[M+H]+	752.5604	3.81
PC(P-18:0/22:6)	TXHZYNSTTCIWMJ-RXSQUPBGSA-N	1.491	0.57628	0.051153	1.2911	[M+H]+	818.605	4.28
PC(O-16:0/20:4)	NA	1.2836	0.36024	0.084826	1.0715	[M+H]+	768.5897	4.33
3-Methylhistidine	JDHILDINMRGULE-LURJTMIESA-N	1.9118	0.93494	0.096339	1.0162	[M+H]+	170.0917	9.1
PI(18:0/20:4)	KRTOMQDUKGRFDJ-MWZLTEOLSA-N	0.82755	-0.27308	0.096339	1.0162	[M+H]+ _[M+NH4]+	904.5929 _887.5652	5.48

Table S6: Metabolite correlation analysis with choline. Correlation coefficients were calculated based on Pearson's correlation with p <0.05 indicating significance.

Metabolite	correlation	*t*-stat	*p*-value	FDR *p*-value
Phenylalanine	0.78285	10.75	1.07E-16	3.95E-15
LysoPC(14:0)	-0.67477	-7.8116	3.18E-11	7.84E-10
PC(14:0/18:0)	0.65672	7.4403	1.58E-10	2.92E-09
LysoPC(18:1)	-0.64136	-7.1423	5.67E-10	8.39E-09
PC(P-18:0/20:4)	0.6345	7.0138	9.82E-10	1.21E-08
PC(O-16:0/2:0).1	-0.62883	-6.9099	1.53E-09	1.62E-08
S1P	-0.62261	-6.798	2.46E-09	2.28E-08
Histidine	0.60254	6.4506	1.07E-08	8.79E-08
LysoPC(P-18:0)	-0.59826	-6.379	1.44E-08	1.07E-07
PC(18:1/14:0)	0.57975	6.0793	5.03E-08	3.23E-07
LysoPC(24:0)	-0.5791	-6.0691	5.24E-08	3.23E-07
Ornithine	0.56956	5.9205	9.67E-08	5.51E-07
Alanine	0.5419	5.509	5.14E-07	2.72E-06
PC(P-18:0/22:6)	0.53696	5.4383	6.82E-07	3.36E-06
PC(O-16:0/22:6)	-0.52538	-5.2755	1.30E-06	6.02E-06
PC(O-16:0/2:0).2	-0.51975	-5.198	1.77E-06	7.68E-06
CAR(12:0)	-0.51847	-5.1805	1.89E-06	7.77E-06
PS(18:0/20:4)	-0.50082	-4.9436	4.74E-06	1.84E-05
PC(O-16:0/20:4)	0.49988	4.9313	4.97E-06	1.84E-05
Tyrosine	0.49827	4.9101	5.39E-06	1.90E-05
Betaine	0.49619	4.883	5.98E-06	2.01E-05
Proline	0.48422	4.7285	1.07E-05	3.46E-05
Carnitine	0.48105	4.6882	1.25E-05	3.85E-05
SM(d18:1/18:1)	-0.47946	-4.6681	1.35E-05	3.99E-05
LysoPC(P-16:0)	-0.47674	-4.6337	1.53E-05	4.36E-05
Kynurenine	0.46938	4.5418	2.16E-05	5.91E-05
Methionine	0.46714	4.5141	2.39E-05	6.31E-05
PC(18:3/18:3)	-0.46356	-4.4699	2.81E-05	7.17E-05
LysoPC(15:0)	-0.45408	-4.3545	4.28E-05	0.00010555
PC(16:0/14:0)	0.444	4.2337	6.60E-05	0.00015762
PI(18:0/20:4)	-0.43925	-4.1775	8.06E-05	0.00018649
SM(d18:1/18:0)	0.42464	4.0074	0.0001464	0.00032829
CAR(16:0)	-0.41967	-3.9503	0.00017826	0.00038798
LysoPC(16:0).1	-0.41242	-3.868	0.00023618	0.00049934
PC(18:1/18:1)	0.40541	3.7892	0.00030829	0.0006337
PC(O-18:1/2:0)	-0.38708	-3.5868	0.00060209	0.0012042
Gal ceramide (d18:1/24:1)	0.38412	3.5546	0.00066844	0.0013017

Glycoursodeoxycholic acid	0.36504	3.3501	0.0012813	0.0024313
PC(18:0/22:6)	0.36292	3.3277	0.0013738	0.0025416
LysoPC(16:0).2	0.36162	3.314	0.0014339	0.002588
CAR(10:0)	-0.35951	-3.2917	0.0015362	0.0027067
Lyso(PE18:1)	-0.3479	-3.1705	0.0022248	0.0037199
Glycocholic acid	0.34752	3.1666	0.0022514	0.0037199
SMd(18:1/12:0)	-0.34737	-3.165	0.0022621	0.0037199
PC(16:1/16:1)	-0.34611	-3.152	0.0023528	0.0037291
PE(16:0/18:1)	0.34532	3.1438	0.0024113	0.0037291
PC(O-16:0/20:5)	-0.34522	-3.1428	0.0024189	0.0037291
CAR(18:1)	-0.33834	-3.072	0.0029874	0.0045116
Leucine	0.33095	2.9965	0.0037292	0.0055192
SMd(18:1/24:1)	0.31426	2.8284	0.0060357	0.0087577
PC(20:4/20:4)	-0.273	-2.4246	0.0178	0.02533
PC(18:0/18:1)	-0.27215	-2.4164	0.018174	0.025374
LysoPC(18:0)	-0.2604	-2.3044	0.02405	0.032958
PC(18:0/18:0)	0.2553	2.256	0.027064	0.036339
PE(O-18:1/20:4)	-0.2546	-2.2494	0.0275	0.036339
LysoPE(18:0)	-0.25156	-2.2208	0.02947	0.03826
Acetylcarnitine	0.23095	2.0281	0.046202	0.058947
Lyso PAF C-16	0.20636	1.802	0.075681	0.094922
PC(16:0/18:2)	0.19049	1.6579	0.10162	0.12534
Acetylcholine	0.18224	1.5836	0.11761	0.14267
PC(20:1/20:1)	0.11639	1.0012	0.32002	0.38196
PE(16:0/18:2)	0.098718	0.84759	0.39944	0.46918
Isoleucine	0.079209	0.6789	0.49935	0.57737
PC(16:0/18:1)	0.063271	0.54168	0.58969	0.67134
PC(16:0/22:6)	-0.060903	-0.52132	0.60372	0.6769
TMAO	0.036081	0.30848	0.7586	0.83785
Cholesterol	0.032133	0.27468	0.78434	0.85354
PC(16:0/18:0)	0.023331	0.1994	0.84251	0.90356
Tryptophan	0.020291	0.1734	0.86282	0.91212
PE(18:0/22:6)	0.017483	0.14939	0.88166	0.91891
PC(14:0/14:0)	0.015561	0.13297	0.89458	0.91943
Deoxycholic acid	-0.011273	-0.096324	0.92353	0.93618
PC(O-16:0/20:3)	0.0050072	0.042782	0.96599	0.96599

Table S7: Metabolite correlation analysis with phenylalanine. Correlations coefficients were calculated based on Pearson's correlation with $p < 0.05$ indicating significance.

Metabolite	Correlation	*t*-stat	*p*-value	FDR *p*-value
Choline	0.78285	10.75	1.07E-16	3.95E-15
Kynurenine	0.71661	8.7785	4.85E-13	1.20E-11
Ornithine	0.68024	7.929	1.91E-11	3.54E-10
LysoPC(P-18:0)	-0.67262	-7.7662	3.87E-11	5.73E-10
Histidine	0.6656	7.62	7.27E-11	8.20E-10
Alanine	0.66487	7.6051	7.76E-11	8.20E-10
PC(14:0/18:0)	0.6622	7.5506	9.81E-11	9.07E-10
Methionine	0.65379	7.3824	2.02E-10	1.66E-09
Tyrosine	0.64632	7.2367	3.78E-10	2.80E-09
Proline	0.64466	7.205	4.33E-10	2.92E-09
LysoPC(14:0)	-0.62219	-6.7904	2.54E-09	1.57E-08
LysoPC(18:1)	-0.60125	-6.4289	1.17E-08	6.66E-08
PC(O-16:0/2:0).1	-0.58175	-6.111	4.41E-08	2.33E-07
S1P	-0.57324	-5.9774	7.66E-08	3.78E-07
Betaine	0.56287	5.8184	1.47E-07	6.80E-07
PC(18:1/14:0)	0.5494	5.6179	3.32E-07	1.44E-06
Glycoursodeoxycholic acid	0.54624	5.5717	4.00E-07	1.64E-06
PC(O-16:0/2:0).2	-0.53322	-5.3853	8.42E-07	3.14E-06
PC(P-18:0/20:4)	0.5331	5.3837	8.48E-07	3.14E-06
Carnitine	0.51775	5.1707	1.96E-06	6.92E-06
LysoPC(P-16:0)	-0.51274	-5.1027	2.56E-06	8.62E-06
SM(d18:1/18:1)	-0.50177	-4.9562	4.52E-06	1.45E-05
PC(18:3/18:3)	-0.49334	-4.8458	6.89E-06	2.12E-05
PC(18:1/18:1)	0.49047	4.8086	7.94E-06	2.35E-05
Glycocholic acid	0.48672	4.7605	9.52E-06	2.71E-05
Leucine	0.48405	4.7263	1.08E-05	2.97E-05
PE(16:0/18:1)	0.47874	4.6589	1.40E-05	3.69E-05
LysoPC(15:0)	-0.47104	-4.5624	2.00E-05	5.10E-05
PC(16:0/14:0)	0.46886	4.5353	2.21E-05	5.45E-05
PC(O-16:0/20:4)	0.46517	4.4898	2.61E-05	6.24E-05
LysoPC(16:0).1	-0.4626	-4.4581	2.93E-05	6.78E-05
PC(O-18:1/2:0)	-0.45834	-4.4061	3.55E-05	7.96E-05
CAR(12:0)	-0.43876	-4.1718	8.23E-05	0.00017911
PC(16:1/16:1)	-0.42845	-4.0513	0.00012567	0.00026569
PS(18:0/20:4)	-0.42178	-3.9745	0.00016403	0.00033718
PC(P-18:0/22:6)	0.40446	3.7786	0.00031948	0.00063896
CAR(10:0)	-0.38385	-3.5517	0.00067482	0.0013141
LysoPC(24:0)	-0.38077	-3.5184	0.00075141	0.0014258
Gal ceramide (d18:1/24:1)	0.37736	3.4816	0.00084552	0.0015642

LysoPC(18:0)	-0.37017	-3.4046	0.0010796	0.0019486
PC(O-16:0/22:6)	-0.35908	-3.2872	0.0015576	0.0027444
PI(18:0/20:4)	-0.34916	-3.1836	0.0021388	0.0036808
LysoPC(16:0).2	0.33856	3.0742	0.0029679	0.0049914
SMd(18:1/24:1)	0.33167	3.0039	0.0036504	0.0060028
CAR(18:1)	-0.32369	-2.923	0.0046131	0.0074211
SM(d18:1/18:0)	0.31728	2.8585	0.0055434	0.008728
CAR(16:0)	-0.3157	-2.8428	0.005796	0.0089355
PC(20:4/20:4)	-0.29113	-2.6	0.011275	0.017028
SMd(18:1/12:0)	-0.28137	-2.5052	0.014468	0.021412
LysoPE(18:0)	-0.27456	-2.4396	0.017132	0.024858
PC(16:0/22:6)	-0.25211	-2.2259	0.029108	0.041423
PE(O-18:1/20:4)	-0.24841	-2.1911	0.031637	0.044173
PC(18:0/22:6)	0.23607	2.0757	0.041446	0.056796
Tryptophan	0.22615	1.9837	0.051055	0.068692
Acetylcholine	0.21979	1.9249	0.058133	0.076818
PC(O-16:0/20:5)	-0.21409	-1.8726	0.065131	0.084557
Isoleucine	0.21283	1.861	0.066768	0.085187
PE(16:0/18:2)	0.21107	1.845	0.069096	0.086662
PC(16:0/18:2)	0.20773	1.8145	0.073714	0.090914
Lyso PAF C-16	0.20283	1.7698	0.080942	0.098192
Lyso(PE18:1)	-0.19675	-1.7145	0.090671	0.10822
PC(20:1/20:1)	0.19255	1.6765	0.097909	0.115
TMAO	0.18835	1.6386	0.10561	0.12211
Acetylcarnitine	0.17263	1.4975	0.13859	0.15777
PC(18:0/18:0)	0.16592	1.4376	0.15483	0.1736
PC(18:0/18:1)	-0.1649	-1.4285	0.15743	0.17387
PC(14:0/14:0)	0.10626	0.91305	0.36422	0.39636
Cholesterol	0.10285	0.88344	0.3799	0.40743
PC(16:0/18:1)	-0.0815	-0.69866	0.48699	0.51207
PE(18:0/22:6)	0.080695	0.69172	0.49131	0.51207
PC(O-16:0/20:3)	0.073132	0.62651	0.53293	0.54774
Deoxycholic acid	0.054473	0.46611	0.64253	0.65133
PC(16:0/18:0)	-0.014654	-0.12522	0.9007	0.9007

Table S8: Metabolite correlation analysis with methionine. Correlations coefficients were calculated based on Pearson's correlation with *p* <0.05 indicating significance.

Metabolite	Correlation	*t*-stat	*p*-value	FDR *p*-value
Betaine	0.82558	12.5	8.11E-20	3.00E-18
Proline	0.74838	9.6404	1.18E-14	2.92E-13
Tyrosine	0.70885	8.5864	1.11E-12	2.06E-11
SM(d18:1/18:1)	-0.69699	-8.3046	3.77E-12	5.58E-11
Glycoursodeoxycholic acid	0.68654	8.0675	1.05E-11	1.30E-10
LysoPC(P-18:0)	-0.65772	-7.4603	1.45E-10	1.53E-09
Phenylalanine	0.65379	7.3824	2.02E-10	1.87E-09
Histidine	0.64291	7.1715	5.00E-10	4.11E-09
PC(16:0/14:0)	0.61806	6.7173	3.47E-09	2.57E-08
PC(14:0/18:0)	0.61513	6.6661	4.31E-09	2.90E-08
Kynurenine	0.596	6.3417	1.69E-08	1.04E-07
LysoPC(16:0).1	-0.58055	-6.0919	4.77E-08	2.72E-07
PC(18:3/18:3)	-0.55698	-5.73	2.11E-07	1.11E-06
LysoPC(16:0).2	0.55385	5.6834	2.54E-07	1.26E-06
PC(18:1/14:0)	0.53623	5.4278	7.11E-07	3.29E-06
PC(O-16:0/20:4)	0.53008	5.3412	1.00E-06	4.37E-06
PC(16:0/22:6)	-0.52137	-5.2202	1.62E-06	6.65E-06
PC(O-16:0/2:0).1	-0.51847	-5.1805	1.89E-06	7.36E-06
Glycocholic acid	0.51191	5.0914	2.68E-06	9.90E-06
PC(18:1/18:1)	0.50407	4.9866	4.02E-06	1.42E-05
S1P	-0.49904	-4.9203	5.18E-06	1.74E-05
Alanine	0.47497	4.6115	1.67E-05	5.36E-05
Ornithine	0.47308	4.5879	1.82E-05	5.61E-05
PC(16:0/18:1)	-0.46893	-4.5362	2.20E-05	6.52E-05
Choline	0.46714	4.5141	2.39E-05	6.65E-05
PC(P-18:0/20:4)	0.46668	4.5084	2.44E-05	6.65E-05
PI(18:0/20:4)	-0.466	-4.4999	2.52E-05	6.65E-05
PE(16:0/18:1)	0.46447	4.4812	2.70E-05	6.88E-05
Leucine	0.43841	4.1677	8.35E-05	0.00020599
PC(O-16:0/2:0).2	-0.42314	-3.9901	0.00015541	0.00037098
LysoPC(14:0)	-0.41801	-3.9315	0.0001902	0.00043984
Acetylcholine	0.41587	3.907	0.00020678	0.00046369
PC(14:0/14:0)	0.39262	3.6474	0.00049384	0.0010748
CAR(10:0)	-0.36295	-3.328	0.0013727	0.0029024
TMAO	0.34668	3.1579	0.0023114	0.0047511
Tryptophan	0.31885	2.8743	0.0053019	0.010394
Isoleucine	0.31861	2.8719	0.0053377	0.010394

PE(O-18:1/20:4)	-0.30846	-2.7706	0.0070917	0.013456
Carnitine	0.30317	2.7182	0.008194	0.015159
PC(18:0/18:0)	0.29153	2.604	0.011157	0.020137
Gal ceramide (d18:1/24:1)	0.29048	2.5937	0.011466	0.020202
PC(O-18:1/2:0)	-0.28399	-2.5306	0.013543	0.023306
PC(P-18:0/22:6)	0.27058	2.4015	0.018877	0.031135
Cholesterol	0.27046	2.4003	0.018934	0.031135
Acetylcarnitine	0.2416	2.1272	0.036779	0.059166
PC(20:1/20:1)	0.23464	2.0623	0.042736	0.067287
PC(20:4/20:4)	-0.2282	-2.0026	0.04894	0.075449
LysoPE(18:0)	-0.22699	-1.9913	0.050185	0.07579
CAR(12:0)	-0.22528	-1.9756	0.051983	0.076935
PC(O-16:0/22:6)	-0.21598	-1.89	0.062733	0.090437
PC(O-16:0/20:5)	-0.21448	-1.8762	0.064627	0.090437
PC(O-16:0/20:3)	0.21343	1.8666	0.065974	0.090437
LysoPC(15:0)	-0.21342	-1.8665	0.065994	0.090437
PC(16:1/16:1)	-0.20659	-1.804	0.075358	0.10139
LysoPC(18:1)	-0.19303	-1.6808	0.097067	0.12827
CAR(18:1)	-0.18175	-1.5792	0.11862	0.154
PC(16:0/18:0)	0.16337	1.4148	0.16138	0.20589
PC(16:0/18:2)	0.16152	1.3984	0.16622	0.20848
PE(18:0/22:6)	-0.15741	-1.3619	0.17743	0.21883
PS(18:0/20:4)	-0.15335	-1.3259	0.189	0.22927
PC(18:0/22:6)	-0.14168	-1.2228	0.22532	0.26893
Lyso PAF C-16	0.12484	1.075	0.2859	0.33582
Lyso(PE18:1)	0.12195	1.0498	0.29727	0.33921
LysoPC(P-16:0)	-0.12178	-1.0483	0.29796	0.33921
SMd(18:1/12:0)	0.099904	0.85787	0.39377	0.4415
PE(16:0/18:2)	0.094416	0.81031	0.42039	0.46431
LysoPC(24:0)	-0.088926	-0.76281	0.44804	0.48757
CAR(16:0)	-0.061345	-0.52512	0.60109	0.64465
SMd(18:1/24:1)	0.057931	0.4958	0.62153	0.65704
Deoxycholic acid	-0.040352	-0.34505	0.73105	0.76194
LysoPC(18:0)	-0.03866	-0.33056	0.74192	0.76253
SM(d18:1/18:0)	0.031214	0.26682	0.79036	0.80118
PC(18:0/18:1)	-0.0092433	-0.078979	0.93727	0.93727

Table S9: Pathway analysis results. Statistical *p*-values are determined by enrichment analysis and adjusted for multiple testing. Pathway impact is determined by pathway topology analysis. "Total" = total number of compounds in the pathway; "Hits" = matched number of metabolites from the current analysis; "Raw *p*-value" = original *p*-value calculated from the enrichment analysis; " Holm adjust" = *p*-value adjusted by Holm-Bonferroni method; "FDR *p*-value" = *p*-value adjusted using the false discovery rate; "Impact" = pathway impact value calculated from pathway topology analysis.

Pathway	Total	Expected	Hits	Raw *p*-value	-log(p)	Holm adjust	FDR *p*-value	Impact
Beta Oxidation of Very Long Chain Fatty Acids	13	0.63	2	1.28E-01	2.05E+00	1.00E+00	1.00E+00	0.05
Betaine Metabolism	18	0.87	2	2.16E-01	1.53E+00	1.00E+00	1.00E+00	0.14
Oxidation of Branched Chain Fatty Acids	22	1.07	2	2.90E-01	1.24E+00	1.00E+00	1.00E+00	0.00
Phospholipid Biosynthesis	25	1.21	2	3.45E-01	1.06E+00	1.00E+00	1.00E+00	0.09
Phenylalanine and Tyrosine Metabolism	25	1.21	2	3.45E-01	1.06E+00	1.00E+00	1.00E+00	0.22
Beta-Alanine Metabolism	26	1.26	2	3.63E-01	1.01E+00	1.00E+00	1.00E+00	0.00
Phosphatidylethanolamine Biosynthesis	13	0.63	1	4.79E-01	7.37E-01	1.00E+00	1.00E+00	0.00
Catecholamine Biosynthesis	14	0.68	1	5.04E-01	6.85E-01	1.00E+00	1.00E+00	0.00
Histidine Metabolism	35	1.70	2	5.16E-01	6.62E-01	1.00E+00	1.00E+00	0.24
Sphingolipid Metabolism	36	1.75	2	5.31E-01	6.62E-01	1.00E+00	1.00E+00	0.07
Carnitine Synthesis	16	0.78	1	5.52E-01	6.33E-01	1.00E+00	1.00E+00	0.00
Methionine Metabolism	39	1.89	2	5.76E-01	5.94E-01	1.00E+00	1.00E+00	0.04
Fatty Acid Metabolism	40	1.94	2	5.90E-01	5.52E-01	1.00E+00	1.00E+00	0.00
Phosphatidylcholine Biosynthesis	18	0.87	1	5.95E-01	5.28E-01	1.00E+00	1.00E+00	0.00
Mitochondrial β-Oxidation of Long Chain Saturated Fatty Acids	24	1.17	1	7.01E-01	3.55E-01	1.00E+00	1.00E+00	0.00
Glycine and Serine Metabolism	50	2.43	2	7.13E-01	3.38E-01	1.00E+00	1.00E+00	0.00
Ammonia Recycling	25	1.21	1	7.16E-01	3.34E-01	1.00E+00	1.00E+00	0.00
Porphyrin Metabolism	36	1.75	1	8.39E-01	1.76E-01	1.00E+00	1.00E+00	0.00
Tyrosine Metabolism	55	2.67	1	9.40E-01	6.17E-02	1.00E+00	1.00E+00	0.00
Tryptophan Metabolism	55	2.67	1	9.40E-01	6.17E-02	1.00E+00	1.00E+00	0.09
Bile Acid Biosynthesis	59	2.87	1	9.52E-01	4.97E-02	1.00E+00	1.00E+00	0.00
Arachidonic Acid Metabolism	65	3.16	1	9.65E-01	3.59E-02	1.00E+00	1.00E+00	0.00

Table S10: Subject characteristics by clinical presentation. Data are expressed as mean ± SD. Statistical difference between groups was calculated by one-way ANOVA, with *p*-value <0.05 indicating significance.

	Healthy Control (n=15)	WD Pre-clinical (n=12)	WD Neurologic (n=22)	WD Hepatic (n=26)	*p*-value
Male (%)	5 (33.3%)	5 (41.6%)	13 (59.0%)	12 (46.1%)	-
Female (%)	10 (66.6%)	7 (58.3%)	9 (40.9%)	14 (53.8)	
Age (years)	36.13 ± 9.20	31.25 ± 13.22	36.50 ± 11.47	33.08 ± 11.50	0.503
BMI (Kg/m$_2$)	23.52 ± 3.88	25.56 ± 3.30	26.01 ± 6.30	25.31 ± 4.47	0.480

Table S11: List of internal standards used for quality control. RT, retention time; m/z, mass-to-charge ratio.

Metabolite name	Species	m/z	RT
1_15N2-L-Arginine iSTD	[2M+H]+	177.1122	9.15
1_CUDA iSTD	[M+H]+	341.2809	1.15
1_D3-1-Methylnicotinamide iSTD	[M-H]-	141.0937	6.03
1_D3-Creatine iSTD	[M+H]+	135.0952	7.78
1_D3-Creatinine iSTD	[M+H]+	117.0846	4.88
1_D3-DL-Alanine iSTD	[M+H]+	93.0731	7.80
1_D3-DL-Aspartic acid iSTD	[M-H]-	137.0626	8.89
1_D3-DL-Glutamic acid iSTD	[M+H]+	151.0785	8.45
1_D3-Histamine, N-methyl- iSTD	[M+H]+	129.1207	7.10
1_D3-L-Carnitine iSTD	[M+H]+	165.131	7.47
1_D5-L-Glutamine iSTD	[M+H]+	152.1069	8.29
1_D9-Betaine iSTD	[M+H-	127.142	6.99
1_D9-Butyrobetaine iSTD	[M+H]+	155.174	7.45
1_D9-Caffeine iSTD	[M+H]+	204.1438	1.21
1_D9-Choline iSTD	[M+H]+	113.1627	5.10
1_D9-Crotonobetaine iSTD	[M+H]+	153.1582	7.53
1_D9-TMAO iSTD	[M+H]+	85.1316	5.39
1_Val-Tyr-Val iSTD	[M+H]+	380.2181	6.64

Table S12: List of primers for mouse gene expression.

Gene	Gene name	Primer	Sequence 5' to 3'	Exon-exon overlap	% Primer efficiency
Aldh7a1	aldehyde dehydrogenase family 7, member A1	F	CCTGGGACCCCAATATCCTC	Yes	100.5
		R	ACCTTGCCCCCATAGACCAC	No	
Bhmt	Betaine-homocysteine methyltransferase 2	F	TTGGATTGGAACCCCGAGT	Yes	104.0
		R	CCTGATGTGGTAGGGCTCAAA	No	
Bhmt2	Betaine-homocysteine methyltransferase	F	GAAAGAGGGCCTCAGAGATGC	No	101.5
		R	GGATACTCTGGGAGGTCCACAA	No	
Chdh	Choline dehydrogenase	F	GAAGCCCCGACAGCTATTCC	No	101.8
		R	ACTTCCAGACAACCCCAGCA	No	
Chpt1	Choline phosphotransferase 1	F	AAGCACCGGAACAGGTTCAA	Yes	101.7
		R	CATGAAACAGCAGCAGCAAGA	No	
Gapdh	glyceraldehyde-3-phosphate dehydrogenase	F	GAAGCTTGTCATCAACGGGAAG	No	102.1
		R	TTTGATGTTAGTGGGGTCTCGC	No	
Mat1a	methionine adenosyltransferase I, alpha	F	TCTGTCCCATACTCACCTCTTCAG	No	98.4
		R	TGCCCTGAGGGTAGAAGGC	No	
Mat2a	methionine adenosyltransferase II, alpha	F	CAGGAGACCAGGGTTTGATGTT	Yes	95.8
		R	GCGTAACCAAGGCAATGTACC	No	
Mtr	methionine synthase	F	CTGCAGATGTGGCCAGAAAAG	No	98.9
		R	CAGCCACAAACCTCTTGACTCC	Yes	
Pcyt1a	Phosphate cytidylyltransferase 1, choline, alpha isoform	F	GAAGAGCATCGACCTCATCCA	No	101.3
		R	CTTCAGCATGTGCTTCAGTGC	Yes	
Pemt	phosphatidylethanolamine N-methyltransferase	F	TACTTCCTGGGCCTTGCATTC	No	100.1
		R	ACGCTGAAGGGAAATGTGGTC	No	

Figure S3: Features view showing distribution before and after normalization. Density plots represent all features. Boxplots display distribution for only 50 features due to limited space.

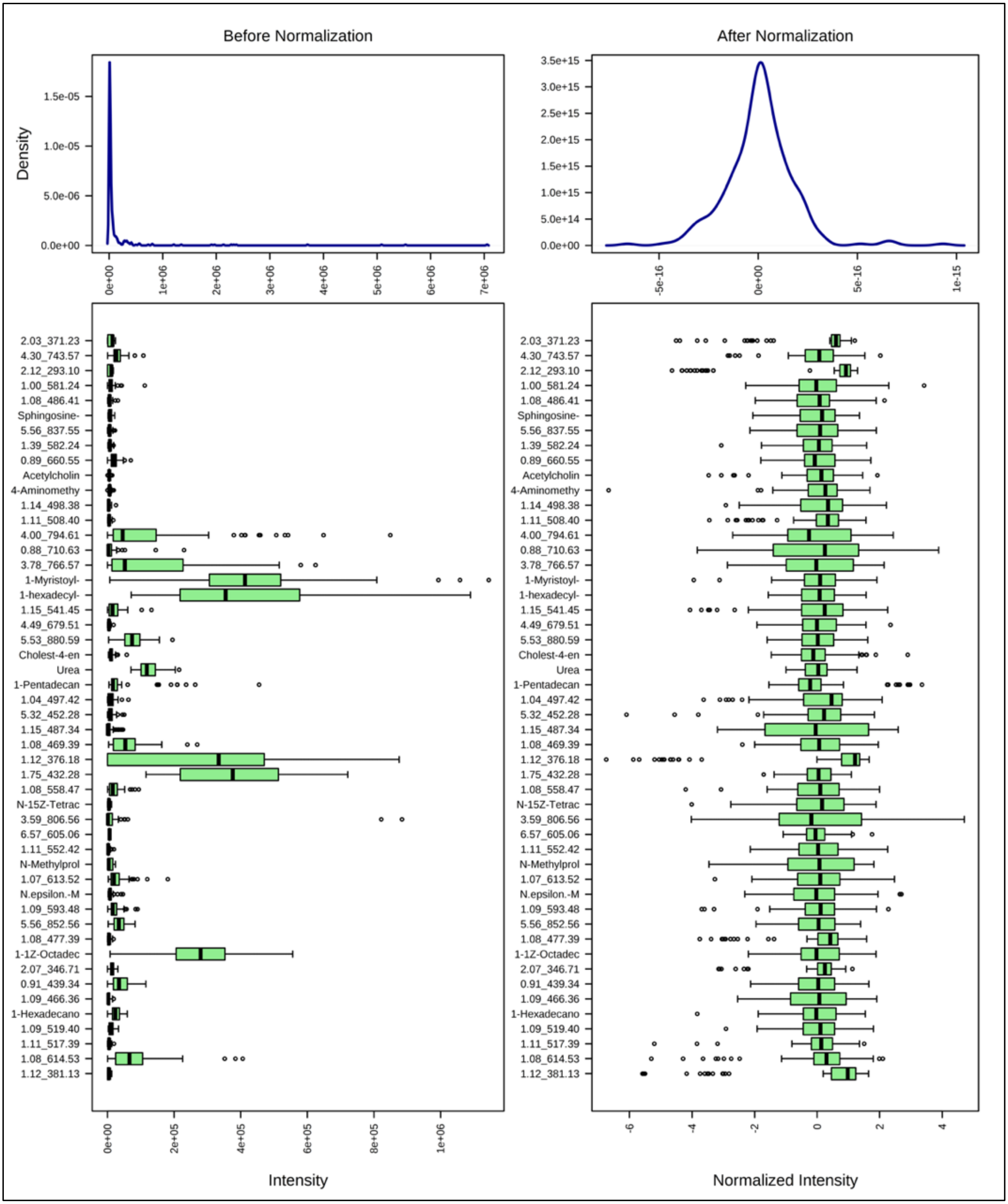

Supplement: Supplementary file 1 [file ijms-20-05937-s001.zip › Supplements/IJMS-Supplemental-White.pdf]
